# MARTX Toxin-Stimulated Interplay between Human Cells and Vibrio vulnificus

**DOI:** 10.1128/mSphere.00659-20

**Published:** 2020-08-12

**Authors:** Byoung Sik Kim, Jong-Hwan Kim, Sanghyeon Choi, Shinhye Park, Eun-Young Lee, Serry Koh, Choong-Min Ryu, Seon-Young Kim, Myung Hee Kim

**Affiliations:** a Infection and Immunity Research Laboratory, Metabolic Regulation Research Center, Korea Research Institute of Bioscience and Biotechnology, Daejeon, South Korea; b Department of Food Science and Engineering, ELTEC College of Engineering, Ewha Womans University, Seoul, South Korea; c Department of Functional Genomics, University of Science and Technology, Daejeon, South Korea; d Personalized Genomic Medicine Research Center, Korea Research Institute of Bioscience and Biotechnology, Daejeon, South Korea; e Infectious Disease Research Center, Korea Research Institute of Bioscience and Biotechnology, Daejeon, South Korea; University of Kentucky

**Keywords:** siderophore, MARTX toxin, *Vibrio vulnificus*, dual-RNA sequencing, iron limitation

## Abstract

To understand toxin-stimulated host-pathogen interactions, we performed dual-transcriptome sequencing experiments using human epithelial (HT-29) and differentiated THP-1 (dTHP-1) immune cells infected with the sepsis-causing pathogen Vibrio vulnificus (either the wild-type [WT] pathogen or a multifunctional-autoprocessing repeats-in-toxin [MARTX] toxin-deficient strain). Gene set enrichment analyses revealed MARTX toxin-dependent responses, including negative regulation of extracellular related kinase 1 (ERK1) and ERK2 (ERK1/2) signaling and cell cycle regulation in HT-29 and dTHP-1 cells, respectively. Further analysis of the expression of immune-related genes suggested that the MARTX toxin dampens immune responses in gut epithelial cells but accelerates inflammation and nuclear factor κB (NF-κB) signaling in immune cells. With respect to the pathogen, siderophore biosynthesis genes were significantly more highly expressed in WT V. vulnificus than in the MARTX toxin-deficient mutant upon infection of dTHP-1 cells. Consistent with these results, iron homeostasis genes that limit iron levels for invading pathogens were overexpressed in WT V. vulnificus-infected dTHP-1 cells. Taken together, these results suggest that MARTX toxin regulates host inflammatory responses during V. vulnificus infection while also countering host defense mechanisms such as iron limitation.

**IMPORTANCE** V. vulnificus is an opportunistic human pathogen that can cause life-threatening sepsis in immunocompromised patients via seafood poisoning or wound infection. Among the toxic substances produced by this pathogen, the MARTX toxin greatly contributes to disease progression by promoting the dysfunction and death of host cells, which allows the bacteria to disseminate and colonize the host. In response to this, host cells mount a counterattack against the invaders by upregulating various defense genes. In this study, the gene expression profiles of both host cells and V. vulnificus were analyzed by RNA sequencing to gain a comprehensive understanding of host-pathogen interactions. Our results suggest that V. vulnificus uses the MARTX toxin to subvert host cell immune responses as well as to oppose host counterattacks such as iron limitation.

## Abstract (precis)

V. vulnificus is an opportunistic human pathogen that can cause life-threatening sepsis in immunocompromised patients via seafood poisoning or wound infection. Among the toxic substances produced by this pathogen, the MARTX toxin greatly contributes to disease progression by promoting the dysfunction and death of host cells, which allows the bacteria to disseminate and colonize the host. In response to this, host cells mount a counterattack against the invaders by upregulating various defense genes. In this study, the gene expression profiles of both host cells and V. vulnificus were analyzed by RNA sequencing to gain a comprehensive understanding of host-pathogen interactions. Our results suggest that V. vulnificus uses the MARTX toxin to subvert host cell immune responses as well as to oppose host counterattacks such as iron limitation.

## INTRODUCTION

Upon infection, invading pathogens express virulence factors such as exotoxins to affect and disrupt the physiology and protective mechanisms of the host (1, 2). In response to infection, host cells produce numerous defense molecules, including cytokines/chemokines and antimicrobial agents (3). This results in turn in changes to the physiology and gene expression of the invading pathogen (4).

Microarray and tiling array analyses have been used to investigate global changes in gene expression profiles during infection. With improvements in sequencing technologies, however, transcriptome sequencing (also known as cDNA sequencing or RNA sequencing) became widely used in gene expression profiling experiments (5, 6). Further developments in sequencing technologies and bioinformatics analysis tools have significantly reduced the cost of RNA sequencing and helped researchers overcome limitations such as sample heterogeneity (7). Dual-transcriptome sequencing, which allows researchers to analyze the gene expression profiles of coexisting organisms simultaneously, has been used to examine host-pathogen interactions (8). Examples include Salmonella enterica serovar Typhimurium, Streptococcus pneumoniae, and Pseudomonas aeruginosa (9–11).

Vibrio vulnificus is a halophilic Gram-negative bacterium present in marine and estuarine environments worldwide. As an opportunistic pathogen, it can cause severe diseases such as life-threatening septicemia or necrotizing fasciitis in immunocompromised patients, including the elderly, or in individuals with hepatitis, hemochromatosis, or diabetes. People are infected by V. vulnificus mostly through either consumption of contaminated seafood or exposure of open wounds to contaminated seawater (12, 13). Among various virulence factors produced by this pathogen, multifunctional-autoprocessing repeats-in-toxin (MARTX) toxin is the primary exotoxin facilitating colonization and dissemination of V. vulnificus in orogastric as well as subcutaneous infection models in mice (14–19).

The multifunctionality of the MARTX toxin makes it critical for V. vulnificus pathogenesis (20, 21). Multiple effector domains located in the central region of the toxin exhibit various activities after release into host subcellular compartments, including the cytosol (22–24). For instance, the Rho inactivation domain (RID) disrupts the cell cytoskeleton via inactivation of Rho family proteins (25, 26), while the makes caterpillars floppy-like domain (MCF) causes Golgi dispersion and cell shrinking via modification of an unknown target(s) (24), and the Ras/Rap1-specific endopeptidase domain (RRSP) interrupts central signaling pathways via Ras/Rap1 cleavage (27, 28). Other effector domains, such as a domain of unknown function at the first position (DUF1) and an alpha/beta hydrolase domain (ABH), have been found in the MARTX toxin of V. vulnificus clinical isolates (21).

Since the MARTX toxin of V. vulnificus has been shown to promote colonization of the pathogen in the host gut (14), we hypothesized that it would dampen host immune responses at an early stage of infection, when the pathogen interacts with gut epithelial cells. Meanwhile, the amino- and carboxyl-terminal repeated sequence-containing domains of the toxin are thought to lyse infected host cells by forming a pore-like structure in the host plasma membrane (29). Indeed, the toxin is critical for dissemination of the pathogen to the bloodstream and to other organs where uncontrolled septic responses occur (12, 14). Therefore, we also hypothesized that the toxin would robustly enhance proinflammatory responses in host immune cells. Consistent with the cytotoxic/cytopathic activities of the toxin, studies have shown toxin-specific immune responses in diverse host cells and animal models (30–32). Nevertheless, none of these studies have compared MARTX toxin-mediated inflammatory responses in different types of host cells or concurrently analyzed the responses that simultaneously occur in the pathogen during infection.

In this study, we used dual-transcriptome sequencing to monitor gene expression in both V. vulnificus pathogens and human host cells. Specifically, human colorectal adenocarcinoma cells (HT-29) and differentiated THP-1 (dTHP-1) human monocytes were infected *in vitro* with either wild-type (WT) V. vulnificus or a mutant strain deficient in the MARTX toxin. Some cell type-specific responses occurred only during infection with WT V. vulnificus but not with the MARTX-deficient strain. In parallel, V. vulnificus also showed MARTX toxin-dependent gene expression patterns. Notably, siderophore biosynthetic genes were more highly expressed in the WT strain than in the MARTX-deficient strain during infection of dTHP-1 cells. Differential regulation of host genes related to iron homeostasis was also observed. These results suggest that V. vulnificus opposes host defense mechanisms while exerting cytotoxic/cytopathic effects via the MARTX toxin.

## RESULTS

### MARTX toxin-dependent gene expression profiles.

**FIG 1:**
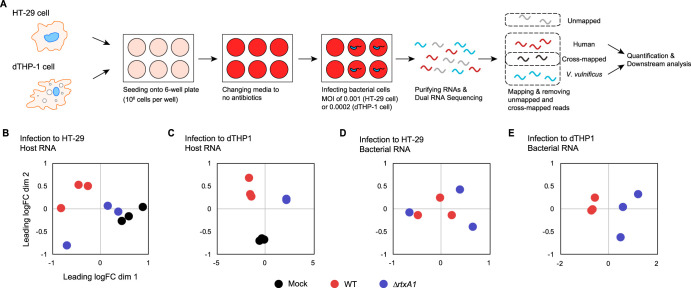
MARTX toxin-specific gene expression changes during infection. (A) Schematic representation of the experimental design. (B and C) Multidimensional scaling (MDS) plots of mock-treated, WT V. vulnificus-infected, and *ΔrtxA1* V. vulnificus-infected HT-29 cells (B) or dTHP-1 cells (C). (D and E) MDS plots of WT and *ΔrtxA1* V. vulnificus during HT-29 cell infection (D) or dTHP-1 cell infection (E).

To examine MARTX toxin-specific host responses, we used the clinical strain V. vulnificus MO6-24/O (WT) and an isogenic mutant deficient in the MARTX toxin (*ΔrtxA1*). Phosphate-buffered saline (PBS) was used as a mock control. Two different types of human cells, HT-29 cells, a representative gut epithelial cell line, and dTHP-1 cells, a representative immune cell line, were used as model host cells. At 3 or 6 h postinfection (h.p.i.), total RNA from the samples was purified, converted to a cDNA library, sequenced, and mapped to the human and V. vulnificus genomes to sort specifically mapped reads from unmapped or cross-mapped reads (Fig. 1A; see also Fig. S1A and B in the supplemental material). The mapped reads were further checked, and any reads corresponding to ribosomal RNAs (rRNAs) or tRNAs were removed from downstream analyses (Fig. S1A and C). It should be noted that the acute pathogen V. vulnificus lyses host cells very quickly using exotoxins like MARTX toxin (15, 17), and thus we used a low multiplicity of infection (MOI) to keep the host cells alive while monitoring transcriptional changes (see Materials and Methods).

10.1128/mSphere.00659-20.1 FIG S1 Analysis method and preprocessing results of the dual-RNA sequencing. (A) The bioinformatic analysis pipeline used for dual-RNA-seq analysis. (B) The percentage of reads mapped to human or *Vibrio* cells or unmapped, multimapped, or cross-mapped reads. (C) The percentage of reads corresponding to mRNAs, rRNAs, and tRNAs from human or V. vulnificus cells. Download FIG S1, PDF file, 0.2 MB. Copyright © 2020 Kim et al. 2020 Kim et al. This content is distributed under the terms of the Creative Commons Attribution 4.0 International license.

The number of sorted reads in each sample was high enough for transcriptome analysis of host cells (see Table S1A in the supplemental material) (33). For bacterial cells, however, only the 6 h.p.i. samples showed more than one million reads, and thus these samples were analyzed further (Table S1A). We assessed MARTX toxin-dependent changes in the overall gene expression profiles of both host cells and pathogens. We did not observe any significant differences among the mock control, WT-infected, and *ΔrtxA1* mutant-infected groups in the overall gene expression profiles of the 3 h.p.i. samples (data not shown). This was presumably because a very low MOI was used, as mentioned above, and 3 h was not enough for the pathogen to cause toxin-dependent changes in host gene expression. Thus, we mainly focused on the 6 h.p.i. samples in the following analyses.

10.1128/mSphere.00659-20.5 TABLE S1 The number of reads for each sample and list of immune-related genes used in this study. Download Table S1, PDF file, 0.1 MB. Copyright © 2020 Kim et al. 2020 Kim et al. This content is distributed under the terms of the Creative Commons Attribution 4.0 International license.

In a multidimensional scaling (MDS) plot, the transcriptomes of the HT-29 samples clustered into different groups according to the strain used for infection (mock control, WT, or *ΔrtxA1* mutant) (Fig. 1B). In particular, the WT-infected HT-29 cells formed a cluster that was distinct from the partially overlapping mock-treated and *ΔrtxA1* mutant-infected groups (Fig. 1B). Differences among the three groups were more evident in the dTHP-1 cells. In this case, the biological replicates for each sample were clearly clustered and formed three distinct groups in an MDS plot (Fig. 1C). Collectively, these results clearly reveal MARTX toxin-dependent gene expression changes in host cells and that these changes were more profound in the dTHP-1 immune cells than in the HT-29 epithelial cells.

On the pathogen side, a clear clustering of WT and *ΔrtxA1* V. vulnificus was observed upon infection of dTHP-1 cells but not HT-29 cells (Fig. 1D and E). The reason for this absence of sample clustering in HT-29 cells is not clear. Nonetheless, these results suggest that the MARTX toxin may play a role in opposing host defense mechanisms in immune cells. These results also indicate that dTHP-1 cells respond to the potent MARTX toxin more strongly than HT-29 cells.

### DEGs in V. vulnificus-infected HT-29 cells.

Next, we compared gene expression results between the mock-treated and WT-infected HT-29 cells and between the mock-treated and *ΔrtxA1* mutant-infected HT-29 cells. To focus on genes showing significant differences, we applied statistical cutoffs of a |fold change (FC)| level of ≥1.5 and a false-discovery rate (FDR) of ≤0.05. As shown in Fig. S2A, a total of 1,000 of the genes were differentially expressed genes (DEG) (716 upregulated and 284 downregulated) in the WT-infected HT-29 cells compared with the mock-treated controls. In contrast, only 155 genes were differentially expressed (129 upregulated and 26 downregulated) in the *ΔrtxA1* mutant-infected HT-29 cells compared with the mock-treated cells (Fig. S2B).

10.1128/mSphere.00659-20.2 FIG S2 Volcano plots of the DEGs in V. vulnificus-infected HT-29 and dTHP-1 cells. (A) DEGs in WT V. vulnificus-infected HT-29 cells compared with the mock-treated cells. (B) DEGs in *ΔrtxA1* V. vulnificus-infected HT-29 cells compared with mock-treated cells. (C) The negative regulation of the ERK1/2 pathway in WT V. vulnificus-infected HT-29 cells was confirmed by Western blotting. (D) DEGs in WT V. vulnificus-infected dTHP-1 cells compared with mock-treated cells. (E) DEGs in *ΔrtxA1* V. vulnificus-infected dTHP-1 cells compared with mock-treated cells. (F) The significant overexpression of PLK1 in WT V. vulnificus-infected dTHP-1 cells was confirmed by Western blotting. Blue and yellow dots indicate downregulated and upregulated genes, respectively, in the WT-infected cells (A and D) and *ΔrtxA1* mutant-infected cells (B and E) compared with mock-treated controls. FDR, false-discovery rate. p-ERK1/2, phospho-ERK1/2. Download FIG S2, PDF file, 2.7 MB. Copyright © 2020 Kim et al. 2020 Kim et al. This content is distributed under the terms of the Creative Commons Attribution 4.0 International license.

**FIG 2:**
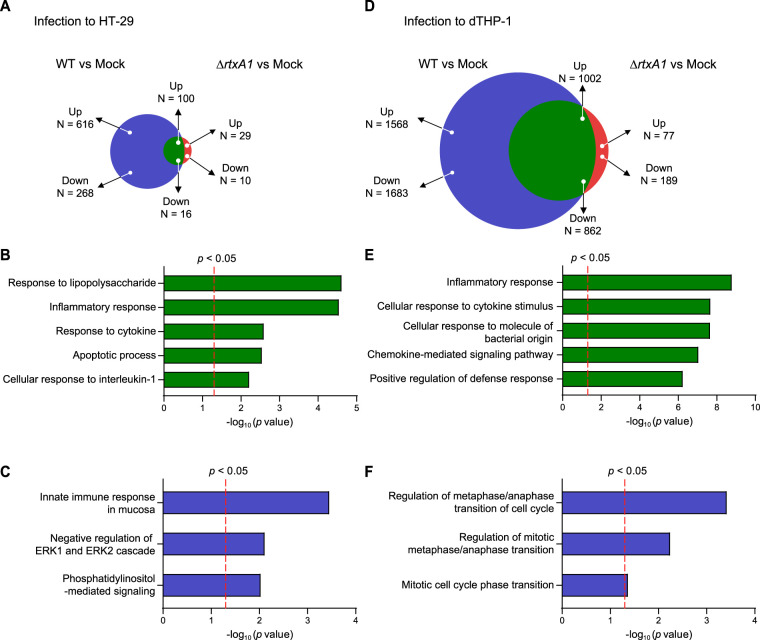
Differentially expressed genes (DEGs) in V. vulnificus-infected HT-29 and dTHP-1 cells. (A) Common and specific DEGs in WT- or *ΔrtxA1* V. vulnificus-infected HT-29 cells. (B) Gene sets enriched in the common DEGs shown in panel A. (C) Gene sets enriched in the DEGs specific for WT V. vulnificus-infected HT-29 cells. (D) Common and specific DEGs in WT- or *ΔrtxA1* V. vulnificus-infected dTHP-1 cells. (E) Gene sets enriched in the common DEGs shown in panel D. (F) Gene sets enriched in the DEGs specific for WT V. vulnificus-infected dTHP-1 cells. Red vertical dotted lines indicate the cutoff *P* value for significance (<0.05).

Among these differentially expressed genes (DEGs), 116 genes were upregulated (100 genes) or downregulated (16 genes) in both the WT- and *ΔrtxA1* mutant-infected HT-29 cells (Fig. 2A). Analysis performed with the Database for Annotation, Visualization, and Integrated Discovery (DAVID), a gene set enrichment analysis (GSEA) tool (34, 35), revealed enrichment of “response to lipopolysaccharide,” “inflammatory response,” “response to cytokine,” “apoptotic process,” and “cellular response to interleukin-1” (IL-1) gene sets (Fig. 2B). This is not surprising because both the WT and *ΔrtxA1* V. vulnificus strains produce lipopolysaccharide, which triggers numerous immune responses.

Notably, 884 genes were specifically upregulated (616 genes) or downregulated (268 genes) in WT-infected cells but not *ΔrtxA1* mutant-infected HT-29 cells compared with the mock-treated control (Fig. 2A), suggesting that they are MARTX toxin-specific DEGs. Enriched gene sets included “innate immune response in mucosa,” “negative regulation of extracellular related kinase 1 (ERK1) and ERK2 cascade,” and “phosphatidylinositol-mediated signaling” (Fig. 2C). To validate these results, we examined whether ERK1/2 activation was affected by the MARTX toxin and found that phospho-ERK1/2 levels were significantly lower in WT-infected HT-29 cells than in *ΔrtxA1* mutant-infected cells (Fig. S2C). These results correlate with the results of previous studies in which the MARTX toxin effector domain ABH showed phosphatidylinositol 3-phosphate (PI3P)-specific phospholipase A1 activity (36) and MARTX significantly downregulated ERK signaling (37). The gene expression analyses were also consistent with previous reports that MARTX toxin causes systemic pathogenicity via various cytopathic/cytotoxic functions of its effector domains (24).

### DEGs in V. vulnificus-infected dTHP-1 cells.

DEGs in dTHP-1 cells were analyzed upon V. vulnificus infection as described above. When the same statistical cutoffs of |FC| of ≥1.5 and FDR of ≤0.05 were applied, much higher numbers of DEGs were observed than were seen in the infected HT-29 cells. A total of 5,115 genes (2,570 upregulated and 2,545 downregulated) were identified as DEGs in a comparison of the mock-treated and WT-infected dTHP-1 cells (Fig. S2D). Comparing the mock-treated and *ΔrtxA1* mutant-infected dTHP-1 cells, there were a total of 2,130 DEGs (1,079 upregulated and 1,051 downregulated) (Fig. S2E). These results further indicate that the dTHP-1 cells responded more strongly to the pathogen than the HT-29 cells.

Similarly to the analyses of the HT-29 cells described above, common DEGs (1,002 upregulated and 862 downregulated; Fig. 2D) in both WT- and *ΔrtxA1* mutant-infected dTHP-1 cells compared with the mock-treated control were subjected to GSEA. Similarly, gene sets common in Gram-negative pathogen infection, including those in the categories “inflammatory response,” “cellular response to cytokine stimulus,” “cellular response to molecule of bacterial origin,” “chemokine-mediated signaling pathway,” and “positive regulation of defense response,” were significantly enriched (Fig. 2E).

Next, we analyzed the MARTX toxin-specific DEGs in infected dTHP-1 cells (1,568 upregulated and 1,683 downregulated; Fig. 2D). Intriguingly, the expression levels of multiple gene sets related to cell cycle regulation, such as “regulation of metaphase/anaphase transition of cell cycle,” “regulation of mitotic metaphase/anaphase transition,” and “mitotic cell cycle phase transition,” were significantly enriched (Fig. 2F). Remarkably, polo-like kinase 1 (PLK1), which is involved in cell cycle regulation, mainly during mitosis (38), was overexpressed in WT-infected but not *ΔrtxA1* mutant-infected dTHP-1 cells (Fig. S2F). These results suggest that MARTX toxin may dysregulate host cell proliferation to maximize its cytotoxicity. It is worth noting that the MARTX effector domain DUF1 has been shown to directly interact with the host cell protein prohibitin 1 (39). Since prohibitin regulates the cell cycle (40), the DUF1 effector domain may be responsible for the observed enrichment of gene sets related to proliferation.

### Immune-related DEGs in HT-29 cells.

Although some cellular responses were highlighted via the global GSEAs described above, we further analyzed the regulation of specific genes to obtain physiological clues about infected host cell responses. Among the DEGs, we focused on immune-related genes for further analysis. For this analysis, a focused gene list (202 genes; Table S1B) was generated by referring to the product information supplied for commercially available gene expression array kits (an antibacterial response RT^2^ profiler PCR array kit and an innate and adaptive immune responses RT^2^ profiler PCR array kit; Qiagen) and to *Vibrio*-responsive gene data reported in previous studies (31, 41).

**FIG 3:**
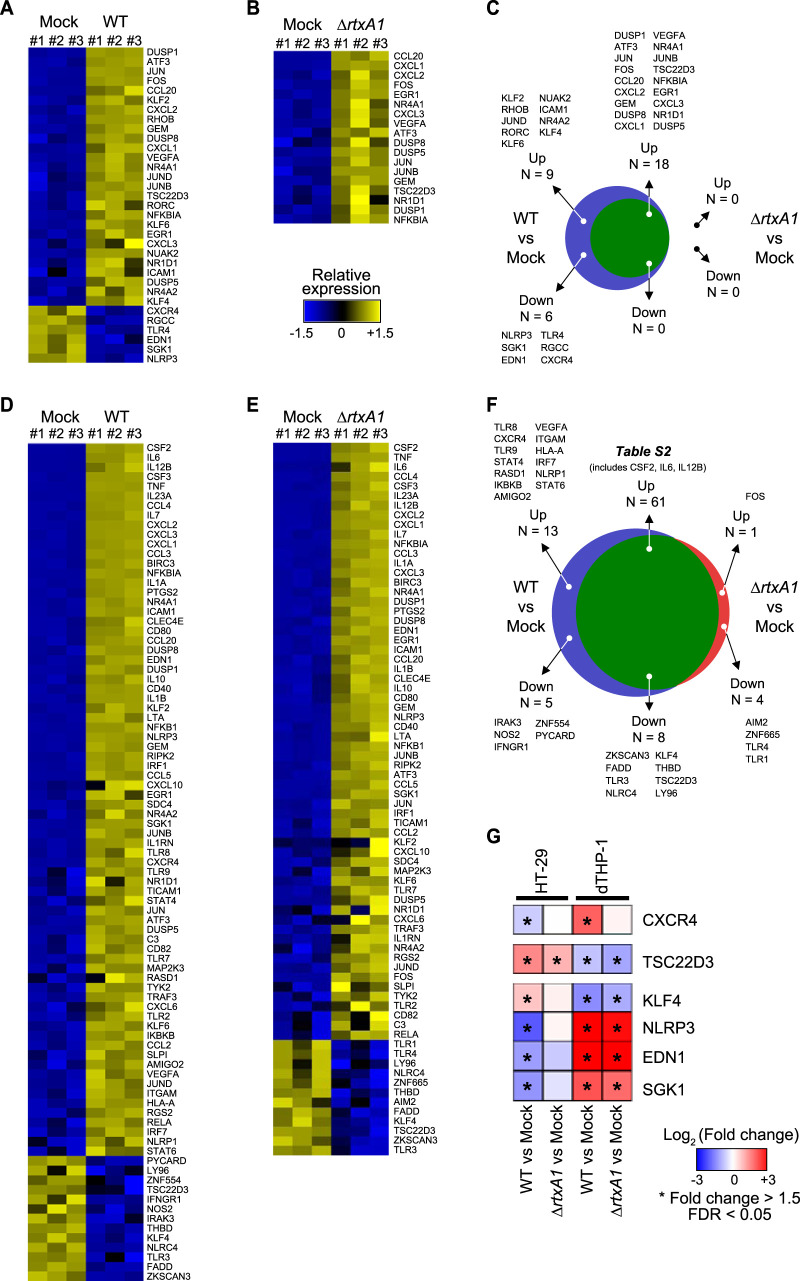
Immune-related DEGs in HT-29 and dTHP-1 cells. (A) Immune-related DEGs in WT V. vulnificus-infected HT-29 cells compared with mock-treated cells. (B) Immune-related DEGs in *ΔrtxA1* V. vulnificus-infected HT-29 cells compared with mock-treated cells. (C) Immune-related DEGs in common in or specific to WT- or *ΔrtxA1* V. vulnificus-infected HT-29 cells. (D) Immune-related DEGs in the WT V. vulnificus-infected dTHP-1 cells compared with mock-treated cells. (E) Immune-related DEGs in *ΔrtxA1* V. vulnificus-infected dTHP-1 cells compared with mock-treated cells. (F) Immune-related DEGs in common in or specific to either WT- or *ΔrtxA1* V. vulnificus-infected dTHP-1 cells. Common upregulated immune genes in WT- and *ΔrtxA1* mutant-infected dTHP-1 cells are shown in Table S2. (G) Some immune-related genes showed opposite expression patterns in the two types of host cells upon V. vulnificus infection. An asterisk (*) represents a statistically significant change in gene expression (|fold change| level of ≥1.5 and false-discovery rate [FDR] of <0.05).

Among the 202 genes, 27 and 6 genes were significantly upregulated or downregulated, respectively, in HT-29 cells upon WT V. vulnificus infection compared with the mock control (Fig. 3A). Remarkably, *DUSP1* (encoding dual-specificity protein phosphatase 1), *ATF3* (cyclic AMP-dependent transcription factor ATF-3), *JUN* (transcription factor AP-1), and *FOS* (proto-oncogene c-Fos) were upregulated more than 8-fold. In contrast, *NLRP3* (NACHT, LRP, and PYD domain-containing protein 3), *SGK1* (serine/threonine-protein kinase [PK] Sgk1), *EDN1* (endothelin-1), and *TLR4* (Toll-like receptor 4) were downregulated more than 2-fold.

10.1128/mSphere.00659-20.6 TABLE S2 Common upregulated immune genes in WT-infected and *ΔrtxA1* mutant-infected dTHP-1 cells. Download Table S2, PDF file, 0.1 MB. Copyright © 2020 Kim et al. 2020 Kim et al. This content is distributed under the terms of the Creative Commons Attribution 4.0 International license.

In HT-29 cells infected with *ΔrtxA1* V. vulnificus, 18 genes on the list were significantly upregulated compared with the mock control (Fig. 3B). The top four upregulated genes were *CCL20* (encoding C-C motif chemokine 20), *CXCL1* (growth-regulated alpha, or GRO-α), *CXCL2* (C-X-C motif chemokine 2), and *FOS*. All were upregulated more than 4-fold.

To identify MARTX toxin-regulated genes among the immune-related DEGs in HT-29 cells, up- and downregulated genes in either WT- or *ΔrtxA1* mutant-infected HT-29 cells were compared (Fig. 3C). As expected, most of the genes were regulated similarly in both WT- and *ΔrtxA1* mutant-infected HT-29 cells. However, *KLF2* (encoding Krueppel-like factor 2), *RHOB* (Rho-related GTP-binding protein RhoB), *JUND* (the transcription factor jun-D), *RORC* (RAR-related orphan receptor C), *KLF6* (Krueppel-like factor 6), *NUAK2* (NUAK family SNF1-like kinase 2), *ICAM1* (intercellular adhesion molecule 1), *NR4A2* (nuclear receptor subfamily 4 group A member 2), and *KLF4* (Krueppel-like factor 4) were specifically upregulated by the MARTX toxin-producing V. vulnificus (Fig. 3C). In contrast, *NLRP3*, *SGK1*, *EDN1*, *TLR4*, *RGCC* (regulator of cell cycle), and *CXCR4* (C-X-C chemokine receptor type 4) were specifically downregulated by the WT V. vulnificus strain (Fig. 3C). Among these genes, the expression of *JUND*, *KLF6*, *NLRP3*, *EDN1*, and *TLR4* was further examined by reverse transcription-quantitative PCR (RT-qPCR). As expected, all the genes were specifically upregulated (*JUND* and *KLF6*) or downregulated (*NLRP3*, *EDN1*, and *TLR4*) in WT-infected but not in *ΔrtxA1* mutant-infected HT-29 cells (Fig. S3A), further supporting the RNA sequencing results. Notably, there were no genes in HT-29 cells that were specifically up- or downregulated by infection with the *ΔrtxA1* strain (Fig. 3C).

10.1128/mSphere.00659-20.3 FIG S3 Validation of expression changes of immune-related genes in V. vulnificus-infected host cells and siderophore biosynthetic genes in V. vulnificus. (A and B) Expression of the indicated genes in V. vulnificus*-*infected HT-29 cells (A) or dTHP-1 cells (B) was determined by RT-qPCR and normalized to the levels in mock-infected control cells. (C) Secretion of the indicated cytokines by mock-treated, WT V. vulnificus-infected, and *ΔrtxA1* V. vulnificus-infected dTHP-1 cells. (D and E) Expression of siderophore biosynthetic genes in V. vulnificus not exposed (D) or exposed (E) to dTHP-1 cells. Expression of the indicated genes in WT or *ΔrtxA1* V. vulnificus was determined by RT-qPCR and normalized to the levels in WT V. vulnificus. Error bars represent the standard deviations (SD) of results from at least three biological replicates. Statistical significance was determined by the Student *t* test (****, *P < *0.0001; ***, *P < *0.0005; **, *P < *0.005; *, *P < *0.05; ns, not significant). Download FIG S3, PDF file, 0.3 MB. Copyright © 2020 Kim et al. 2020 Kim et al. This content is distributed under the terms of the Creative Commons Attribution 4.0 International license.

### Immune-related DEGs in dTHP-1 cells.

Among the genes on the focused gene list, 74 and 13 genes were significantly up- and downregulated, respectively, in dTHP-1 cells upon WT V. vulnificus infection compared with the mock control (Fig. 3D). Remarkably, *CSF2* (encoding granulocyte-macrophage colony-stimulating factor), *IL-6* (interleukin-6), *IL*-*12B* (interleukin-12 subunit beta), *CSF3* (granulocyte colony-stimulating factor), and *TNF* (tumor necrosis factor) were extremely highly expressed upon infection with WT V. vulnificus (log_2_ FC > 8). In contrast, *ZKSCAN3* (zinc finger protein with Krueppel-associated box [KRAB] and SCAN domains 3), *FADD* (FAS-associated death domain protein), *TLR3* (Toll-like receptor 3), and *NLRC4* (Nod-like receptor [NLR] family caspase recruitment domain [CARD]-containing protein 4) were the four most highly downregulated genes, with at least a 2.9-fold decrease.

Totals of 62 genes and 12 genes were significantly upregulated and downregulated, respectively, in dTHP-1 cells upon *ΔrtxA1* V. vulnificus infection compared with the mock control (Fig. 3E). Notably, *CSF2*, *TNF*, and *IL-6* were the most highly upregulated genes, as in the case of WT V. vulnificus infection, indicating that these genes respond to the V. vulnificus cells independently of the MARTX toxin. In contrast, *TLR3*, *ZKSCAN3*, and *TSC22D3* (encoding TSC22 domain family protein 3, also known as glucocorticoid-induced leucine zipper, or GILZ) were downregulated more than 2-fold in the *ΔrtxA1* V. vulnificus-infected dTHP-1 cells.

As in the results seen with infection of HT-29 cells, most of the DEGs were regulated by V. vulnificus itself, irrespective of the production of MARTX toxin (Fig. 3F). Upregulated immune genes common to WT-infected and *ΔrtxA1* mutant-infected dTHP-1 cells are listed in Table S2. All the genes for which expression increased more than 5-fold upon infection with WT cells were included on the common list of upregulated genes. Similarly, the top six genes that were downregulated upon infection with WT cells were also significantly downregulated upon infection with *ΔrtxA1* cells. These results suggest that the dTHP-1 cells responded mainly to factors expressed or produced by V. vulnificus other than the MARTX toxin. Nonetheless, there were a significant number of immune-related genes specifically regulated in WT strain-infected dTHP-1 cells, as described below.

Among the WT infection-specific upregulated genes in dTHP-1 cells (Fig. 3F), *TLR8* (Toll-like receptor 8), *CXCR4* (C-X-C chemokine receptor type 4), *TLR9* (Toll-like receptor 9), *STAT4* (signal transducer and activator of transcription 4), *RASD1* (Ras-related dexamethasone-induced 1), and *IKBKB* (inhibitor of nuclear factor [NF]-ĸB kinase [IKK] subunit beta, also known as IKK-β) were upregulated more than 2-fold. An additional seven genes, including *AMIGO2* (adhesion molecule with Ig-like domain 2), were also upregulated only in the WT-infected dTHP-1 cells, but to a lesser extent (Fig. 3F). In contrast, *IRAK3* (interleukin-1 receptor-associated kinase 3), *NOS2* (nitric oxide synthase), *IFNGR1* (interferon gamma receptor 1), *ZNF554* (zinc finger protein 554), and *PYCARD* (apoptosis-associated speck-like protein containing a CARD, also known as ASC) were downregulated approximately 1.6-fold only in the WT-infected dTHP-1 cells (Fig. 3F). Among these genes, the expression of *TLR8*, *TLR9*, *IFNGR1*, and *PYCARD* was further validated by RT-qPCR. Although WT infection-specific regulation was observed only for *TLR8* and *IFNGR1*, all four genes were expressed at higher levels (*TLR8* and *TLR9*) or at lower levels (*IFNGR1* and *PYCARD*) in the WT-infected dTHP1 cells than in the mock-treated or *ΔrtxA1* mutant-infected dTHP-1 cells (Fig. S3B). To examine the consequences of these gene expression changes, secretion of VGEF-A, TNF-α, and IL-6 from the dTHP-1 cells was examined by enzyme-linked immunosorbent assay (ELISA). Although statistical significance was not observed for the VGEF-A results, secretion of the three cytokines was more highly induced by infection with WT V. vulnificus than by infection with the *ΔrtxA1* mutant (Fig. S3C). These results suggest that the MARTX toxin robustly induces proinflammatory responses in immune cells.

There were also *ΔrtxA1* infection-specific DEGs in dTHP-1 cells. *FOS* was upregulated, while *AIM2* (encoding interferon-inducible protein AIM2), *ZNF665* (zinc finger protein 665), *TLR4* (Toll-like receptor 4), and *TLR1* (Toll-like receptor 1) were downregulated only in *ΔrtxA1* mutant-infected dTHP-1 cells (Fig. 3F). These genes might also have represented MARTX toxin-specific DEGs, if MARTX toxin-mediated dampening of these changes occurred only in cells infected with the WT strain.

### Genes showing distinct expression patterns in different cells upon V. vulnificus infection.

Among the immune-related DEGs, some genes showed completely different expression patterns in HT-29 cells and dTHP-1 cells (Fig. 3G). For example, *CXCR4* was downregulated in HT-29 cells but upregulated in dTHP-1 cells upon WT V. vulnificus infection. In addition, regardless of the strain used, *TSC22D3* was upregulated in HT-29 cells but downregulated in dTHP-1 cells. Notably, CXCR4 plays pivotal roles in inflammation (42), while GILZ, the protein encoded by *TSC22D3*, has a role in anti-inflammatory responses (43, 44). These results suggest that immune responses are dampened in HT-29 cells but enhanced in dTHP-1 cells upon V. vulnificus infection.

Similarly, *KLF4* was upregulated in HT-29 cells upon WT V. vulnificus infection but was downregulated in dTHP-1 cells upon infection with both WT and *ΔrtxA1* V. vulnificus. *NLRP3*, *EDN1*, and *SGK1* were downregulated in HT-29 cells upon WT V. vulnificus infection but upregulated in dTHP-1 cells upon both WT and *ΔrtxA1* V. vulnificus infection (Fig. 3G). These results also suggest that the inflammatory responses of the host cells against V. vulnificus differ. Although further studies are needed, this might be due to differences in the levels of expression of various receptors or signaling proteins, such as Toll-like receptors or their adaptors, between epithelial and immune cells (45, 46).

### DEGs in V. vulnificus during host cell infection.

**FIG 4:**
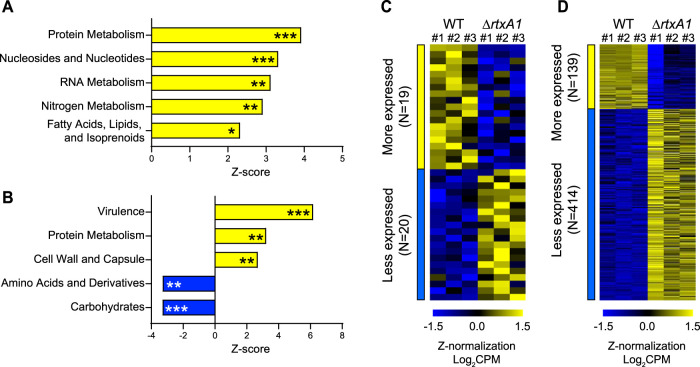
DEGs in the V. vulnificus strains during host cell infection. (A and B) Enriched KEGG pathways in WT V. vulnificus compared with *ΔrtxA1* V. vulnificus during infection of HT-29 cells (A) or dTHP-1 cells (B). *, *P < *0.05; **, *P < *0.01; ***, *P < *0.001. (C and D) DEGs that were expressed at higher levels or at lower levels in WT V. vulnificus than in *ΔrtxA1* V. vulnificus during infection of HT-29 cells (C) or dTHP-1 cells (D). CPM, counts per million mapped reads.

To examine MARTX toxin-dependent gene expression changes in V. vulnificus infecting host cells, the transcriptomes of the WT and *ΔrtxA1* V. vulnificus strains were directly compared during infection of either HT-29 or dTHP-1 cells (Fig. 4). First, significant biological changes were identified via a parametric analysis of gene set enrichment (PAGE) using custom gene sets defined from Kyoto Encyclopedia of Genes and Genomes (KEGG) pathways of V. vulnificus (47, 48). Upon infection of HT-29, the WT V. vulnificus strain expressed significantly higher levels of genes related to “protein metabolism,” “nucleosides and nucleotides,” “RNA metabolism,” “nitrogen metabolism,” and “fatty acids, lipids, and isoprenoids” than the *ΔrtxA1* strain (*P < *0.05) (Fig. 4A). Intriguingly, somewhat distinctive sets of genes were enriched significantly when V. vulnificus infected dTHP-1 cells (Fig. 4B). As in the case of HT-29 cell infection, genes involved in protein metabolism were significantly more highly expressed in the WT strain than in the *ΔrtxA1* strain. However, genes related to “carbohydrates” and “amino acids and derivatives” were expressed at significantly lower levels in the WT strain than in the *ΔrtxA1* strain.

Importantly, “virulence”-related genes, which were not significantly enriched upon infection of HT-29 cells, were strongly enriched in WT V. vulnificus, but not in the *ΔrtxA1* strain, during infection of dTHP-1 cells (Fig. 4B). Of note, this difference was not due to the expression of the MARTX toxin itself in the WT strain because *rtxA1* (locus tag VVMO6_03947) was omitted from the “virulence” gene set in the PAGE. Therefore, it seems that MARTX toxin-mediated changes in dTHP-1 cells specifically enhanced the expression of other virulence-related genes in WT V. vulnificus.

Next, we generated a list of DEGs in V. vulnificus by applying statistical cutoffs. In the case of HT-29 cell infection, slightly less stringent cutoff values (|FC| ≥ 1.5 and *P* value ≤ 0.05) were applied to increase the number of DEGs. As shown in Fig. 4C (see also Table S3), 19 and 20 genes were expressed at significantly higher and lower levels, respectively, in WT V. vulnificus than in the *ΔrtxA1* mutant. The genes expressed at higher levels in the WT strain included those encoding diguanylate cyclase (VVMO6_01132), nitrite reductase large subunit (VVMO6_03847), nitrite reductase small subunit (VVMO6_03846), and chemotaxis protein CheD (VVMO6_04552), while the genes that were expressed at higher levels in the *ΔrtxA1* strain included those encoding pyruvate dehydrogenase E1 component subunit alpha (VVMO6_03938), MFS transporter (VVMO6_04388), and S-formylglutathione hydrolase (VVMO6_02379).

10.1128/mSphere.00659-20.7 TABLE S3 DEGs in V. vulnificus (WT strain versus *ΔrtxA1* mutant) during HT-29 or dTHP-1 cell infection. Download Table S3, PDF file, 0.1 MB. Copyright © 2020 Kim et al. 2020 Kim et al. This content is distributed under the terms of the Creative Commons Attribution 4.0 International license.

In the case of dTHP-1 cell infection, statistical cutoff values of |FC| of ≥1.5 and FDR of ≤0.05 were applied. A total of 553 genes were identified as DEGs, where 139 and 414 genes were expressed at significantly higher and lower levels, respectively, in WT V. vulnificus than in the *ΔrtxA1* strain (Fig. 4D; see also Table S3). The genes expressed at higher levels in the WT strain included those encoding 3-deoxy-7-phosphoheptulonate synthase (VVMO6_04200), enterobactin synthase subunit E (VVMO6_04203), manganese transporter 11 TMS (VVMO6_01834), superoxide dismutase (VVMO6_00202), and microcin C ABC transporter ATP-binding protein (VVMO6_04162). Notably, a number of pilus-related genes, namely, the MshA pilin gene (VVMO6_03886) and the Tad1 pilin biogenesis locus genes (VVMO6_01213, VVMO6_01204, VVMO6_01205, and VVMO6_01211), were significantly (at least 2.4-fold) more highly expressed in the WT V. vulnificus strain than in the *ΔrtxA1* mutant during infection of dTHP-1 cells (Table S3). This result confirms the importance of these adhesive factors during infection (49–51). Moreover, it suggests that expression of the *tad1* locus is induced not simply by *in vivo* infection (49) but by some signal generated during MARTX toxin-mediated disruption of host cells.

In contrast, genes encoding ribosome hibernation protein YfiA (VVMO6_02482), universal stress protein A (VVMO6_00091), beta-galactosidase subunit alpha (VVMO6_00660), and d-ribose pyranase (VVMO6_03510) were expressed at significantly lower levels in WT V. vulnificus than in the *ΔrtxA1* strain (Table S3). Since these proteins are related to the stationary-phase, stress, and/or starvation conditions (52–54), it seems that the WT strain was subjected to less cellular stress than the *ΔrtxA1* strain during infection of dTHP-1 cells. This suggests that the MARTX toxin-mediated lysis of dTHP-1 host cells generated sufficient nutrients for WT V. vulnificus but that, in the absence of this cell lysis, fewer nutrients were available to the *ΔrtxA1* V. vulnificus. Notably, *vvhBA*, which encodes cytolysin secretion protein and cytolysin (VVMO6_03881 and VVMO6_03880), was expressed at lower levels in WT V. vulnificus than in the *ΔrtxA1* mutant (Table S3), suggesting that this exotoxin, in contrast to the MARTX toxin, may have a limited role in host cell lysis and subsequent nutrient generation. Since the WT pathogens experienced harsher iron-limiting conditions (see below), this result also suggests that such *vvhBA* expression might be largely driven by the carbon source shortage rather than by environmental cues such as iron limitation during infection (55–58).

### Significant overexpression of siderophore biosynthetic genes in dTHP-1 cell-infecting WT V. vulnificus.

To gain a more comprehensive understanding of the physiological changes occurring in V. vulnificus during dTHP-1 cell infection, the gene expression profiles were analyzed using the V. vulnificus KEGG pathway database (48), and significantly changed metabolic pathways were identified. Highly enriched pathways in WT V. vulnificus included “biosynthesis of siderophore group nonribosomal peptides,” “ribosome,” and “bacterial secretion systems.” Meanwhile, suppressed pathways in WT V. vulnificus included “valine, leucine, and isoleucine degradation,” “arginine biosynthesis,” “glycan degradation,” and “geraniol degradation” (Table S4).

10.1128/mSphere.00659-20.8 TABLE S4 Differentially regulated pathways in V. vulnificus (WT strain versus *ΔrtxA1* mutant) during dTHP-1 cell infection. Download Table S4, PDF file, 0.1 MB. Copyright © 2020 Kim et al. 2020 Kim et al. This content is distributed under the terms of the Creative Commons Attribution 4.0 International license.

**FIG 5:**
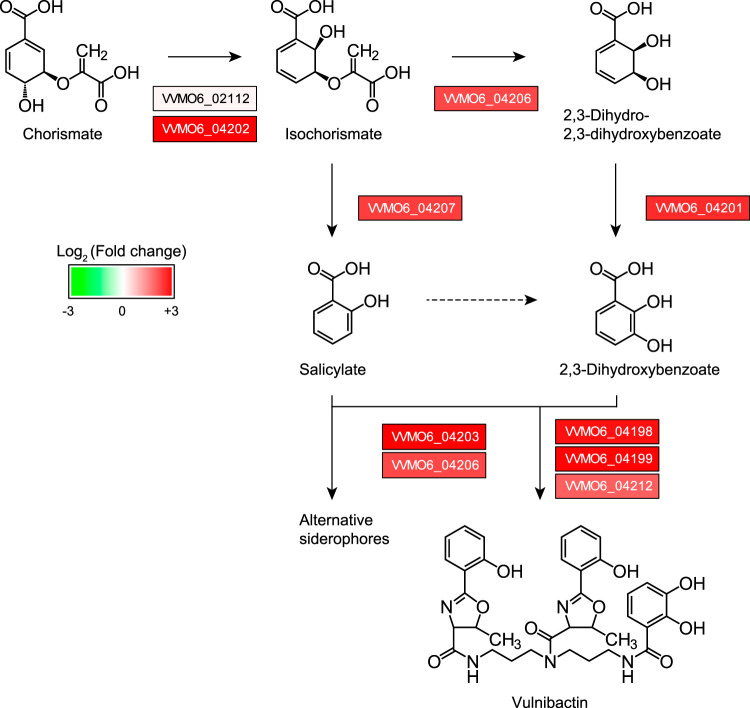
Vulnibactin biosynthesis genes are significantly more highly expressed in WT V. vulnificus than in *ΔrtxA1* V. vulnificus during dTHP-1 cell infection. Genes belonging to the vulnibactin biosynthesis pathway were identified from the KEGG pathway database and previous studies (60, 79).

Among these pathways, the siderophore biosynthetic pathway showed the highest Z-score in the analysis (Z-score of 13.8; Table S4). Indeed, not only siderophore vulnibactin biosynthesis genes (VVMO6_04197 to VVMO6_04212) but also aerobactin/enterobactin biosynthesis, binding, and transport genes (VVMO6_04403, VVMO6_04404, VVMO6_04408, and VVMO6_03836 to VVMO6_03841) were significantly more highly expressed in the WT V. vulnificus strain than in the *ΔrtxA1* strain during dTHP-1 cell infection (Fig. 5; see also Table S3). In addition, genes previously reported to be important for siderophore formation, and thus to be essential for V. vulnificus pathogenesis in mice (59, 60), such as the genes encoding isochorismatase (VVMO6_04206), isochorismate-pyruvate lyase (VVMO6_04207), and 2, 3-dihydroxybenzoate-2, 3-dehydrogenase (VVMO6_04201), were also more highly expressed in WT V. vulnificus than in the *ΔrtxA1* strain (5.5-, 5.9-, and 6.7-fold, respectively) (Fig. 5; see also Table S3). To validate these results, the expression of select vulnibactin biosynthetic pathway genes was examined by RT-qPCR. In this experiment, WT and *ΔrtxA1* V. vulnificus bacteria that had not been exposed to host cells were included as controls to determine whether the observed gene expression changes would occur only upon cell infection. As shown in Fig. S3D and E, all of the examined genes were much more highly expressed in WT V. vulnificus than in the *ΔrtxA1* mutant, but only upon dTHP-1 cell infection. Since these gene clusters are known to be upregulated under iron-limited conditions, this result suggests that the WT strain experienced more iron limitation during dTHP-1 cell infection than the *ΔrtxA1* strain.

Many genes related to bacterial secretion systems were also highlighted in the KEGG pathway analysis. In particular, the *gsp* genes (VVMO6_02862 to VVMO6_02872), which encode components of type II secretion system proteins, were significantly more highly expressed (about 1.5-fold) in the WT V. vulnificus strain than in the *ΔrtxA1* strain (Table S5). Furthermore, the *tolC* gene (VVMO6_02608), which encodes the outer membrane protein for the type I secretion system, and many *sec* and *tat* genes (VVMO6_00227, VVMO6_02445, VVMO6_02446, VVMO6_02568, VVMO6_02900, and VVMO6_02901), which encode Sec (secretion) and Tat (twin-arginine translocation) translocase system proteins, were also expressed at slightly but significantly higher levels in WT V. vulnificus (Table S5). It should be noted that the genes encoding a type I secretion system for the MARTX toxin (*rtxBDE* [VVMO6_03950 to VVMO6_03952]; 61) were not expressed more highly in WT V. vulnificus than in the *ΔrtxA1* strain. This indicates that general secretion systems, not the MARTX toxin-specific secretion system, are expressed at higher levels in the WT strain than in the *ΔrtxA1* strain during infection. This might allow the secretion of other exoenzymes that benefit V. vulnificus during MARTX toxin-mediated host cell lysis.

10.1128/mSphere.00659-20.9 TABLE S5 Differentially regulated secretion system genes in V. vulnificus (WT strain versus *ΔrtxA1* mutant) during dTHP-1 cell infection. Download Table S5, PDF file, 0.1 MB. Copyright © 2020 Kim et al. 2020 Kim et al. This content is distributed under the terms of the Creative Commons Attribution 4.0 International license.

### Differentially expressed iron homeostasis genes in dTHP-1 cells.

The higher expression of siderophore biosynthetic genes in WT V. vulnificus than in the *ΔrtxA1* strain upon dTHP-1 cell infection suggests that less iron was available to WT V. vulnificus than to the *ΔrtxA1* mutant during infection. Therefore, we hypothesized that iron- or heme-sequestering proteins might be produced by host cells in response to WT V. vulnificus infection. However, genes encoding iron/heme-sequestering proteins such as *LTF* (lactotransferrin), *HPX* (hemopexin), *HP* (haptoglobin), *TF* (transferrin), and *NGAL* (siderocalin) were not expressed in either mock-treated or WT-infected dTHP-1 cells under our experimental conditions (see deposited next-generation sequencing [NGS] data, Materials and Methods section) (62, 63). Thus, it seems that iron/heme-sequestering genes are not overexpressed by host cells during infection. Nonetheless, the possibility cannot be ruled out that stored sequestering proteins are released upon MARTX toxin attack, resulting in the relatively iron-limiting conditions experienced by WT V. vulnificus.

We further examined the expression of the host genes related to iron homeostasis. Although not all of these changes were statistically significant, some genes showed changes in expression during infection with WT V. vulnificus but not the *ΔrtxA1* mutant (Fig. S4A). Hepcidin, which is encoded by the *HAMP* gene, is an iron-regulatory hormone that governs iron absorption and distribution across tissues in mammals. Its presence leads to decreased release of iron from enterocytes and macrophages into the plasma by degradation of ferroportin, an iron exporter (62–64). As shown in Fig. S4A, *HAMP* expression was higher during infection with WT V. vulnificus than during infection with *ΔrtxA1* V. vulnificus. Therefore, the higher production of hepcidin might decrease the extracellular iron level during infection, which would then induce expression of siderophore biosynthetic genes by WT V. vulnificus (Fig. 5; see also Fig. S3D and E) (Tables S4 and S5).

10.1128/mSphere.00659-20.4 FIG S4 (A) Genes related to iron homeostasis in humans were differentially regulated in dTHP-1 cells during V. vulnificus infection. An asterisk (*) represents a statistically significant change in gene expression (|fold change| level of ≥1.5 and false-discovery rate [FDR] of <0.05). (B and C) Survival of host cells and pathogens at 6 h.p.i. (B) To estimate host cell survival, lactate dehydrogenase (LDH) activity in culture supernatants was measured, and percent cytotoxicity was calculated using the LDH activity from cells lysed with 5% Triton X-100 (100% lysis). (C) CFUs per well of either WT or *ΔrtxA1* V. vulnificus measured at 6 h.p.i. Error bars represent the SD of results from at least three biological replicates. Download FIG S4, PDF file, 0.2 MB. Copyright © 2020 Kim et al. 2020 Kim et al. This content is distributed under the terms of the Creative Commons Attribution 4.0 International license.

Similarly to the *HAMP* gene, *SLC11A1*, which encodes natural resistance-associated macrophage protein 1 (NRAMP1), was also more highly expressed in WT V. vulnificus-infected dTHP-1 cells (Fig. S4A). Since this protein transports irons from phagosomes to the cytoplasm (63), engulfed pathogens would experience relative iron limitation if this protein is overexpressed. Other genes that showed differences in expression between WT-infected and *ΔrtxA1* V. vulnificus-infected dTHP-1 cells were *FTH1* and *FTL*. These genes encode the heavy and light chains of ferritin, respectively, which stores iron in cells in a nontoxic, readily available form (63). Notably, these genes were expressed at lower levels in WT V. vulnificus-infected dTHP-1 cells (Fig. S4A). Reduced production of ferritin may be one mechanism through which host cells minimize the amount of readily available iron that can be hijacked by the pathogen. Taken together, these results suggest that host cells recalibrate their expression of iron homeostasis genes to limit the availability of iron that can be utilized by the invading extracellular pathogen, V. vulnificus.

## DISCUSSION

In this study, we explored MARTX toxin-specific gene expression changes in both host cells and the infecting species V. vulnificus. Although there were some genes that showed differential expression in the two types of host cells that we tested (HT-29 and dTHP-1 cells; Fig. 3G), gene sets related to immune responses were commonly regulated (Fig. 2B and E). Nonetheless, we were able to identify MARTX toxin-specific gene sets enriched only in the WT-infected cells. Notably, differential expression of those gene sets could be attributed to the biochemical function of the MARTX toxin effector domains. For instance, enrichment of genes related to negative regulation of ERK1/2 in HT-29 cells might be mediated by the RID, and genes involved in cell cycle regulation in dTHP-1 cells might be regulated by the DUF1 domain (Fig. 2C and F) (27, 39).

In a detailed analysis of 202 immune-related genes, we found that both host cell types upregulated some inflammatory genes upon V. vulnificus infection, regardless of the MARTX toxin; in HT-29 cells, these included the proinflammatory transcription factors *JUN*, *FOS*, and *ATF3* (Fig. 3C), while in dTHP-1 cells, these included proinflammatory cytokines/chemokines, including *CSF2*, *IL-6*, and *IL12B* (Fig. 3F; see also Table S2 in the supplemental material). These results were not surprising because MARTX toxin-deficient mutants can still produce pathogen-associated molecular patterns (PAMPs) such as those associated with lipopolysaccharide.

Nevertheless, MARTX toxin-specific regulation of immune-related genes was still apparent. Under conditions of infection with WT V. vulnificus, HT-29 cells upregulated *KLF2*, *KLF4*, and *KLF6* and downregulated *NLRP3*, *TLR4*, and *CXCR4* (Fig. 3C), suggesting that the MARTX toxin interrupts inflammatory responses, NF-κB signaling, and mitogen-activated protein kinase (MAPK) signaling in infected cells (65). Since gut epithelial cells such as HT-29 cells function not only as a barrier but also as a sensor of invading pathogens by releasing cytokines/chemokines that promote immune cell recruitment (66), these changes may help V. vulnificus to evade immune-related clearance at the infection site, the gut.

In the case of dTHP-1 cells, however, somewhat different responses occurred upon WT V. vulnificus infection. Although *PYCARD* was downregulated, which is consistent with inhibition of inflammation, upregulation of *TLR8*, *TLR9*, *CXCR4*, *STAT4*, *IKBKB*, and *IRF7* and downregulation of *IRAK3* were also observed (Fig. 3F), suggesting that the MARTX toxin enhances inflammation and NF-κB signaling (67–69). This may correlate with the uncontrolled septic immune responses that usually occur in cases of V. vulnificus systemic infection (12, 31). Notably, the contrasting expression patterns of the anti-inflammatory gene *TSC22D3* and the inflammatory gene *EDN1* further support such opposite outcomes of V. vulnificus infection in HT-29 and dTHP-1 cells (Fig. 3G) (43, 44, 70).

V. vulnificus also exhibited MARTX toxin-dependent gene expression changes during infection (Fig. 4). Most importantly, various siderophore biosynthesis, binding, and transport genes were significantly more highly expressed in the WT strain than in the MARTX toxin-deficient mutant strain during dTHP-1 cell infection (Fig. 5; see also Table S3). Although we did not observe overexpression of iron-sequestering protein genes in the host, the relative upregulation of hepcidin and NRAMP1 and the relative downregulation of ferritin (see Fig. S4A in the supplemental material) support the notion that dTHP-1 cells limit iron levels to inhibit the invading pathogens.

It should be noted that the final numbers of host and V. vulnificus cells at 6 h.p.i. were different. Regardless of the host cell type, many more cells were lysed by WT V. vulnificus than by the *ΔrtxA1* strain (31.3% versus 12.4% lysis for HT-29 cells; 67.6% versus 21.8% lysis for dTHP-1 cells), probably due to the potent cytotoxicity of the MARTX toxin (Fig. S4B). Since cellular components released from lysed cells can be used as nutrients by the infecting pathogens, the difference in cell lysis might affect the gene expression profiles of V. vulnificus. Indeed, expression levels of genes related to protein metabolism were considerably increased in WT V. vulnificus (Fig. 4A and B), while stress/starvation-related genes such as *yfiA* were expressed at significantly lower levels (Table S3). Consistent with this, higher numbers of WT V. vulnificus cells than of *ΔrtxA1* cells were present at 6 h.p.i. (5.5 × 10^7^ versus 2.6 × 10^7^ CFU/well for HT-29 infection; 5.1 × 10^6^ versus 3.4 × 10^5^ CFU/well for dTHP-1 infection; Fig. S4C). The MARTX toxin also has a role in protecting bacteria from phagocytosis, which may further explain the lower number of *ΔrtxA1* cells at 6 h after dTHP-1 cell infection (71, 72). The increased phagocytosis of *ΔrtxA1* V. vulnificus compared with the WT cells might also affect the V. vulnificus transcriptome profiles. In this context, the significant increase in expression of genes related to arginine biosynthesis in the *ΔrtxA1* strain during dTHP-1 infection is of interest (Table S4) because in macrophages, arginine is consumed to produce nitric oxide to inhibit invading pathogens (73).

**FIG 6:**
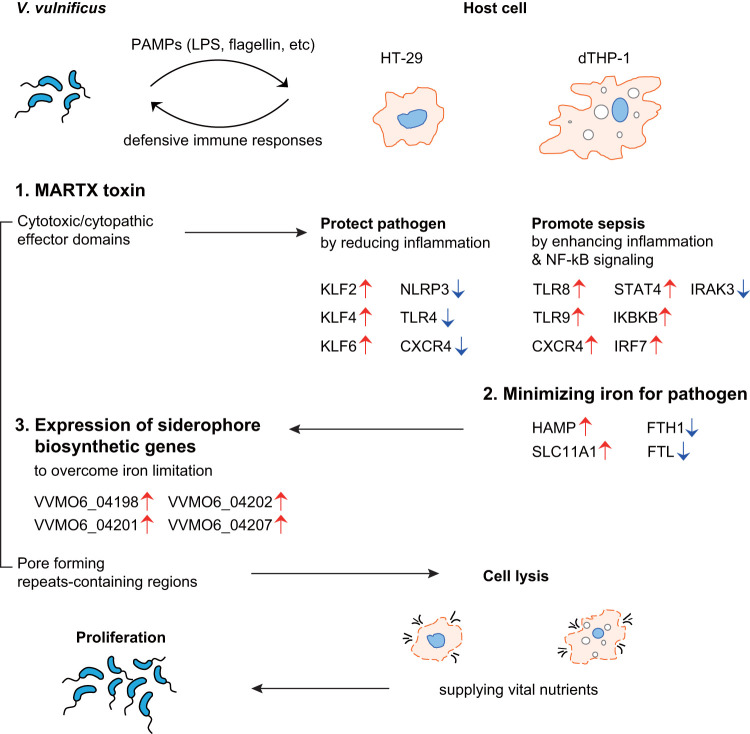
Proposed model for host-V. vulnificus interactions during infection. While the PAMP molecules of the pathogen trigger general immune responses in host cells, the MARTX toxin dysregulates immune-related genes in epithelial or immune cells to reduce inflammation or to enhance inflammation and NF-κB signaling, respectively. In response to this, host immune cells recalibrate the expression of iron homeostasis genes to minimize the iron available to the invading pathogen. Through the expression of siderophore biosynthetic genes, V. vulnificus overcomes this iron limitation. Host cells are eventually lysed by the pore-forming activity of the MARTX toxin, and the pathogen utilizes nutrients released by the lysed host cells to proliferate further. LPS, lipopolysaccharide.

In conclusion, during infection, the host cells and the invading V. vulnificus cells differentially regulate their levels of gene expression in response to each other (Fig. 6; see also Table S6A). After sensing PAMPs expressed by the invading pathogens, host cells reprogram their gene expression for defensive immune responses. In the meantime, V. vulnificus attacks host cells by secreting its primary virulence factor, the MARTX toxin. Due to the biochemical functions of MARTX effector domains, host signaling is subverted, resulting in different outcomes depending on the host cell type. In the case of gut epithelial cells, MARTX toxin downregulates inflammatory responses to protect V. vulnificus from clearance from the gut. In the case of immune cells, MARTX toxin upregulates proinflammatory responses that could result in uncontrolled sepsis. Nonetheless, the MARTX toxin eventually lyses the host cells via its pore-forming activity, which supplies V. vulnificus with vital nutrients. In the meantime, the toxin-affected host cells recalibrate iron homeostasis genes to minimize the amount of iron available to invading pathogens. Despite this host counterattack, V. vulnificus can overcome the relative limitation of iron by expressing siderophore biosynthetic and utilization genes, allowing the pathogen to survive and the infection to continue.

10.1128/mSphere.00659-20.10 TABLE S6 Differentially regulated host and V. vulnificus genes during infection and the oligonucleotides used in RT-qPCR. Download Table S6, PDF file, 0.1 MB. Copyright © 2020 Kim et al. 2020 Kim et al. This content is distributed under the terms of the Creative Commons Attribution 4.0 International license.

## MATERIALS AND METHODS

### Strain construction and culture conditions.

V. vulnificus MO6-24/O, a clinical isolate, was used as the parental WT strain for the construction of the MARTX toxin-deficient (*ΔrtxA1*) mutant. Briefly, a suicide plasmid (pMW0613) containing an internally deleted *rtxA1* gene fragment with the kanamycin resistance cassette *nptI* at the deleted region (*ΔrtxA1*::*nptI*) was transferred to the parental strain via conjugation, as described previously (15). The transconjugants that underwent first-round homologous recombination were selected and then challenged with 20% (wt/vol) sucrose to induce second-round homologous recombination. The constructed mutant strain, which lacked 9,190 bp of the 14,112 bp-long *rtxA1* gene but contained the 1.2-kb *nptI* cassette, was confirmed by PCR and sequencing. Since MARTX toxin has a secretion signal sequence and amino acid repeats essential for toxin secretion at the carboxyl-terminal region (29), the mutant cannot secrete the toxin even if a truncated version is produced. V. vulnificus strains were grown in Luria-Bertani medium supplemented with 2.0% (wt/vol) NaCl (LBS) at 30°C.

HT-29 and THP-1 cells were purchased from the American Type Culture Collection (ATCC) and cultured at 37°C with 5% CO_2_ in RPMI 1640 medium (Thermo Fisher Scientific, Waltham, MA) supplemented with 1% antibiotic/antimycotic (Thermo Fisher Scientific) and 10% fetal bovine serum (FBS; Thermo Fisher Scientific).

### Bacterial infection and RNA purification.

For HT-29 cells, 24 h before infection, 10^6^ cells in 2 ml of RPMI 1640 per well were seeded onto 6-well plates (SPL, Seoul, South Korea). On the following day, cells were washed twice with PBS, and then fresh medium without antibiotic/antimycotic or FBS was added. WT and *ΔrtxA1* V. vulnificus cells were cultured to the mid-exponential phase, as determined by an optical density at 600 nm (OD_600_) of approximately 0.5. The bacterial cells were washed, diluted with PBS, and added to the HT-29 cells at an MOI of 0.001.

For THP-1 cells, about 2 × 10^5^ cells were seeded in 75-T flasks (SPL) and then treated with 100 ng/ml of phorbol 12-myristate 13-acetate (PMA; Thermo Fisher Scientific) to induce differentiation. The residual PMA was washed out for 3 days, and 10^6^ dTHP-1 cells in 2 ml of RPMI 1640 per well were seeded onto 6-well plates (SPL). On the following day, cells were washed with PBS and fresh medium without antibiotic/antimycotic or FBS was added. Bacterial cells at the mid-exponential phase were prepared as described above and added to the dTHP-1 cells at an MOI of 0.0002. It should be noted that neither the WT strain nor *ΔrtxA1* V. vulnificus was eliminated at this MOI.

After addition of the bacterial cells, the plates were lightly centrifuged (300 × *g*, 3 min) to synchronize the cell infection and were then placed in a CO_2_ incubator. After 3 or 6 h, samples were treated with RNAlater reagent (Qiagen, Hilden, Germany) to stabilize the transcriptomes. Both host and V. vulnificus cells were harvested by scraping, and total RNA was isolated using an RNeasy minikit (Qiagen) according to the manufacturer’s instructions. For RNA sequencing, host (human) and bacterial (V. vulnificus) ribosomal RNAs (rRNAs) were depleted using an Illumina Ribo-Zero Plus rRNA depletion kit (Illumina, San Diego, CA) according to the manufacturer’s instructions.

### RNA sequencing.

RNA sequencing libraries were prepared using a TruSeq RNA sample prep kit (Illumina), and the sequencing was performed using an Illumina HiSeq 2000 platform to generate 100-bp paired-end reads. Bioinformatic analysis was conducted as described elsewhere (74), with slight modifications. Trim galore! (v.0.6.5; https://www.bioinformatics.babraham.ac.uk/projects/trim_galore/) was used to trim low-quality reads using the default parameters, and FastQC (v.0.11.9; https://www.bioinformatics.babraham.ac.uk/projects/fastqc/) was used to perform a quality check. Human and Vibrio vulnificus reference genomes were obtained from NCBI Genome (https://www.ncbi.nlm.nih.gov/genome/), and genome indexing was performed using STAR (v.2.5.1) (75). The sequenced reads were mapped to the human genome (hg19) and the Vibrio vulnificus MO6-24 genome (https://www.ncbi.nlm.nih.gov/assembly/GCA_000186585.1) using STAR. The sequencing depths were at least 10 and 1.8 million reads for human cells and V. vulnificus cells, respectively (see Table S1A in the supplemental material). In the cases of human rRNA and tRNA, annotation was performed using the University of California, Santa Cruz (UCSC), Table browser (76). In the case of V. vulnificus, rRNA and tRNA were identified using NCBI protein annotation (https://www.ncbi.nlm.nih.gov/genome/browse/#!/proteins/189/165751%7CVibrio%20vulnificus%20MO6-24~2FO/). Among the mapped reads, any that were cross-mapped to human and V. vulnificus genomes or that were mapped to rRNAs or tRNAs were removed from the fastq files (see Fig. S1 and Table S1A in the supplemental material). Then, the gene expression levels were quantified using the count module of STAR. The edgeR (v.3.12.1) package was used to select DEGs among the mock-treated, WT-infected, and *ΔrtxA1* mutant-infected samples (77). The upper quantile normalization method was used to calculate the library size, and the value representing counts per million mapped reads (CPM) for each gene was added to 1 and the result subjected to log_2_ transformation for further analysis.

### Gene set analysis of the human gene expression profile.

To functionally annotate the differently expressed genes among the mock-treated, WT-infected, and *ΔrtxA1* mutant-infected samples, gene ontology (GO) analyses were performed using DAVID (34). *P* values of less than 0.05 were considered to represent statistically significant enrichment in the annotation category.

### Gene set analysis of the Vibrio vulnificus gene expression profile.

As the functional gene sets of Vibrio vulnificus MO6-24 strain were defined only in the KEGG pathway, we created a custom gene set using the KEGG pathway DB. All the KEGG pathways associated with Vibrio vulnificus MO6-24 were collected using Pathview (v.1.23) (78), and detailed gene expression changes in the selected pathways were displayed using the “pathview” module of the same program. The gene set enrichment test was performed by PAGE (47) using the custom gene set, and the results were summarized by z-scores and *P* values.

### RT-qPCR, Western blotting, and ELISAs.

For RT-qPCR, cDNA was synthesized using an RT^2^ Easy first-strand kit (Qiagen), and real-time PCR amplification of the cDNA was performed with a qRT-PCR kit (BioAssay, South Korea) and specific primer pairs for each target gene (Table S6B). The relative expression levels of the target transcripts were calculated using the expression of the GAPDH (glyceraldehyde-3-phosphate dehydrogenase) gene (*GAPDH* [for host cell genes]) or *rrsH* (for V. vulnificus genes) as the internal reference for normalization.

For Western blot analysis, at 6 h.p.i., host cells were lysed with 2× Laemmli sample buffer containing protease inhibitors and phosphatase inhibitors, boiled, and separated on Bis-Tris gels. Then, the proteins were transferred onto polyvinylidene difluoride (PVDF) membranes and incubated with primary antibodies, secondary antibodies, and ECL Select chemiluminescent substrate (GE Healthcare Life Science, Chicago, IL). The antibodies included rabbit polyclonal anti-phospho-p44/42 MAPK (Erk1/2) (Cell Signaling Technology, Danvers, MA; catalog no. 9101) (1:1,000), rabbit monoclonal anti-p44/42 MAPK (Erk1/2) (Cell Signaling Technology; catalog no. 4695) (1:1,000), rabbit polyclonal anti-PLK1 (Cell Signaling Technology; catalog no. 4535) (1:1,000), horseradish peroxidase (HRP)-conjugated rabbit monoclonal anti-β-actin (Cell Signaling Technology; catalog no. 12620) (1:5,000), and an HRP-conjugated anti-rabbit IgG secondary antibody (Cell Signaling Technologies, catalog no. 7046S) (1:5,000).

For ELISAs, culture supernatants were harvested at 6 h.p.i., and the amounts of secreted cytokines were measured with a human TNF ELISA set (BD Bioscience, catalog no. 555212), a human IL-6 ELISA set (BD Bioscience, catalog no. 555220), and a human vascular endothelial growth factor receptor (VEGF) Quantikine ELISA kit (R&D Systems, catalog no. DVE00) according to the instructions of the manufacturers.

### Lactate dehydrogenase (LDH) release assay and bacterial cell enumeration.

Cell culture supernatants were harvested at 6 h.p.i. and analyzed for lactate dehydrogenase (LDH) activity using a CytoTox 96 nonradioactive cytotoxicity assay kit (Promega, Madison, WI) according to the manufacturer’s instructions. Percent cell lysis was calculated as follows: (*A*_490_ of sample/*A*_490_ of 100% lysis control) × 100. To enumerate total bacterial cells at 6 h.p.i., the host cells were lysed by addition of 0.1% Triton X-100, and the lysates were serially diluted and plated on LBS agar. After overnight incubation at 30°C, the number of CFU per well was calculated.

### Data availability.

NGS data have been deposited in the NCBI Gene Expression Omnibus under accession number GSE136540. The raw sequence tags were deposited in the NCBI Short Read Archive (SRA) under accession number SRP219588.
