# Supplementary figures and images for: MARTX Toxin-Stimulated Interplay between Human Cells and Vibrio vulnificus
Source: mSphere. 2020 Aug 12;5(4):e00659-20. doi: 10.1128/mSphere.00659-20 (PMC7426173; doi:10.1128/mSphere.00659-20)

**A**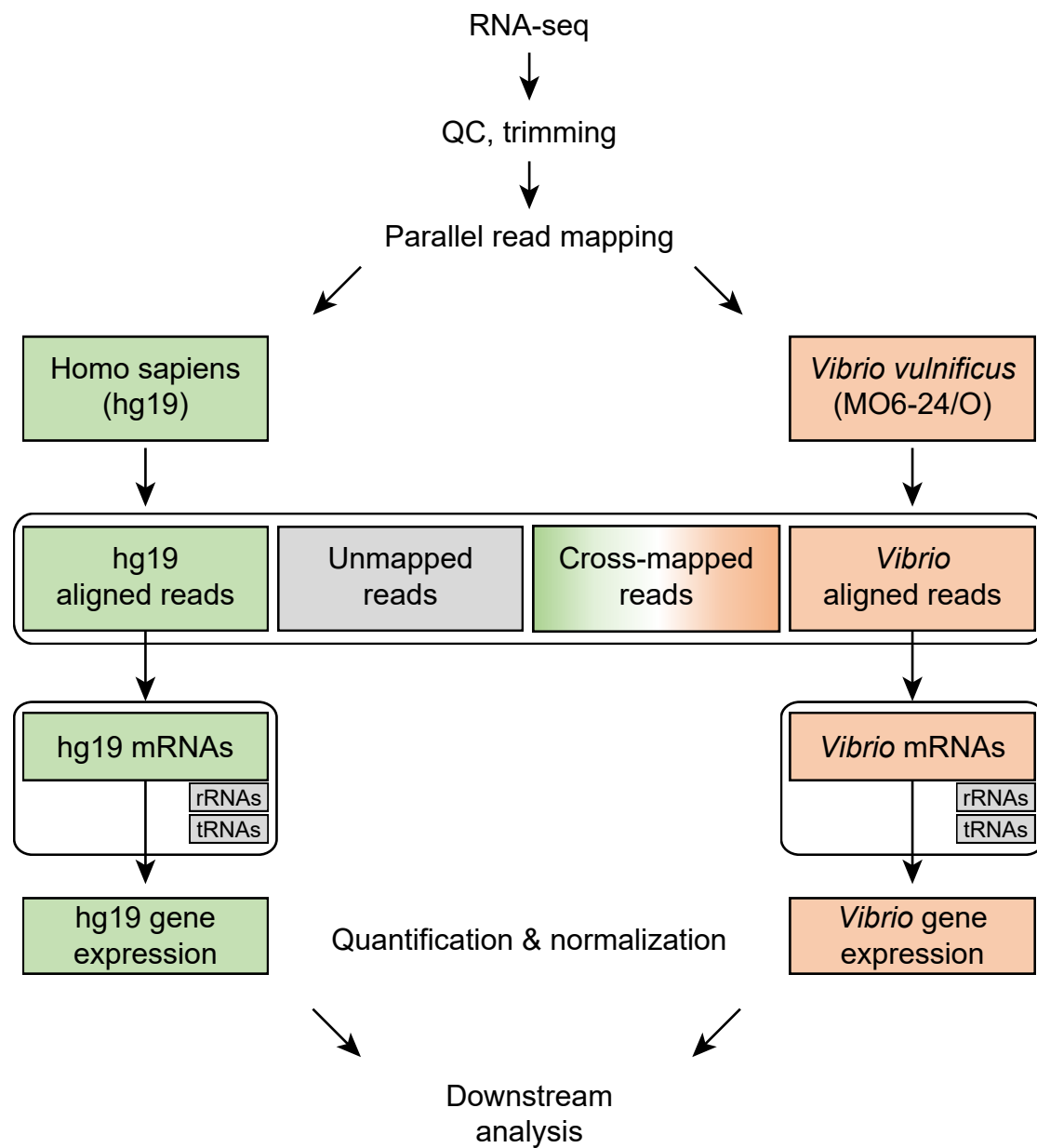**B**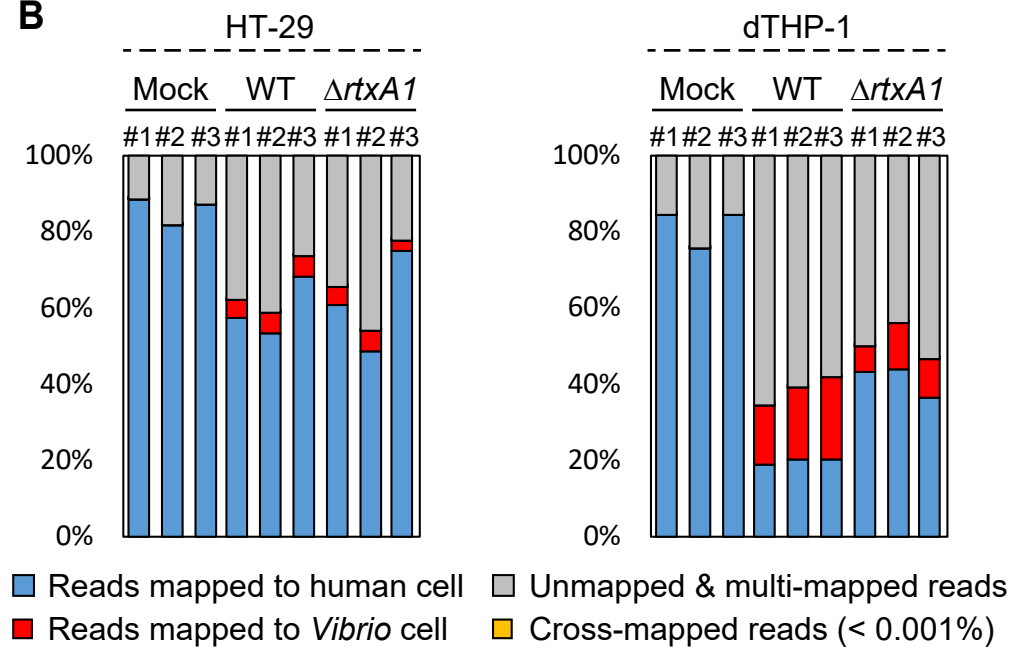**C**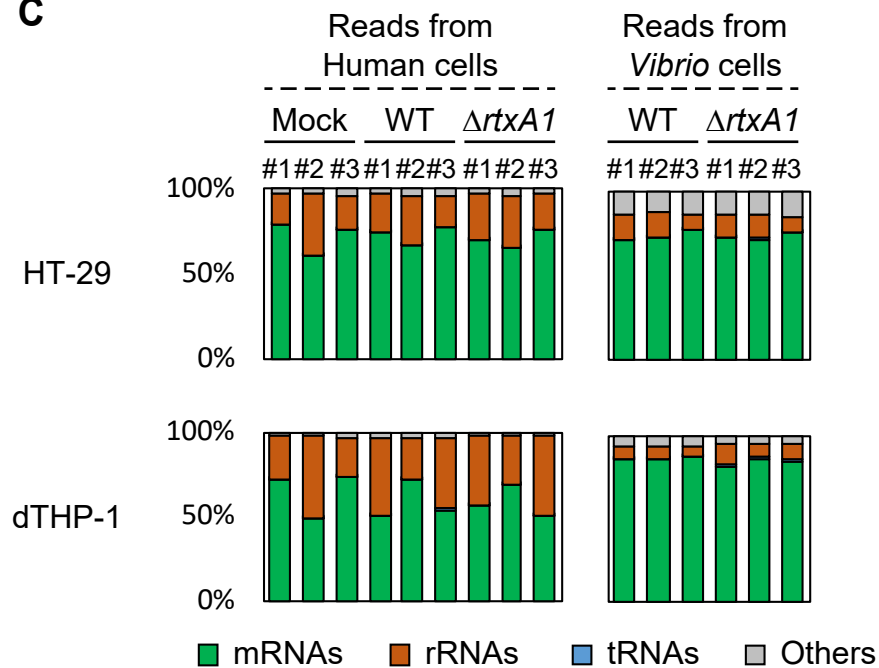

Supplement: FIG S1 [file mSphere.00659-20-sf001.pdf]

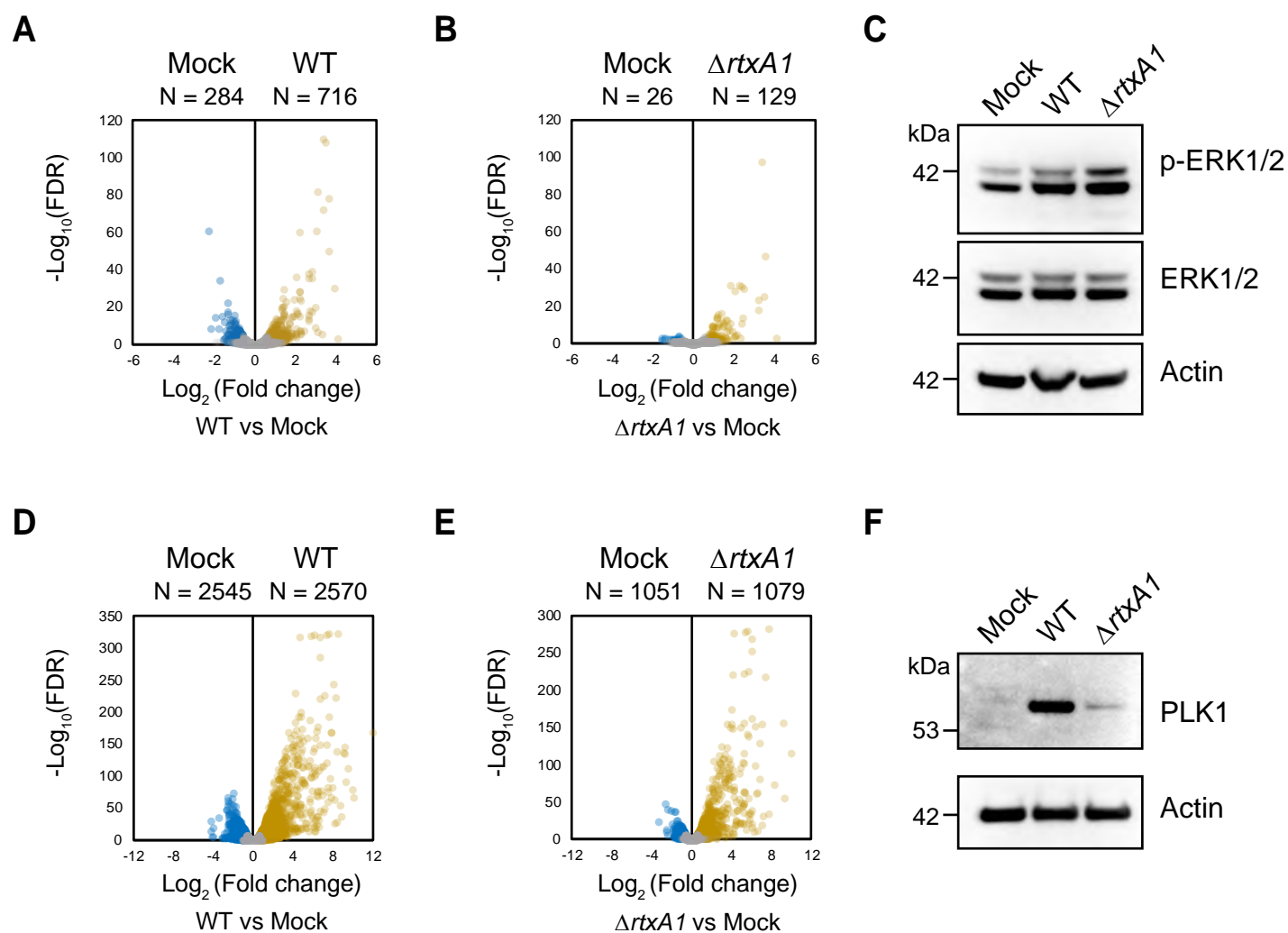

Figure S2. Kim *et al.*

Supplement: FIG S2 [file mSphere.00659-20-sf002.pdf]

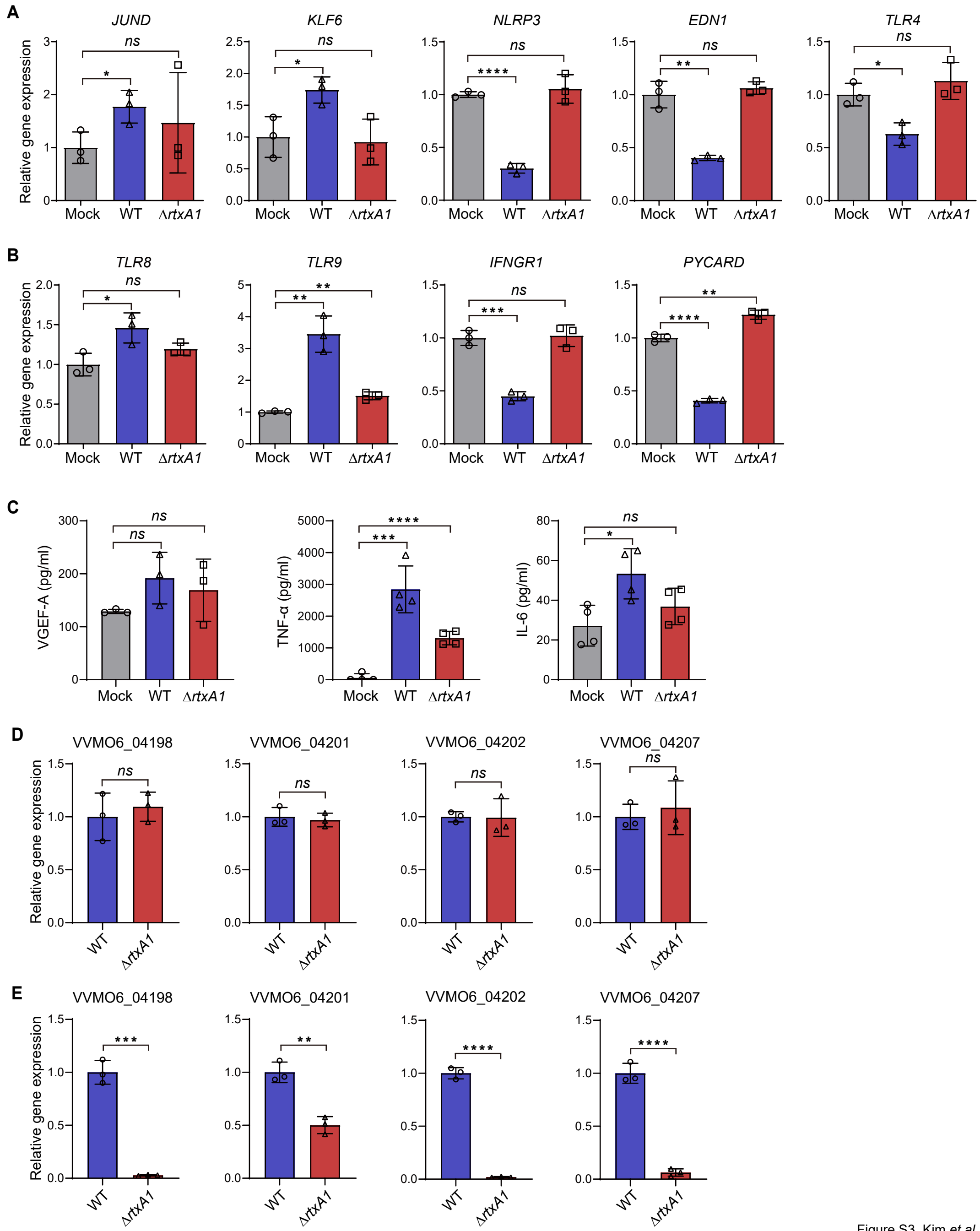

Figure S3. Kim et al.

Supplement: FIG S3 [file mSphere.00659-20-sf003.pdf]

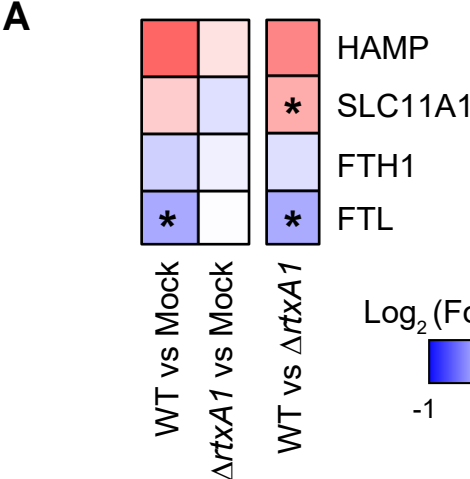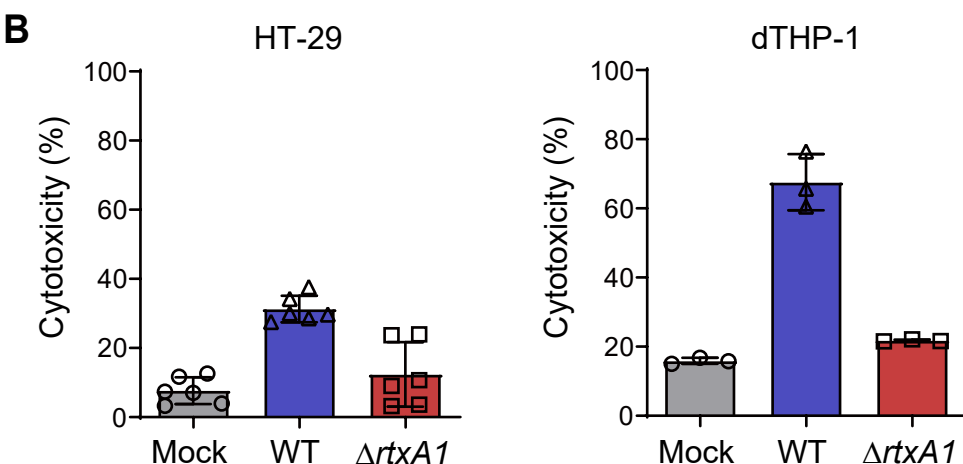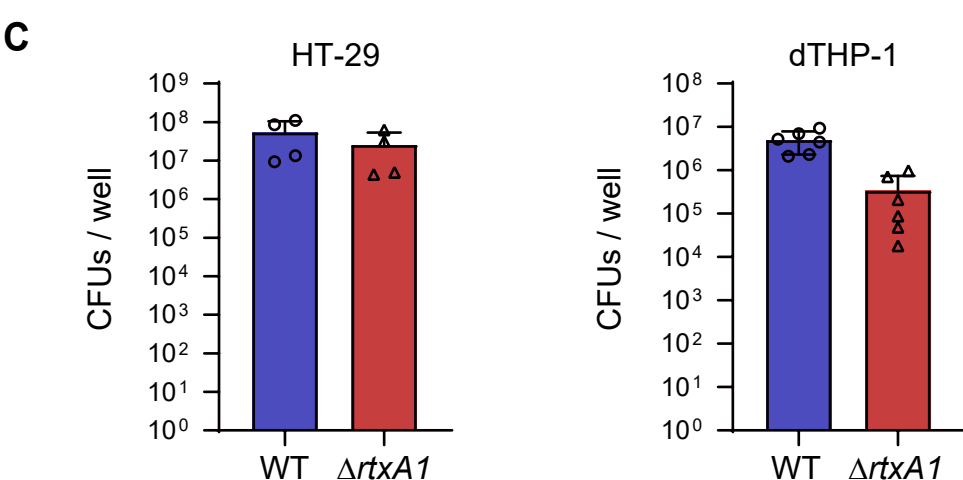

Figure S4. Kim *et al.*

Supplement: FIG S4 [file mSphere.00659-20-sf004.pdf]
