# Supplementary material for: MARTX Toxin-Stimulated Interplay between Human Cells and Vibrio vulnificus
Source: mSphere. 2020 Aug 12;5(4):e00659-20. doi: 10.1128/mSphere.00659-20 (PMC7426173; doi:10.1128/mSphere.00659-20)
Supplement: TABLE S1 [file mSphere.00659-20-st001.pdf]

Table S1. The number of reads for each sample and list of immune-related genes used in this study

(A) The number of reads for each sample

Experiment: Exposed to HT-29 cells

The reads mapped to human genome only

Sample* name		Protein-coding (mRNAs)	tRNAs	rRNAs	others (ncRNAs, snoRNAs, snRNAs, pseudo genes, and unknown)	Total
Mock 6h	#1	30,555,410	12,795	6,924,043	1,187,411	38,679,659
	#2	22,101,168	10,315	13,509,124	1,030,141	36,650,748
	#3	31,639,185	17,654	8,610,329	1,520,849	41,788,017
WT 6h	#1	20,685,727	4,828	6,303,164	828,091	27,821,810
	#2	16,492,451	5,425	7,305,904	881,731	24,685,511
	#3	27,646,586	9,660	6,264,311	1,439,529	35,360,086
ΔrtxA1 6h	#1	19,778,593	5,651	7,600,698	797,674	28,182,616
	#2	13,788,027	5,141	6,408,951	744,581	20,946,700
	#3	26,910,780	9,226	7,676,400	1,089,903	35,686,309

The reads mapped to *Vibrio* genome only

Sample name		mRNAs	tRNAs	rRNAs	others (hypothetical proteins)	Total
WT 6h	#1	2,185,406	7,841	459,704	380,295	3,033,246
	#2	2,175,973	8,214	417,555	359,669	2,961,411
	#3	2,897,136	13,372	321,965	498,621	3,731,094
ΔrtxA1 6h	#1	1,894,861	6,907	343,467	354,053	2,599,288
	#2	2,072,547	8,403	430,194	364,047	2,875,191
	#3	1,379,736	7,226	163,542	264,750	1,815,254

Experiment: Exposed to dTHP-1 cells

The reads mapped to human genome only

Sample name		Protein-coding (mRNAs)	tRNAs	rRNAs	others (ncRNAs, snoRNAs, snRNAs, pseudo genes, and unknown)	Total
Mock 6h	#1	32,679,424	14,087	11,150,396	1,218,627	45,062,534
	#2	22,680,569	12,107	22,112,762	999,725	45,805,163
	#3	34,171,279	18,665	11,316,503	1,401,646	46,908,093
WT 6h	#1	5,526,481	4,127	4,961,332	347,786	10,839,726
	#2	8,354,900	4,823	2,738,633	488,964	11,587,320
	#3	6,269,090	3,527	4,882,583	404,683	11,559,883
ΔrtxA1 6h	#1	15,467,262	13,433	11,043,702	710,208	27,234,605
	#2	17,727,665	10,666	7,494,839	696,452	25,929,622
	#3	10,845,368	12,401	10,289,574	566,058	21,713,401

The reads mapped to *Vibrio* genome only

Sample name		mRNAs	tRNAs	rRNAs	others (hypothetical proteins)	Total
	#1	9,615,626	60,998	934,333	701,303	11,312,260

WT 6h	#2	13,023,413	71,480	1,083,750	1,030,977	15,209,620
	#3	14,573,006	69,447	954,855	1,141,632	16,738,940
	#1	4,470,084	31,925	657,533	311,328	5,470,870
ΔrtxA1 6h	#2	7,599,369	43,303	663,353	531,025	8,837,050
	#3	6,165,981	40,462	639,036	428,993	7,274,472

* Only 6 h.p.i samples are shown since the number of mapped reads correspond to the *Vibrio* mRNAs was less than 1 million in 3 h.p.i samples. Also there are no significant differences among the mock control, WT-infected, and *ΔrtxA1*-infected groups in the overall human gene expression profiles of the 3 h.p.i.

(B) List of immune-related genes used in this study

Gene	Conding protein
AIM2	Interferon-inducible protein AIM2
AKT1	RAC-alpha serine/threonine-protein kinase
AMIGO2	Amphoterin-induced protein 2
ANK1	Ankyrin-1
APCS	Serum amyloid P-component
ATF3	Cyclic AMP-dependent transcription factor ATF-3
BAX	Apoptosis regulator BAX
BIRC3	Baculoviral IAP repeat-containing protein 3
BPI	Bactericidal permeability-increasing protein
C10ORF10	Protein DEPP1 (Decidual protein induced by progesterone)
C3	Complement C3
C4B	Complement C4-B
CAMP	Cathelicidin antimicrobial peptide (18 kDa cationic antimicrobial protein
CARD6	Caspase recruitment domain-containing protein 6
CARD9	Caspase recruitment domain-containing protein 9
CASP1	Caspase-1
CASP3	Caspase-3
CASP8	Caspase-8
CCL2	C-C motif chemokine 2
CCL20	C-C motif chemokine 20
CCL3	C-C motif chemokine 3
CCL4	C-C motif chemokine 4
CCL5	C-C motif chemokine 5
CCR4	C-C chemokine receptor type 4,
CCR5	C-C chemokine receptor type 5
CCR6	C-C chemokine receptor type 6
CCR8	C-C chemokine receptor type 8
CD14	Monocyte differentiation antigen CD14
CD4	T-cell surface glycoprotein CD4
CD40	Tumor necrosis factor receptor superfamily member 5
CD40LG	CD40 ligand, CD40-L (Tumor necrosis factor ligand superfamily member 5)
CD80	T-lymphocyte activation antigen CD80
CD82	CD82 antigen (C33 antigen)
CD86	T-lymphocyte activation antigen CD86 (Activation B7-2 antigen)
CD8A	T-cell surface glycoprotein CD8 alpha chain
CDKN2B	Cyclin-dependent kinase 4 inhibitor B
CHUK	Inhibitor of nuclear factor kappa-B kinase subunit alpha
CLEC1A	C-type lectin domain family 1 member A
CLEC4D	C-type lectin domain family 4 member D
CLEC4E	C-type lectin domain family 4 member E

CRP	C-reactive protein
CSF2	Granulocyte-macrophage colony-stimulating factor
CSF3	Granulocyte colony-stimulating factor, G-CSF
CTSG	Cathepsin G
CXCL1	Growth-regulated alpha protein (C-X-C motif chemokine 1)
CXCL10	C-X-C motif chemokine 10
CXCL2	C-X-C motif chemokine 2
CXCL3	C-X-C motif chemokine 3
CXCL6	C-X-C motif chemokine 6
CXCR3	C-X-C chemokine receptor type 3
CXCR4	C-X-C chemokine receptor type 4
DDX58	Probable ATP-dependent RNA helicase DDX58
DMBT1	Deleted in malignant brain tumors 1 protein
DUSP1	Dual specificity protein phosphatase 1
DUSP5	Dual specificity protein phosphatase 5
DUSP8	Dual specificity protein phosphatase 8
EDN1	Endothelin-1 (Preproendothelin-1, PPET1)
EGR1	Early growth response protein 1
EPB41	EPB41 protein, Protein 4.1
FADD	FAS-associated death domain protein
FASLG	Tumor necrosis factor ligand superfamily member 6
FOS	Proto-oncogene c-Fos (Cellular oncogene fos)
FOXP3	Forkhead box protein P3
GATA3	Trans-acting T-cell-specific transcription factor GATA-3
GEM	GTP-binding mitogen-induced T-cell protein
HLA-A	HLA class I histocompatibility antigen, A-1 alpha chain
HLA-E	HLA class I histocompatibility antigen, alpha chain E
HSP90AA1	Heat shock protein HSP 90-alpha
ICAM1	Intercellular adhesion molecule 1
IFNA1	Interferon alpha-1/13
IFNA2	Interferon alpha-2
IFNAR1	Interferon alpha/beta receptor 1
IFNB1	Interferon beta
IFNG	Interferon gamma
IFNGR1	Interferon gamma receptor 1
IKBKB	Inhibitor of nuclear factor kappa-B kinase subunit beta
IL10	Interleukin-10
IL11	Interleukin-11
IL12A	Interleukin-12 subunit alpha
IL12B	Interleukin-12 subunit beta
IL13	Interleukin-13
IL17A	Interleukin-17A
IL18	Interleukin-18
IL1A	Interleukin-1 alpha
IL1B	Interleukin-1 beta
IL1R1	Interleukin-1 receptor type 1
IL1R2	Interleukin-1 receptor type 2
IL1RN	Interleukin-1 receptor antagonist protein
IL2	Interleukin-2
IL23A	Interleukin-23 subunit alpha
IL3	Interleukin-3
IL4	Interleukin-4
IL5	Interleukin-5
IL6	Interleukin-6

IL7	Interleukin-7
IL8	Interleukin-8
IRAK1	Interleukin-1 receptor-associated kinase 1
IRAK3	Interleukin-1 receptor-associated kinase 3
IRF1	Interferon regulatory factor 1
IRF2	Interferon regulatory factor 2
IRF3	Interferon regulatory factor 3
IRF5	Interferon regulatory factor 5
IRF7	Interferon regulatory factor 7
ITGAM	Integrin alpha-M (CD11 antigen-like family member B)
JAK2	Tyrosine-protein kinase JAK2
JUN	Transcription factor AP-1
JUNB	Transcription factor jun-B
JUND	Transcription factor jun-D
KIAA0754	Uncharacterized protein KIAA0754
KLF2	Krueppel-like factor 2
KLF4	Krueppel-like factor 4
KLF6	Krueppel-like factor 6
KRAS	GTPase KRas
LBP	Lipopolysaccharide-binding protein
LCN2	Neutrophil gelatinase-associated lipocalin, NGAL
LTA	Lymphotoxin-alpha
LTF	Lactotransferrin
LURAP1L	Leucine rich adaptor protein 1-like
LY96	Lymphocyte antigen 96
LYZ	Lysozyme C
MAP2K1	Dual specificity mitogen-activated protein kinase kinase 1
MAP2K3	Dual specificity mitogen-activated protein kinase kinase 3
MAP2K4	Dual specificity mitogen-activated protein kinase kinase 4
MAP3K7	Mitogen-activated protein kinase kinase kinase 7
MAPK1	Mitogen-activated protein kinase 1
MAPK14	Mitogen-activated protein kinase 14
MAPK3	Mitogen-activated protein kinase 3
MAPK6	Mitogen-activated protein kinase 6
MAPK8	Mitogen-activated protein kinase 8
MBL2	Mannose-binding protein C
MEFV	Pyrin
MPO	Myeloperoxidase
MX1	Interferon-induced GTP-binding protein Mx1
MYD88	Myeloid differentiation primary response protein MyD88
NAIP	Baculoviral IAP repeat-containing protein 1
NFKB1	Nuclear factor NF-kappa-B p105 subunit
NFKBIA	NF-kappa-B inhibitor alpha
NLRC4	NLR family CARD domain-containing protein 4
NLRP1	NACHT, LRR and PYD domains-containing protein 1
NLRP3	NACHT, LRR and PYD domains-containing protein 3
NOD1	Nucleotide-binding oligomerization domain-containing protein 1
NOD2	Nucleotide-binding oligomerization domain-containing protein 2
NOS2	Nitric oxide synthase
NR1D1	Nuclear receptor subfamily 1 group D member 1
NR4A1	Nuclear receptor subfamily 4 group A member 1
NR4A2	Nuclear receptor subfamily 4 group A member 2
NUAK2	NUAK family SNF1-like kinase 2
PF4	Platelet factor 4

PIK3CA	Phosphatidylinositol 4,5-bisphosphate 3-kinase catalytic subunit alpha isoform
PRTN3	Myeloblastin
PSTPIP1	Proline-serine-threonine phosphatase-interacting protein 1
PTGS2	Prostaglandin G/H synthase 2
PYCARD	Apoptosis-associated speck-like protein containing a CARD
RAC1	Ras-related C3 botulinum toxin substrate 1
RASD1	Dexamethasone-induced Ras-related protein 1
RELA	Transcription factor p65
RGCC	Regulator of cell cycle RGCC
RGS2	Regulator of G-protein signaling 2
RHOB	Rho-related GTP-binding protein RhoB
RIPK1	Receptor-interacting serine/threonine-protein kinase 1
RIPK2	Receptor-interacting serine/threonine-protein kinase 2
RORC	Nuclear receptor ROR-gamma
SDC4	Syndecan-4
SELL	L-selectin
SGK1	Serine/threonine-protein kinase Sgk1
SLC11A1	Natural resistance-associated macrophage protein 1
SLPI	Antileukoproteinase, ALP
STAT1	Signal transducer and activator of transcription 1-alpha/beta
STAT3	Signal transducer and activator of transcription 3
STAT4	Signal transducer and activator of transcription 4
STAT6	Signal transducer and activator of transcription 6
SUGT1	Protein SGT1 homolog
TBX21	T-box transcription factor TBX21
TFRC	Transferrin receptor protein 1
THBD	Thrombomodulin
TICAM1	TIR domain-containing adapter molecule 1
TICAM2	TIR domain-containing adapter molecule 2
TIRAP	Toll/interleukin-1 receptor domain-containing adapter protein
TLR1	Toll-like receptor 1
TLR2	Toll-like receptor 2
TLR3	Toll-like receptor 3
TLR4	Toll-like receptor 4
TLR5	Toll-like receptor 5
TLR6	Toll-like receptor 6
TLR7	Toll-like receptor 7
TLR8	Toll-like receptor 8
TLR9	Toll-like receptor 9
TNF	Tumor necrosis factor
TNFRSF1A	Tumor necrosis factor receptor superfamily member 1A
TOLLIP	Toll-interacting protein
TRAF3	TNF receptor-associated factor 3
TRAF6	TNF receptor-associated factor 6
TRIM56	E3 ubiquitin-protein ligase TRIM56
TSC22D3	TSC22 domain family protein 3
TYK2	Non-receptor tyrosine-protein kinase TYK2
VEGFA	Vascular endothelial growth factor A
XIAP	E3 ubiquitin-protein ligase XIAP
ZBP1	Z-DNA-binding protein 1
ZKSCAN3	Zinc finger protein with KRAB and SCAN domains 3
ZNF554	Zinc finger protein 554
ZNF665	Zinc finger protein 665
ZNF681	Zinc finger protein 681
