# Supplementary material for: MARTX Toxin-Stimulated Interplay between Human Cells and Vibrio vulnificus
Source: mSphere. 2020 Aug 12;5(4):e00659-20. doi: 10.1128/mSphere.00659-20 (PMC7426173; doi:10.1128/mSphere.00659-20)
Supplement: TABLE S2 [file mSphere.00659-20-st002.pdf]

Table S2. Common up-regulated immune genes in WT- and *Δ rtxA1* -infected dTHP-1 cells

Gene	Expression (log_2 CPM)									Coding protein
	Mock_6h_1st	Mock_6h_2nd	Mock_6h_3rd	WT_6h_1st	WT_6h_2nd	WT_6h_3rd	ΔrtxA1_6h_1st	ΔrtxA1_6h_2nd	ΔrtxA1_6h_3rd	
**ATF3**	4.367050805	4.247927283	4.266502468	5.749522117	5.808968448	5.85695935	6.08014362	5.994140933	6.107088269	Cyclic AMP-dependent transcription factor ATF-3
**BIRC3**	2.491820098	2.308061787	2.808266941	8.328922862	8.585849469	8.858908857	6.486546257	7.652880797	7.484231856	Baculoviral IAP repeat-containing protein 3
**C3**	7.49242824	7.826161338	7.338986822	9.200122683	9.040406329	8.976976196	8.27493564	7.84382837	8.391203382	Complement C3
**CCL2**	2.353604674	2.509860845	2.34008947	3.377809133	3.398377112	3.263130272	3.555922605	3.738599796	3.637038515	C-C motif chemokine 2
**CCL20**	6.45154502	6.098031844	6.678104891	10.68759245	10.8460426	10.99090666	9.31709351	10.32173196	10.0604126	C-C motif chemokine 20
**CCL3**	5.647966016	5.892918686	6.050896461	12.22602885	12.0825636	12.22094273	11.04328438	11.21425537	11.86308561	C-C motif chemokine 3
**CCL4**	3.532940066	3.560714732	3.894555465	11.37626869	11.49254885	11.77387446	10.4455339	10.9532809	11.18559905	C-C motif chemokine 4
**CCL5**	7.67553543	7.687078709	7.700242358	10.22721471	9.999573399	9.917174935	9.341365945	9.352790846	9.685634615	C-C motif chemokine 5
**CD40**	3.873640555	4.155917914	4.000997491	7.99351135	7.900825028	7.984928064	6.389359209	6.371820498	6.897895917	Tumor necrosis factor receptor superfamily member 5
**CD80**	0.94077946	0.895976823	1.245922075	4.568989607	4.592535957	5.064160181	2.862361848	3.249831396	3.413242263	T-lymphocyte activation antigen CD80
**CD82**	5.95707953	6.202144883	5.740417315	7.630127177	7.278893827	7.433380821	6.568279286	6.288929153	6.894648279	CD82 antigen (C33 antigen)
**CLEC4E**	0.282580064	0.257797718	0.551747992	2.885292685	2.95044628	3.569552406	1.569346004	2.072222815	1.880293888	C-type lectin domain family 4 member E
**CSF2**	0	0	0.072808687	6.710394658	6.531792954	6.888596656	4.343981324	4.589041145	5.359090309	Granulocyte-macrophage colony-stimulating factor
**CSF3**	0.11255081	0.349266751	0.240214228	6.602946102	6.573167568	6.701069082	3.726465474	4.353480874	4.939004229	Granulocyte colony-stimulating factor, G-CSF
**CXCL1**	4.219866451	4.155917914	4.529977145	10.71542878	10.86253926	11.13111455	9.680983838	9.995592596	10.01439926	Growth-regulated alpha protein (C-X-C motif chemokine 1)
**CXCL10**	1.130947586	1.336904156	1.214086619	1.860201	3.201354573	3.311094766	1.738398446	1.920281284	2.427548279	C-X-C motif chemokine 10
**CXCL2**	3.196591534	3.246606147	3.454734213	10.42236445	10.47579828	10.580049	8.876106735	9.12559869	9.407399677	C-X-C motif chemokine 2
**CXCL3**	4.025118088	3.757208589	4.18878427	10.55621206	10.65945648	10.70114854	8.938370304	9.2277556	9.397491002	C-X-C motif chemokine 3
**CXCL6**	2.498740375	2.24990678	2.775922776	3.429044753	3.601089683	3.96392322	2.774336694	3.89964379	3.472529162	C-X-C motif chemokine 6
**DUSP1**	4.567950981	4.571307244	4.701941058	8.968121454	8.82376741	8.516178378	8.529723722	8.553877763	8.773887711	Dual specificity protein phosphatase 1
**DUSP5**	5.19667452	5.210978087	5.230517462	6.783576742	6.595245208	6.734432188	5.959879331	6.241624432	6.566253997	Dual specificity protein phosphatase 5
**DUSP8**	1.546100883	1.709098632	1.214086619	5.454205955	5.250792156	5.254036408	4.493246099	4.671672905	4.959871617	Dual specificity protein phosphatase 8
**EDN1**	1.961225224	1.743224414	1.904349902	5.472637548	5.815252349	5.884961116	4.875150968	5.48280797	5.365360347	Endothelin-1 (Preproendothelin-1, PPET1)
**EGR1**	2.951300902	3.160835812	2.916081171	5.283756837	5.370836931	4.476263763	6.50424509	6.404404078	6.614429138	Early growth response protein 1
**GEM**	6.19509155	6.120925035	6.271869087	9.301122527	9.148873146	9.014127277	8.817144309	9.02528757	9.213375487	GTP-binding mitogen-induced T-cell protein
**ICAM1**	7.742840697	7.923546864	7.775959745	12.66468938	12.50647534	12.55153323	11.1236075	11.33974362	11.68420254	Intercellular adhesion molecule 1
**IL10**	1.835670659	1.544678746	2.142841984	5.391008995	5.287413942	5.769537491	3.983609495	4.855729525	4.696992803	Interleukin-10
**IL12B**	0	0	0.0368636	3.526360531	4.037166636	4.597262681	0.895976823	1.177036156	1.051441844	Interleukin-12 subunit beta
**IL1A**	1.250566047	1.485426671	1.366671962	6.440781578	6.582407678	6.805223261	5.424137356	6.145307597	6.21864776	Interleukin-1 alpha
**IL1B**	9.0221797	8.976557655	9.291994728	13.024001	13.02992094	13.07452683	12.00811058	12.52295743	12.61999477	Interleukin-1 beta
**IL1RN**	7.391699893	7.252048083	7.372721546	9.191339327	9.224300852	9.33477226	7.907715262	8.511713622	8.491348655	Interleukin-1 receptor antagonist protein
**IL23A**	1.930846032	1.917722137	1.853594924	9.654561237	9.591541495	9.9915004	7.241694298	7.892814627	8.143313395	Interleukin-23 subunit alpha
**IL6**	0	0	0.0368636	4.068971222	4.06906891	4.567950981	1.377822014	2.209675968	2.628594969	Interleukin-6
**IL7**	0.038500843	0.209792352	0.072808687	3.661033785	3.685835173	3.99362086	2.111197058	2.193193635	2.52037636	Interleukin-7
**IRF1**	5.235513334	5.291020273	5.165421126	8.056979184	7.885976035	8.086608412	6.593301561	6.627815016	6.880490085	Interferon regulatory factor 1
**JUN**	6.907896231	6.957228004	6.683563947	8.542457774	8.414842819	8.280527292	8.598623326	8.307450911	8.356608335	Transcription factor AP-1
**JUNB**	6.596084635	6.699775878	6.666021758	8.795848214	8.609092252	8.456782699	8.633888505	9.003592567	8.994966991	Transcription factor jun-B
**JUND**	6.093613536	6.129820328	5.996375854	6.976737831	6.870348482	6.754085883	6.700396396	6.886110583	6.97269712	Transcription factor jun-D
**KLF2**	0.434666554	0.349266751	0.361423236	2.068422707	2.434298164	2.291199364	0.577175383	0.451884744	1.432042008	Krueppel-like factor 2
**KLF6**	6.649920216	6.582149925	6.433953482	7.778249576	7.749082283	7.62345656	7.794399479	7.694463852	7.67424691	Krueppel-like factor 6
**LTA**	0.345351308	0.39291463	0.332062251	2.192218197	2.223683386	1.910231924	1.234375503	1.210179599	1.693580059	Lymphotoxin-alpha
**MAP2K3**	3.576110509	4.002350077	3.420270952	5.212435441	5.009086338	5.007062172	4.775024125	4.531201031	5.048263322	Dual specificity mitogen-activated protein kinase kinase 3
**NFKB1**	5.916311457	6.01054611	5.963244873	9.3024111	9.286117331	9.498756653	8.1869583	8.388862057	8.616545424	Nuclear factor NF-kappa-B p105 subunit
**NFKBIA**	5.672654791	5.705496925	5.740417315	11.86548343	11.76668899	11.62616724	11.11062734	11.19940607	11.43906818	NF-kappa-B inhibitor alpha

**NLRP3**	7.06622249	7.024681747	7.088518986	10.42650683	10.36424299	10.27231855	9.537950035	9.800272061	10.03826401	NACHT, LRR and PYD domains-containing protein 3
**NR1D1**	0.85708998	1.040206198	0.601813427	2.068422707	1.439921337	1.910231924	1.539139381	0.88028994	1.732906094	Nuclear receptor subfamily 1 group D member 1
**NR4A1**	0.768245155	0.925999303	0.697015425	4.741042359	4.532779987	4.837284894	3.509939486	3.894555465	4.318264053	Nuclear receptor subfamily 4 group A member 1
**NR4A2**	1.532677121	1.171318102	1.39534384	2.770799673	3.294257043	2.841267721	1.790616466	2.090137883	2.014010245	Nuclear receptor subfamily 4 group A member 2
**PTGS2**	3.144592836	2.972292859	3.41677891	8.202113229	8.377642671	8.667211596	6.334352171	7.14046343	7.062260869	Prostaglandin G/H synthase 2
**RELA**	5.780226946	5.97688137	5.642907557	6.563365217	6.573167568	6.45377245	6.2760201	6.487858763	6.45453535	Transcription factor p65
**RGS2**	5.894831477	5.887207129	6.111554382	6.657670464	6.721054116	6.773475433	6.706355271	6.736315546	6.877202983	Regulator of G-protein signaling 2
**RIPK2**	4.478015725	4.518096963	4.29341066	7.241449287	7.121724888	7.325127473	5.952735583	6.210227909	6.438270092	Receptor-interacting serine/threonine-protein kinase 2
**SDC4**	5.80695765	5.744979806	6.053147473	7.921542761	7.988931973	8.149796247	6.831584359	7.146361771	7.149315686	Syndecan-4
**SGK1**	5.910484926	5.935971884	5.986987241	7.929348232	7.956607973	7.96726214	7.499221717	7.778347933	7.740264832	Serine/threonine-protein kinase Sgk1
**SLPI**	1.420538421	1.146031662	1.245922075	2.131647883	1.931423629	1.97121111	1.95979301	1.58340829	1.693580059	Antileukoproteinase, ALP
**TICAM1**	3.640102294	4.026799831	3.534234692	5.403871794	5.169739976	5.394235462	5.134521748	4.956829891	5.380917249	TIR domain-containing adapter molecule 1
**TLR2**	7.641156211	7.561511855	7.867698845	8.806785499	9.00511071	9.094269176	8.165181416	8.680298776	8.328803666	Toll-like receptor 2
**TLR7**	3.157769267	3.096290977	3.222453725	4.386622446	4.562967334	4.635440955	3.989206492	4.292891239	4.217891397	Toll-like receptor 7
**TNF**	3.334491977	3.382727172	3.48172408	11.76943756	11.70356966	11.60294763	10.68507063	11.02510234	11.11060211	Tumor necrosis factor
**TRAF3**	6.325364457	6.477071661	6.374441345	7.702188716	7.719235315	7.809234572	7.319842953	7.362623197	7.50425781	TNF receptor-associated factor 3
**TYK2**	7.361647424	7.584635525	7.206976377	8.924155595	8.71033965	8.721463941	8.114547204	7.9585939	8.234763171	Non-receptor tyrosine-protein kinase TYK2
