# Supplementary material for: MARTX Toxin-Stimulated Interplay between Human Cells and Vibrio vulnificus
Source: mSphere. 2020 Aug 12;5(4):e00659-20. doi: 10.1128/mSphere.00659-20 (PMC7426173; doi:10.1128/mSphere.00659-20)
Supplement: TABLE S3 [file mSphere.00659-20-st003.pdf]

Table S3. DEGs in *V. vulnificus* (WT vs. *Δ rtxA1*) during HT-29 or dTHP-1 cell infection

locus_tag	Expression (log_2 CPM)						protein_description
	WT_6h_1st	WT_6h_2nd	WT_6h_3rd	ΔrtxA1_6h_1st	ΔrtxA1_6h_2nd	ΔrtxA1_6h_3rd	
**Genes expressed more in the WT *V. vulnificus* than in the *ΔrtxA1 V. vulnificus* upon HT-29 cell infection**							
VVMO6_03947	13.3275962	13.22517622	12.81817875	10.24926743	10.26179569	9.968927925	peptidase C80
VVMO6_03557	2.575722736	3.259574042	2.410844876	1.675301212	1.959718078	1.684270553	hypothetical protein
VVMO6_RS13915	1.866408615	2.301917038	2.017173215	0.791844711	1.059145742	1.913501542	hypothetical protein
VVMO6_04552	2.575722736	2.301917038	1.823714984	1.499892777	1.289793038	1.684270553	chemotaxis protein CheD
VVMO6_03846	4.159448784	4.291566978	3.575510481	2.429675039	3.88037531	2.440303036	nitrite reductase small subunit
VVMO6_01546	4.132493406	4.555486676	3.952102344	3.117447547	3.77010336	3.128990355	peptidase M15
VVMO6_02954	3.534180012	4.032729271	3.802562356	3.176985629	3.039954104	2.580387175	3-ketoacyl-CoA thiolase
VVMO6_03847	6.409697213	6.37223632	6.066903048	4.651480178	6.243652264	4.784783672	nitrite reductase large subunit
VMO6_t00072	2.575722736	2.301917038	3.268391455	1.831676096	2.205494011	2.285139576	tRNA
VVMO6_00802	5.593996054	5.948455105	6.033728193	4.542520957	5.505623927	4.999679898	hypothetical protein
VVMO6_01132	5.973974113	5.761521577	6.366869327	5.326387854	4.812109703	5.619110265	diguanylate cyclase
VVMO6_01559	4.813726633	5.358466856	4.749917128	3.490315294	4.83054264	4.267810949	MULTISPECIES: hypothetical protein
VVMO6_00279	4.237418744	4.094394689	3.693498505	3.234163727	3.565228345	3.300872631	sulfurtransferase
VVMO6_01565	3.896538071	3.677325251	3.342511558	2.524405747	3.218524885	3.300872631	MULTISPECIES: hypothetical protein
VVMO6_02342	3.263075361	2.893896316	2.826964851	2.429675039	2.598663974	2.111254356	hypothetical protein
VVMO6_01552	5.358408402	5.579425576	4.76347166	4.519705629	4.570319882	4.784783672	MULTISPECIES: hypothetical protein
VVMO6_02453	7.065803255	6.919793205	7.24186884	6.671259032	5.895294297	6.604358559	malate synthase A
VVMO6_00393	4.358677785	4.124266343	4.606869997	3.58126706	3.844546403	3.891041585	ornithine carbamoyltransferase
VVMO6_02452	5.796458954	6.0057455	5.680614945	5.378040438	5.127462016	5.186677157	isocitrate lyase
**Genes expressed less in the WT *V. vulnificus* than in the *ΔrtxA1 V. vulnificus* upon HT-29 cell infection**							
VVMO6_04298	3.726705833	3.968309925	3.693498505	3.786336909	4.456246437	4.755523152	hypothetical protein
VVMO6_03486	3.3592164	3.026357682	3.19025613	3.89670281	3.520515407	3.836193971	hypothetical protein
VVMO6_03810	7.366905622	7.341417902	7.492366391	7.788668207	8.462871965	7.673232661	hypothetical protein
VVMO6_01879	4.287151916	4.833794837	4.928987141	5.2998511	5.309317071	5.352194074	short-chain dehydrogenase
VVMO6_04317	4.04846695	4.317657065	4.651292762	4.865648683	4.953372025	5.119361322	hypothetical protein
VVMO6_00896	8.15217436	8.254950915	8.362632516	8.793746801	9.242467028	8.540559576	antibiotic biosynthesis monooxygenase
VVMO6_02235	2.575722736	2.585743972	2.478155946	3.234163727	3.039954104	2.933824873	membrane protein
VVMO6_02535	4.989253168	5.213148234	4.803387516	5.545518167	5.859838557	5.409674326	hypothetical protein
VVMO6_03866	2.797850292	2.669168758	3.020015648	3.055346248	3.608597067	3.719816731	nitrite reductase large subunit
VVMO6_01880	5.717035742	6.0057455	5.855344688	6.574949086	6.336861261	6.803342404	transcriptional regulator
VVMO6_01876	6.055160573	5.722995588	6.251368851	6.904894687	6.284338513	6.97183146	cyclopropane-fatty-acyl-phospholipid synthase
VVMO6_01878	5.670946149	5.906094151	5.545921484	6.386041893	6.557624089	6.3344258	amine oxidase
VVMO6_03431	2.106950308	2.19337772	2.017173215	2.613297638	2.975156273	2.440303036	2-aminoethylphosphonate--pyruvate aminotransferase
VVMO6_04309	2.406029451	2.497197408	2.017173215	2.776165937	3.218524885	2.708065049	DNA polymerase III subunit epsilon
VVMO6_03661	2.106950308	2.076003389	2.187730352	2.990451063	2.836116343	2.285139576	hypothetical protein
VVMO6_01823	2.106950308	1.948227242	1.923684396	2.524405747	2.836116343	2.440303036	phage-shock protein
VVMO6_04167	2.575722736	1.948227242	1.335682982	2.328284213	2.761225515	3.128990355	Fe-S oxidoreductase
VVMO6_02379	4.554160305	4.032729271	4.464753199	5.440109468	5.616002261	4.497250947	S-formylglutathione hydrolase
VVMO6_04388	1.216062964	1.948227242	1.474015935	2.219225481	2.598663974	2.111254356	MFS transporter

VVMO6_03938	2.106950308	2.076003389	1.923684396	2.697028878	2.836116343	3.379698464	pyruvate dehydrogenase (acetyl-transferring) E1 component subunit alpha
**Genes expressed more in the WT *V. vulnificus* than in the *ΔrtxA1 V. vulnificus* upon dTHP-1 cell infection**							
VVMO6_04200	8.547575973	8.615460503	9.034115854	4.189214466	6.31070993	5.568421015	3-deoxy-7-phosphoheptulonate synthase
VVMO6_04202	7.710024607	7.728269811	8.185016746	3.090950061	5.611567171	4.935376673	isochorismate synthase
VVMO6_04203	7.699071788	7.555135815	8.031693885	2.961041034	5.523859103	4.954630201	2,3-dihydroxybenzoate-AMP ligase
VVMO6_04199	11.32216389	11.29719572	11.67357834	6.511702821	9.343339043	8.603870312	hypothetical protein
VVMO6_04198	9.932046386	9.878552322	10.16774713	5.420270507	8.051981315	7.275039015	peptide synthetase
VVMO6_01834	8.921377411	8.743071826	9.161459984	4.602612839	7.022973936	6.636969152	manganese transporter 11 TMS
VVMO6_00202	9.425708086	9.462206362	9.825883604	5.304510902	7.585402219	7.356912661	superoxide dismutase
VVMO6_04211	12.24862841	12.55164534	12.9298531	7.733760915	11.03862465	10.01065986	ligand-gated channel protein
VVMO6_04201	7.065350385	7.053727297	7.319359994	2.85530621	5.440771606	4.855685357	2,3-dihydro-2,3-dihydroxybenzoate dehydrogenase
VVMO6_04208	9.643326316	9.579955165	9.838453058	5.456881089	7.791264041	7.617674497	2,3-dihydroxybenzoate-AMP ligase
VVMO6_04205	9.625401751	9.576786185	9.69651471	5.533153799	8.005323239	7.419453512	NADPH-dependent ferric siderophore reductase
VVMO6_04197	6.599155311	6.67846971	6.964124165	3.266208147	5.085717127	4.59442199	4\'-phosphopantetheinyl transferase
VVMO6_04207	9.135082171	9.065231164	9.32734882	5.126571598	7.597728352	7.074036392	isochorismate-pyruvate lyase
VVMO6_03280	6.561233108	7.133882372	7.4208477	3.090950061	5.617632631	4.882741197	hypothetical protein
VVMO6_04161	9.18765429	9.174923329	9.569320215	4.888249083	7.746989593	7.354498709	hypothetical protein
VVMO6_04206	9.29531447	9.394993292	9.659573877	5.521678811	8.083547648	7.32521178	isochorismatase
VVMO6_04162	10.05032806	9.986041804	10.26875467	6.109611154	8.676574806	8.124993004	microcin C ABC transporter ATP-binding protein
VVMO6_04210	9.268108218	9.435303827	9.559964779	5.544538236	7.983178679	7.482715088	enterochelin ABC transporter substrate-binding protein
VVMO6_04209	7.28203554	7.556680794	7.769628077	3.181238756	6.255376971	5.617566988	acyl carrier protein
VVMO6_03768	11.78947019	12.09645395	12.34721728	7.986303512	10.78260462	10.18398139	TonB-dependent receptor
VVMO6_04408	8.835139482	8.86883098	9.034115854	5.462893536	7.367864104	7.207091752	aerobactin siderophore receptor IutA
VVMO6_03947	12.35504497	12.13464744	12.15029628	10.39179519	9.92549969	10.26734681	peptidase C80
VVMO6_00201	8.708182989	8.629475985	8.746498595	6.10192602	6.86112533	6.976940258	RNA methyltransferase
VVMO6_02034	7.838882026	7.828874851	8.163824725	5.407857573	6.223914861	6.22609551	hypothetical protein
VVMO6_01202	9.189036461	9.465225735	9.851292743	5.917213359	8.122991488	7.979059097	hypothetical protein
VVMO6_03613	5.369322542	5.836807108	6.453397139	3.027457278	4.655591122	4.21254573	hypothetical protein
VVMO6_04212	7.87253262	7.772374643	8.033030179	4.852055788	6.557836518	6.161280516	short-chain isoprenyl diphosphate synthase
VVMO6_01208	8.29461589	8.180760719	8.467677184	4.991680423	6.711776857	7.045886732	hypothetical protein
VVMO6_01203	10.50832786	10.61585195	11.01735699	6.97840471	9.450438726	9.221769867	hypothetical protein
VVMO6_03279	5.496820484	5.434647283	5.780204446	3.151765932	4.295897001	3.706921858	FAD-dependent oxidoreductase
VVMO6_01204	7.247074974	7.341722925	7.856348179	4.02010833	6.021498239	6.196749617	pilin
VVMO6_01205	7.653074792	7.654194361	7.87397886	4.327440873	6.03742196	6.608888473	hypothetical protein
VVMO6_03317	10.92420165	10.84633888	10.81473432	7.369396543	9.618457836	9.4209271	peptidase S9
VVMO6_00141	9.537351064	9.504446933	9.665617268	6.830465265	8.218374072	8.108613115	O-methyltransferase
VVMO6_01214	6.10663291	5.919185679	6.244332798	2.741204882	4.763470741	4.922396741	hypothetical protein
VVMO6_02416	5.336438046	5.679309009	6.08081029	3.936159912	4.177712204	4.090208046	hypothetical protein
VVMO6_03841	8.247192252	8.365163087	8.406357145	5.550196942	7.025257207	6.935549176	biopolymer transporter TonB
VVMO6_03321	9.81443216	9.771192691	9.676132667	6.33285098	8.607015205	8.371134404	hypothetical protein
VVMO6_03318	10.90638671	10.70330793	10.62468475	7.48469503	9.537192901	9.415147409	ligand-gated channel
VVMO6_04383	6.564076601	6.220180305	6.29087557	4.27372887	4.325843632	5.318902545	hypothetical protein
VVMO6_01211	7.947400746	7.971604103	8.293530424	4.940891469	6.823573564	6.859277084	pilus assembly protein TadB
VVMO6_01213	6.509067936	6.384622883	6.730420402	3.397568165	5.36707872	5.353161085	pilus assembly protein TadD

VVMO6_03840	8.799086876	8.581712515	8.651167723	5.98980062	7.324023948	7.479395488	flagellar motor protein MotA
VVMO6_01216	8.079462466	8.134611036	8.314287013	4.999974098	6.912601405	7.131562135	hypothetical protein
VVMO6_03278	3.515169722	3.810564209	4.133443575	2.104911596	2.668077947	2.535060281	membrane protein
VVMO6_01215	5.717198335	5.722161496	5.875649991	2.741204882	4.480283081	4.862497034	hypothetical protein
VVMO6_03839	7.113700124	6.947030191	7.165804174	4.870265932	5.731080976	5.90831929	hypothetical protein
VVMO6_01212	7.205794225	7.196665476	7.446646977	4.174634554	6.219933412	6.210162703	flp pilus assembly protein TadC
VVMO6_01209	8.323430622	8.379201499	8.738534647	5.527427714	7.495214846	7.349658655	pilus assembly protein CpaE
VVMO6_03836	8.386635209	8.259714064	8.484380077	6.066827126	7.254687205	7.142797066	ferrichrome ABC transporter substrate-binding protein
VVMO6_03258	8.464030291	8.534144464	9.089752642	7.230043631	7.019542239	7.499199471	glycine C-acetyltransferase
VVMO6_02984	4.315446878	4.045836574	4.489359294	2.278579436	3.37471714	2.921700285	sugar ABC transporter ATP-binding protein
VVMO6_03838	5.881225728	5.83341208	5.936895673	3.752014387	4.684771772	4.734677136	biopolymer transporter protein ExbD
VVMO6_01649	5.493841154	5.222631536	5.26778611	4.02010833	4.08444354	3.660775175	membrane protein
VVMO6_03144	7.695834482	7.540635659	7.784778828	5.256859561	6.610489301	6.503887995	ligand-gated channel protein
VVMO6_01650	5.622169094	5.322878601	5.247208329	4.327440873	3.927281951	3.780697056	multidrug ABC transporter permease
VVMO6_03837	7.648393394	7.377129892	7.472485544	5.149119837	6.35495037	6.538326746	cell envelope biogenesis protein TonB
VVMO6_03333	5.207799815	5.00752532	4.91168448	3.121678429	3.927281951	3.943736864	hypothetical protein
VVMO6_03259	9.092634311	8.98775004	9.421806357	7.720020284	7.565144886	8.09638558	L-threonine 3-dehydrogenase
VVMO6_01648	6.321837404	6.331463154	6.385973745	5.07254671	5.124570173	4.792888221	membrane protein
VVMO6_04385	5.520436189	5.313161604	5.193247145	2.994631316	4.432912804	4.264899761	two-component sensor histidine kinase
VVMO6_03886	5.558005872	5.177822707	5.178831966	4.203648506	3.805040677	4.006583192	MSHA biogenesis protein MshA, partial
VVMO6_03255	5.289102934	5.372846041	5.13220981	3.181238756	4.08444354	4.411444835	ATPase
VVMO6_03053	6.127928121	6.240829536	6.460366718	4.327440873	5.501072847	4.889426613	hypothetical protein
VVMO6_03334	6.506113817	6.475636743	6.383880599	4.624234473	5.568379557	5.194855672	hypothetical protein
VVMO6_00902	8.662480203	8.769737758	8.805746617	6.121062638	7.863227003	7.830646823	hypothetical protein
VVMO6_04384	5.271813962	4.92699124	4.923260949	3.471001475	3.897678883	4.018830488	DNA-binding response regulator
VVMO6_00285	8.459074189	8.858637988	8.866597402	6.482405642	7.68912471	7.882670734	bacterioferritin
VVMO6_01651	5.813539751	5.63903911	5.624038849	4.697455984	4.446606622	4.285320742	ABC transporter
VVMO6_03332	6.178903051	6.076800834	5.893415666	4.465601249	4.967213545	5.017013858	hypothetical protein
VVMO6_03146	3.491466939	3.451713693	3.61064903	1.502985106	2.904730749	2.465178616	(2Fe-2S)-binding protein
VVMO6_04319	8.846570347	8.652777526	8.473594429	6.993020712	7.62959214	7.680697296	acriflavin resistance protein
VVMO6_03335	7.997693813	7.734198602	7.713645549	6.154881604	6.700391322	6.917673653	ATPase AAA
VVMO6_RS08020	8.556170429	8.754111671	8.787256565	7.651916701	6.935847324	7.836712954	MULTISPECIES: hypothetical protein
VVMO6_03330	6.784898714	6.567392028	6.56383726	4.966509433	5.804702578	5.526147125	aerotolerance protein BatD
VVMO6_03145	6.980627799	6.932823233	7.034530341	4.767140076	6.070963646	6.228733955	AraC family transcriptional regulator
VVMO6_03063	6.644190529	6.58667714	6.444386491	4.940891469	5.653499437	5.547438904	hypothetical protein
VVMO6_03331	6.703420898	6.550949562	6.329452752	4.879285527	5.685611961	5.491412859	hypothetical protein
VVMO6_00995	5.928330276	6.284995031	6.074334958	4.246103874	5.392063607	5.151017663	membrane protein
VVMO6_03052	6.088972679	6.142747261	6.483032554	4.737686623	5.577743502	5.059130947	hypothetical protein
VVMO6_00275	9.458551881	9.601945236	9.299858906	7.559548482	8.759143707	8.531988832	hypothetical protein
VVMO6_03609	6.55409976	6.520661394	6.50245661	4.569560393	5.764266338	5.773872954	iron ABC transporter ATP-binding protein
VVMO6_04320	7.744901046	7.744176766	7.499803709	5.998105078	6.90642129	6.83524013	membrane protein
VVMO6_04403	6.731516246	6.546809497	6.65605106	4.676911992	5.825813927	6.12208187	(2Fe-2S)-binding protein
VVMO6_00510	9.171663411	9.262259065	9.117731534	7.808851902	8.508043137	8.256219204	peptidase M16
VVMO6_04526	8.156844189	8.044989017	8.169607693	7.159605979	7.274977127	7.105959009	hypothetical protein

VVMO6_01534	9.721658398	9.8758732	9.779067667	8.509994231	9.11639136	8.871614233	GlyGly-CTERM sorting domain-containing protein
VVMO6_04509	8.363154742	8.091206725	8.390285255	7.142850869	7.407788503	7.481609403	bifunctional proline dehydrogenase/L-glutamate gamma-semialdehyde dehydrogenase
VVMO6_02600	10.00500974	9.928333668	9.788988253	8.520121232	9.233089115	9.097722078	peptidase
VVMO6_02096	6.891749943	7.036852512	7.466069157	6.139948738	5.899852616	6.586435139	cysteine synthase
VVMO6_00509	5.520436189	5.731311186	5.606194415	4.429214093	4.874012004	4.828112408	membrane protein
VVMO6_01835	6.76526548	6.193946077	6.518763897	5.369964804	5.815296868	5.613534832	alpha-galactosidase
VVMO6_03172	5.250789939	4.920606032	4.996325133	4.052366072	4.003376519	4.475050914	uridine phosphorylase
VVMO6_00027	7.589603944	7.812155903	7.841934365	6.443407595	7.026397489	7.021748162	thiol:disulfide interchange protein
VVMO6_02983	3.479467895	3.379058457	3.673690976	2.85530621	2.884751013	2.391738929	dipeptide/oligopeptide/nickel ABC transporter ATP-binding protein
VVMO6_00108	4.406979793	3.967471343	4.143376284	3.090950061	3.223842232	3.676321989	DTW domain-containing protein
VVMO6_00469	9.040756967	8.912522116	8.830741573	7.693419109	8.154101064	8.239963663	anti-sigma E factor
VVMO6_00971	9.929292292	9.891975804	10.55156876	9.176463689	9.307907899	9.350599746	keto-acid formate acetyltransferase
VVMO6_00468	10.00736202	9.957573723	10.01171261	8.773336905	9.276145424	9.251265164	RNA polymerase sigma factor AlgU
VVMO6_00866	5.635747887	5.812872405	5.786556479	4.66652924	4.967213545	5.041231108	membrane protein
VVMO6_00854	6.76526548	6.72318239	6.511112835	5.964596523	5.775160634	5.737282016	aromatic hydrocarbon degradation protein
VVMO6_00056	9.715916787	9.589043128	9.438037251	8.592518829	8.772117336	8.837448802	transporter
VVMO6_00276	10.38581132	10.55583051	10.36381463	9.241300726	9.754556471	9.768337501	peptidyl-prolyl cis-trans isomerase
VVMO6_00685	8.012341998	8.14594551	8.335020079	7.271740325	7.360649301	7.424065352	hydrolase
VVMO6_03276	2.350156526	2.387300284	2.011221732	1.835115512	1.661260139	1.475798496	MBL fold metallo-hydrolase
VVMO6_00028	7.292360616	7.441541851	7.37205241	6.458531854	6.581302892	6.680020479	stress response kinase A
VVMO6_02868	8.119155519	8.041315125	7.951284685	7.075776951	7.200153684	7.455941922	type II secretion system protein GspG
VVMO6_01218	4.677664627	4.222339204	4.32016176	3.05955293	4.144489786	3.62916973	hypothetical protein
VVMO6_04500	2.569779237	2.802859233	2.677079153	2.104911596	1.918731908	2.232593149	arylsulfatase
VVMO6_01639	7.754268277	7.966963824	8.148494684	7.033982837	7.357934421	7.219083216	hypothetical protein
VVMO6_03195	7.033903176	7.120712601	7.562220804	6.316342168	6.721666022	6.468607006	hypothetical protein
VVMO6_03851	7.127221853	7.371873927	7.319907507	6.699099308	6.671528062	6.180487549	MaoC family dehydratase
VVMO6_04306	3.430448129	3.433890025	3.491285246	2.994631316	2.62054549	2.813375016	2-deoxy-D-gluconate 3-dehydrogenase
VVMO6_01217	5.218668039	5.485282057	5.674573499	4.083918297	5.098784748	4.869276701	FMN-dependent NADH-azoreductase
VVMO6_00057	8.235646136	8.157191629	8.05259809	7.21587256	7.511593379	7.493725578	MexH family multidrug efflux RND transporter periplasmic adaptor subunit
VVMO6_02226	7.953377494	7.77591621	8.27501428	7.303916259	7.239034391	7.314074068	molecular chaperone HtpG
VVMO6_04404	8.605309291	8.495733105	8.604742788	7.188920684	7.882875566	8.253630594	iron ABC transporter substrate-binding protein
VVMO6_02870	8.612194469	8.390507973	8.141077998	7.483216786	7.661501474	7.840167896	type II secretion system protein GspE
VVMO6_02871	9.255190243	9.183948677	9.018010238	8.22523071	8.551463437	8.491915872	type II secretion system protein GspD
VVMO6_00210	9.647521549	9.285893943	9.797760562	9.05186599	8.649818159	8.902099565	molecular chaperone GroEL
VVMO6_03735	5.762464263	6.585668535	6.262674892	5.745344771	5.427022025	5.437691287	hypothetical protein
VVMO6_02384	6.552668849	6.513254191	6.870804356	5.904015942	5.958377035	6.046332102	molecular chaperone DnaJ
VVMO6_01105	7.068357599	7.208507696	7.121895268	6.117255567	6.672985022	6.492955084	disulfide bond formation protein B
VVMO6_04350	7.262912151	7.446550466	7.605976787	6.565779796	6.876382546	6.847308667	DNA repair ATPase
VVMO6_00470	8.644121807	8.440178902	8.267086578	7.483216786	7.845892953	7.985313685	sigma-E factor regulatory protein RseB
VVMO6_03217	6.973159505	6.973489731	6.831410813	5.845389545	6.47172443	6.411692864	thiol:disulfide interchange protein
VVMO6_01535	8.899874406	8.735134178	8.713334074	7.90030431	8.258715169	8.216893736	ABC transporter ATPase
VVMO6_RS15045	7.721531784	7.904432682	7.849917689	7.085551985	7.165381095	7.283946157	hypothetical protein
VVMO6_03728	6.048936108	6.176634392	6.480096257	5.207580406	5.777871402	5.777481518	hypothetical protein

VVMO6_04522	8.698187151	8.219640074	8.50258086	8.155480224	7.584628328	7.747535644	molecular chaperone GroEL
VVMO6_02044	5.460655901	6.15911245	6.192655443	5.297799173	5.406148787	5.338578352	membrane protein
VVMO6_04164	5.475834799	5.092347531	5.366489908	4.4414454	4.795927587	4.828112408	prolyl endopeptidase
VVMO6_RS15040	7.046157737	7.184725244	7.240581328	6.393916118	6.541977496	6.660610803	glutathione synthetase
VVMO6_02525	7.466591201	7.475153523	7.344871116	6.582444033	6.959918695	6.852450151	thiol:disulfide interchange protein
VVMO6_00197	7.956627132	7.983522861	7.686729561	6.804658249	7.584628328	7.287746733	thiol:disulfide interchange protein DsbD
VVMO6_00535	6.046904814	6.071046661	6.377582879	5.450843479	5.635677492	5.609491376	LuxR family transcriptional regulator
VVMO6_02869	8.211819717	8.064305969	7.779215466	7.139100937	7.624330141	7.451431021	type II secretion system protein GspF
VVMO6_02865	6.704709899	6.521716468	6.177026028	5.951827408	5.758788183	5.947287288	type II secretion system protein GspJ
VVMO6_02108	7.210338909	7.058824168	7.045827438	6.461537772	6.399635522	6.652773221	o-succinylbenzoate synthase
**Genes expressed less in the WT *V. vulnificus* than in the *ΔrtxA1 V. vulnificus* upon dTHP-1 cell infection**							
VVMO6_01815	8.543619672	8.658784372	8.713542436	9.245666643	9.237773026	9.237903153	Na+-dependent transporter
VVMO6_03418	4.097250023	4.004169522	4.04613488	4.66652924	4.538987939	4.666253298	acetyltransferase
VVMO6_00684	5.38548791	5.204349075	5.221652467	6.128646772	5.555798997	5.864881238	C4-dicarboxylate ABC transporter
VVMO6_00351	5.017612349	4.898032594	4.719244837	5.648432431	5.388520719	5.362801655	diguanylate cyclase
VVMO6_00033	7.288927128	6.936783664	7.023144082	7.910239112	7.260513502	7.824555079	hemolysin III family protein
VVMO6_01713	3.858716566	4.069116926	3.847802207	4.4168782	4.752487473	4.285320742	membrane protein
VVMO6_00121	4.940931065	4.646578868	4.561510043	5.33760902	5.174798295	5.419330168	hypothetical protein
VVMO6_00961	4.67240382	4.180196404	4.077556556	5.256859561	4.719026882	4.719749822	diguanylate cyclase
VVMO6_01522	6.350118787	6.131733085	6.057361801	7.019778009	6.537185594	6.776944898	peptidase S41
VVMO6_01338	6.982754516	7.252387736	7.173701068	7.625358028	8.067019915	7.492628303	abortive infection protein
VVMO6_00616	4.522586209	4.317754659	4.354771323	5.149119837	4.976682907	4.835055206	c-di-GMP phosphodiesterase A
VVMO6_03879	6.612951409	6.432531307	6.4781354	7.257974896	7.06130649	7.036882031	chemotaxis protein
VVMO6_01529	3.849422906	3.948766149	4.051419581	4.66652924	4.493536596	4.475050914	hypothetical protein
VVMO6_02940	3.047197545	2.702219482	2.84473082	3.517959398	3.444622689	3.267958121	molybdopterin biosynthesis protein MoeB
VVMO6_03311	4.368460249	4.016197695	3.947542408	4.697455984	4.558037126	4.855685357	hypothetical protein
VVMO6_02721	3.186569791	3.068466539	3.36114761	3.936159912	3.867455633	3.512757192	uroporphyrin-III methyltransferase
VVMO6_00718	5.066558617	4.930173278	4.861421055	5.826860639	5.477920917	5.348316533	DNA polymerase IV
VVMO6_00980	5.136998578	4.99542432	5.060634771	5.803359761	5.685611961	5.521850801	chemotaxis protein
VVMO6_00186	8.027873173	8.099710099	8.166566915	8.561334722	8.590534568	8.97344691	3-dehydroquinate dehydratase
VVMO6_03410	3.230187677	3.262717353	3.01669044	3.883341388	3.636057611	3.721981798	alkylated DNA repair protein
VVMO6_03225	5.102208461	5.139846816	4.879361724	5.895150114	5.530303902	5.526147125	hydroxymethylpyrimidine/phosphomethylpyrimidine kinase
VVMO6_04098	5.971805594	5.777976107	6.049460175	6.92278217	6.197836844	6.468607006	cytochrome c
VVMO6_01873	5.200508579	4.983220961	4.796811275	5.643146893	5.656448474	5.538959863	hypothetical protein
VVMO6_01759	3.442860102	3.068466539	3.208666768	3.650304897	3.611953695	4.135903741	cysteine desulfurase
VVMO6_04530	2.122195639	2.25143949	2.356842167	2.701073609	2.780478329	2.841228357	N-acetylmuramic acid 6-phosphate etherase
VVMO6_02893	7.7984033	7.768379961	7.678214281	8.424773216	8.220866455	8.480891508	cytochrome c
VVMO6_01981	8.317123721	8.266653315	8.347157248	8.941550568	8.952701215	8.93438056	catalase
VVMO6_03202	3.366734114	2.935446721	3.160570854	3.790771905	3.694612274	3.721981798	endonuclease I
VVMO6_03723	3.639000225	3.725209512	3.766588355	4.287345505	4.194040856	4.448133801	ACP phosphodiesterase
VVMO6_02887	5.261340247	4.855149745	4.92614061	5.947545797	5.437346476	5.508884522	diguanylate cyclase
VVMO6_00064	4.073499448	4.227521539	3.930434809	4.914810371	4.532581903	4.618765314	ABC transporter permease
VVMO6_00053	3.156740908	3.312337799	3.369634329	3.540877366	4.273021903	3.62916973	transcriptional regulator
VVMO6_00637	2.240673931	2.349770678	2.115818326	2.332114458	2.924437563	2.868554117	AraC family transcriptional regulator

VVMO6_00977	2.500221381	2.25143949	2.09549689	2.994631316	2.596178794	2.868554117	amino acid ABC transporter permease
VVMO6_03124	2.678738532	3.179594224	3.291394364	3.471001475	3.772790506	3.721981798	ATPase
VVMO6_01097	4.734301705	4.783066471	5.137188961	5.521678811	5.697114266	5.338578352	hypothetical protein
VVMO6_02016	4.909974065	4.804031807	4.831015073	5.55583354	5.494495835	5.386624948	molybdopterin guanine dinucleotide biosynthesis protein MoaE
VVMO6_01016	3.538489366	3.360308181	3.386459449	4.31419918	3.975309288	3.780697056	choline dehydrogenase
VVMO6_03966	5.129339619	5.052021009	4.885292709	5.684898726	5.474582959	5.80602963	alkyl hydroperoxide reductase subunit C
VVMO6_04301	3.272525444	2.641393317	2.782533339	3.671228529	3.498206326	3.308430367	multidrug transporter
VVMO6_04353	2.874699093	2.494364493	2.423572194	3.210121513	3.019125305	3.267958121	multidrug transporter
VVMO6_02394	5.958900006	5.797273439	5.662536569	6.657762341	6.280444173	6.414013065	DNA repair protein RecN
VVMO6_02822	4.322181065	4.500105279	4.409299069	4.958020556	4.943264595	5.221593042	chemotaxis protein
VVMO6_02032	5.537897692	5.452461655	5.472724269	6.241371558	6.131561892	6.028271755	endonuclease III
VVMO6_02709	4.210469606	4.201421679	4.108308435	5.118976518	4.545365656	4.719749822	hypothetical protein
VVMO6_00344	3.340439189	2.972958009	3.369634329	4.052366072	3.498206326	3.956527055	thymidylate kinase
VVMO6_02198	3.063365107	3.021502481	2.904357303	3.809767077	3.09064096	3.809182233	succinate dehydrogenase cytochrome b556 small membrane subunit
VVMO6_03246	5.200508579	5.250901059	5.219306602	5.760068695	6.095074549	5.718632538	soluble cytochrome b562
VVMO6_03950	6.610202723	6.476725251	6.522574265	7.219428399	7.281677513	7.074036392	RTX toxin transporter
VVMO6_03047	6.401895608	6.106639373	6.026834314	7.085551985	6.875117256	6.519056202	GTPase RsgA
VVMO6_00918	2.720117949	2.960562301	3.090423559	3.563436956	3.671474807	3.347798149	DNA repair ATPase
VVMO6_00925	3.671005749	3.360308181	3.300300196	4.4168782	3.694612274	4.043017517	CDS
VVMO6_01982	9.477716663	9.312657179	9.324214005	10.02609562	10.06186775	10.05258791	aminotransferase class III
VVMO6_00261	3.561438061	3.033387598	3.100655849	4.114795208	3.706043327	3.751638108	succinyldiaminopimelate aminotransferase
VVMO6_02593	7.043103841	6.870418365	6.656918066	7.709944253	7.311917677	7.56749044	cytochrome c
VVMO6_02543	3.550009343	3.520884722	3.559638039	4.189214466	4.369629356	3.917811199	hypothetical protein
VVMO6_01814	5.420426711	5.308278452	5.428729849	6.082532148	5.931847693	6.144611337	chemotaxis protein
VVMO6_02553	6.495726503	6.243390032	6.242023553	7.139100937	6.895229927	6.97379815	dihydropyrimidine dehydrogenase subunit A
VVMO6_02443	4.188525368	4.356115326	4.087880187	4.906010832	5.162404253	4.429905944	hypothetical protein
VVMO6_00622	5.597400473	5.508884522	5.701715109	6.306345435	6.303202807	6.223452232	2-deoxyribose-5-phosphate aldolase
VVMO6_01255	5.851275768	6.455901835	6.2683593	6.75896541	7.203181993	6.650807154	integration host factor subunit alpha
VVMO6_02457	3.538489366	3.739790608	3.727505295	4.4168782	4.280687307	4.275146383	copper homeostasis protein CutC
VVMO6_01530	3.722819286	4.057523708	3.747179168	4.535732918	4.619776931	4.335143291	macrodomain Ter protein
VVMO6_00232	4.419594436	3.824311638	4.061931261	4.888249083	4.811886233	4.634768952	dihydroorotate dehydrogenase
VVMO6_01523	7.876541326	7.698721319	7.585631298	8.532323496	8.304553591	8.410012338	cytochrome c oxidase, cbb3-type subunit I
VVMO6_02179	2.546963893	2.802859233	2.832503993	3.266208147	3.444622689	3.328248535	hypothetical protein
VVMO6_00457	6.762792433	6.283751744	6.307535116	7.425890709	7.070179802	6.938775608	5-formyltetrahydrofolate cyclo-ligase
VVMO6_02563	3.639000225	3.688098702	3.52206474	4.246103874	4.362423399	4.180196404	CDS
VVMO6_03156	2.740370936	2.746213141	2.76976547	3.671228529	3.191677038	3.267958121	membrane protein
VVMO6_02652	7.134892033	6.844511811	6.673292967	7.743672538	7.391066101	7.619686206	3-isopropylmalate dehydratase large subunit
VVMO6_01004	3.628171964	3.49533265	3.483486662	4.327440873	4.127587209	4.113236806	dihydrolipoamide dehydrogenase
VVMO6_01514	6.102727054	5.758417153	5.827176679	6.830465265	6.35857656	6.567800699	universal stress protein E
VVMO6_00260	4.28819132	4.222339204	4.093014431	5.056732011	4.570598269	4.973630161	N-succinylglutamate 5-semialdehyde dehydrogenase
VVMO6_04020	1.888101831	1.744585522	2.011221732	2.659793989	2.266298091	2.428926024	cytochrome b561
VVMO6_00120	2.592239381	2.511460794	2.439783345	3.540877366	2.943878811	2.841228357	RNA polymerase subunit sigma
VVMO6_00166	6.353409852	6.756726781	7.023144082	7.713731016	7.118030836	7.424065352	anti-RNA polymerase sigma 70 factor
VVMO6_00479	4.945299763	4.917402799	5.068475596	5.826860639	5.722663977	5.433122868	hypothetical protein

VVMO6_00354	5.950231693	5.74402404	5.751269039	6.540416877	6.578196008	6.420951361	lysine-sensitive aspartokinase 3
VVMO6_01520	6.906388318	6.718588949	6.590402005	7.615945767	7.329577378	7.383202919	cytochrome CBB3
VVMO6_01729	5.178411134	4.920606032	4.937602116	5.934624191	5.487888701	5.680594383	23S rRNA (adenine(1618)-N(6))-methyltransferase
VMO6_t00087	4.188525368	4.069116926	3.798367774	4.747571404	4.564331368	4.800002196	tRNA
VVMO6_03372	3.526876661	3.147145368	3.180002047	3.901163288	3.984725833	3.930832268	hypothetical protein
VVMO6_01965	2.059096069	1.855858079	1.64240778	2.222981043	2.384839194	2.535060281	4Fe-4S ferredoxin
VVMO6_00893	3.584027423	3.272778914	3.208666768	3.901163288	4.194040856	3.917811199	aquaporin
VVMO6_01760	2.657595075	2.577889315	2.423572194	3.446936634	2.963061555	3.139247826	agglutination protein
VVMO6_01169	3.595190837	3.49533265	3.344022917	4.524278508	3.805040677	4.078554336	hypothetical protein
VVMO6_04314	4.267406327	3.998117619	3.779384248	4.870265932	4.696280751	4.561311786	alkaline phosphatase
VVMO6_04160	6.996502279	6.835206183	6.900391628	7.818262465	7.390180582	7.662273032	ATP-dependent protease
VVMO6_03310	5.586254211	5.814595258	5.903678836	6.574135975	6.444685012	6.461894679	MULTISPECIES: purine-nucleoside phosphorylase
VVMO6_00496	6.749114371	6.708431602	6.752490452	7.603753353	7.402528655	7.364130375	membrane protein
VVMO6_00060	6.634754375	6.650768577	6.489860761	7.453329291	6.977712264	7.46939061	cytochrome c
VVMO6_01880	2.592239381	3.079972504	3.130924362	3.320195692	3.659765417	3.809182233	transcriptional regulator
VVMO6_01712	5.17096946	4.910974918	4.715920614	5.92594504	5.413140142	5.584989724	catalase peroxidase
VVMO6_00219	4.195877264	3.917041582	4.167912928	4.983338794	4.369629356	4.973630161	fumarate reductase flavoprotein subunit
VVMO6_01879	2.350156526	2.291579728	2.756883596	2.701073609	3.223842232	3.308430367	short-chain dehydrogenase
VVMO6_01910	2.657595075	2.25143949	2.663338433	3.121678429	3.403087109	2.895371913	membrane protein
VMO6_t00025	4.468978115	4.069116926	4.229821269	4.897157291	5.215359253	4.734677136	tRNA
VVMO6_01728	2.500221381	2.123848582	2.155622118	3.090950061	2.596178794	2.947556778	hypothetical protein
VVMO6_03482	2.152739794	2.078681194	1.826288971	2.481741662	2.571393471	2.726435417	membrane protein
VVMO6_04029	3.515169722	3.775611353	4.056684995	4.379224731	4.790568568	4.264899761	histone acetyltransferase
VVMO6_RS10310	4.60220813	4.587344567	4.739030908	5.533153799	5.42009778	5.111535313	hypothetical protein
VVMO6_00678	4.640429642	4.147761243	4.128451458	5.01641977	5.103114451	4.967324603	glycerol kinase
VVMO6_00657	2.296453525	2.625777448	2.548430534	3.372235615	2.864490689	3.226317647	amino acid permease
VVMO6_01588	1.517572489	1.715372704	1.64240778	1.835115512	2.235075834	2.353567877	hypothetical protein
VVMO6_00466	2.350156526	2.494364493	2.471669193	2.926649987	3.207849274	3.046583714	hypothetical protein
VVMO6_03405	4.667123759	4.206679561	4.512535931	5.468881031	5.211354091	4.848841366	ribosomal-protein-L7/L12-serine acetyltransferase
VVMO6_03427	5.591838107	5.527912488	5.316885264	6.205962026	6.305083253	6.133390581	GntR family transcriptional regulator
VVMO6_01521	2.856258276	3.136164846	2.950345366	4.003704891	3.270784113	3.69170305	cytochrome-c oxidase
VVMO6_02275	2.856258276	2.909886152	2.649465582	3.71218685	3.403087109	3.308430367	Fe3+-citrate ABC transporter substrate-binding protein
VVMO6_02972	5.943696108	5.977253611	5.613358696	6.900804576	6.418866867	6.414013065	amino acid ABC transporter substrate-binding protein
VVMO6_RS06785	3.550009343	3.817454298	3.529658092	4.429214093	4.576838064	3.943736864	hypothetical protein
VVMO6_04391	2.699576591	2.477063164	2.423572194	3.42246356	2.780478329	3.328248535	hypothetical protein
VVMO6_01794	3.821177222	3.904153811	3.913121909	4.767140076	4.202136397	4.778554161	membrane protein
VVMO6_03387	6.081053757	6.314516953	5.995646874	7.079694916	6.718847457	6.840424727	peptidyl-prolyl cis-trans isomerase
VVMO6_02412	2.657595075	2.960562301	2.578026856	3.691853036	3.301251072	3.247288112	potassium transporter Kef
VVMO6_03026	2.152739794	2.31123866	2.423572194	3.151765932	3.142043299	2.428926024	hypothetical protein
VVMO6_02338	6.676082377	6.48107109	6.533001182	7.518283195	7.156034553	7.263505572	LOG family protein
VVMO6_02020	6.231719243	6.338665316	6.548034334	7.377376497	7.197119005	6.762450749	cyclic pyranopterin phosphate synthase MoaA
VVMO6_03013	2.26883329	2.230942642	2.053974591	3.293454441	2.467761124	2.633918791	hypothetical protein
VVMO6_01877	2.090990802	2.73169714	2.286875307	3.210121513	3.037339308	2.921700285	chromosome partitioning protein ParA
VVMO6_00315	5.366067619	5.278627271	5.244903685	6.184291489	5.943966994	6.003834127	bifunctional protein ArgH

VVMO6_02633	3.904307502	3.844690237	3.792067646	4.634924975	4.466906681	4.610696427	PTS mannitol transporter subunit IIABC
VVMO6_01864	5.030005476	4.790088823	4.917484326	5.621808942	5.714197583	5.633583851	ATP phosphoribosyltransferase
VVMO6_04187	2.818654071	2.494364493	2.39059296	3.517959398	3.037339308	3.161512859	arginine transporter permease subunit ArtM
VVMO6_00920	3.467368217	3.388342995	3.544725939	4.31419918	4.194040856	4.066805723	hypothetical protein
VVMO6_03964	2.296453525	2.055555906	1.850744116	2.961041034	2.596178794	2.535060281	hydroxymethylglutaryl-CoA lyase
VMO6_t00108	6.228138175	6.056560011	5.888994715	7.003370792	6.89772441	6.565715269	tRNA
VVMO6_00205	2.874699093	2.83036948	2.717533114	3.471001475	3.301251072	3.706921858	p pilus assembly/Cpx signaling pathway, periplasmic inhibitor/zinc- resistance associated protein
VVMO6_00974	4.425860634	3.917041582	4.087880187	5.032680084	4.532581903	5.094277834	ATP-binding protein
VVMO6_04368	3.595190837	2.883864539	3.255210877	4.129989335	3.659765417	4.135903741	3-phosphoglycerate dehydrogenase
VVMO6_03935	3.538489366	3.158042947	3.344022917	4.327440873	4.066818212	3.795009946	phosphodiesterase
VVMO6_01623	1.888101831	1.959159509	2.011221732	2.481741662	2.596178794	2.633918791	sigma-54-dependent Fis family transcriptional regulator
VVMO6_02594	7.106891436	7.061003041	6.887891102	7.82877649	7.835521777	7.728087	cytochrome b
VVMO6_RS17365	4.759328712	4.469812481	4.461840735	5.572612393	5.395597815	4.941823108	peptide chain release factor A
VVMO6_04087	5.121639783	5.03142701	4.806220292	5.98980062	5.656448474	5.597292564	membrane protein
VVMO6_01504	3.230187677	2.98524812	3.386459449	4.189214466	3.728637252	3.878027655	NAD-dependent deacylase
VVMO6_00855	8.984293211	8.946900178	9.001893406	9.599886139	9.683691503	9.979863314	long-chain fatty acid transporter
VVMO6_01528	1.993109964	2.032053891	2.19435715	2.818266138	2.780478329	2.568771429	hypothetical protein
VVMO6_03882	3.272525444	3.056868072	3.282433214	3.828515397	4.012612219	3.917811199	sn-glycerol-3-phosphate transporter
VVMO6_01238	1.420498597	1.269387137	1.259644843	1.678586963	2.032063925	1.674669482	hydrolase
VVMO6_02653	5.316341436	5.250901059	5.099419584	6.014571948	5.828431256	6.141814319	3-isopropylmalate dehydratase small subunit
VVMO6_02234	1.369397021	1.559716736	1.750333358	2.278579436	2.170528045	2.05368977	membrane protein
VVMO6_01836	2.856258276	2.746213141	2.950345366	3.607524642	3.624055992	3.441759581	hypothetical protein
VVMO6_03752	6.61432379	6.55919422	6.424159028	7.441198746	7.177749699	7.33992956	2OG-Fe(II) oxygenase
VVMO6_RS16685	1.651936748	1.685556115	1.330912452	2.278579436	2.266298091	1.793488015	hypothetical protein
VVMO6_01863	4.754357923	4.696018761	4.761776831	5.533153799	5.590134963	5.414703142	histidinol dehydrogenase
VVMO6_04374	1.469851859	1.457566192	1.585258133	1.835115512	2.413012738	1.793488015	two-component system response regulator
VVMO6_04019	2.780043411	2.055555906	2.407176811	3.320195692	3.286098016	2.665421495	protoheme IX farnesyltransferase
VVMO6_03060	6.504634486	6.531177574	6.243178638	7.59145702	6.916296806	7.107393388	ribosomal protein S12 methylthiotransferase RimO
VVMO6_01015	2.760343538	2.883864539	2.649465582	3.563436956	3.255305909	3.645058999	coniferyl-aldehyde dehydrogenase
VMO6_t00060	3.712604378	3.529302489	3.090423559	4.246103874	4.625808045	3.580424151	tRNA
VVMO6_01003	2.760343538	2.511460794	2.439783345	3.517959398	3.142043299	3.183439494	hypothetical protein
VVMO6_02242	3.572776954	3.602930625	3.673690976	4.259982492	4.486925058	4.373798436	hypothetical protein
VVMO6_03338	2.059096069	2.625777448	2.578026856	2.926649987	3.471663264	2.947556778	helix-turn-helix transcriptional regulator
VMO6_r00026	8.87203509	9.078735618	8.68428764	9.594077013	10.22433277	9.063768713	rRNA
VVMO6_03103	1.851329095	1.559716736	1.64240778	2.617298363	2.067945619	2.232593149	peptide ABC transporter substrate-binding protein
VVMO6_01886	1.469851859	1.744585522	2.011221732	2.383733795	2.384839194	2.465178616	biotin synthase
VVMO6_02404	1.888101831	1.492426226	1.614115922	2.573512908	2.203162902	2.05368977	hypothetical protein
VVMO6_01979	4.67240382	4.273343436	4.262087882	5.324460751	5.277974323	4.992383144	transcriptional regulator
VVMO6_01634	2.401932016	2.008162671	2.286875307	2.818266138	3.000678407	2.895371913	sugar transferase
VVMO6_03691	4.764282434	4.028126414	4.187246143	5.382706632	4.918911368	5.128588797	serine protease
VVMO6_01876	3.340439189	3.817454298	3.624898342	4.203648506	4.545365656	4.373798436	cyclopropane-fatty-acyl-phospholipid synthase
VVMO6_02017	2.636137134	2.055555906	2.155622118	3.05955293	2.520507619	3.308430367	molybdopterin synthase sulfur carrier subunit
VVMO6_RS09355	2.92864808	2.909886152	3.01669044	3.809767077	3.739802895	3.563802622	hypothetical protein

VVMO6_03030	2.323554891	2.56156673	2.856854896	3.540877366	3.331087912	3.161512859	diguanylate cyclase
VVMO6_00307	5.772333356	5.998629809	6.112758042	7.001306708	6.493326814	6.862678474	5,10-methylenetetrahydrofolate reductase
VVMO6_RS06465	1.851329095	1.959159509	2.155622118	2.701073609	2.736563341	2.601712809	protein-S-isoprenylcysteine methyltransferase
VVMO6_03732	3.379704012	2.83036948	3.180002047	3.987112798	3.826147116	3.917811199	ATP-dependent endonuclease
VVMO6_RS19845	1.369397021	1.592220486	1.259644843	1.835115512	2.235075834	1.793488015	hypothetical protein
VVMO6_01343	1.420498597	1.457566192	1.826288971	2.042043814	2.440646619	2.05368977	hypothetical protein
VVMO6_02444	2.92864808	3.469319844	3.57439758	4.129989335	4.460171683	3.736886154	hypothetical protein
VVMO6_02942	6.189990314	5.843573289	5.807009536	6.873981749	6.763296759	6.745968858	phosphomethylpyrimidine synthase ThiC
VVMO6_01693	1.261420515	1.143444588	1.222645824	1.907439984	1.661260139	1.735301621	tricarboxylic transport TctC
VVMO6_00604	1.851329095	2.31123866	2.074885118	2.891419063	3.125111866	2.189903884	hypothetical protein
VMO6_t00055	4.693332916	5.201718279	4.821766896	5.616424776	6.261200489	5.194855672	tRNA
VVMO6_03230	1.694080855	1.959159509	1.898444857	2.659793989	2.520507619	2.465178616	MFS transporter
VVMO6_RS08370	1.923960519	1.773218537	2.17511963	2.994631316	2.384839194	2.633918791	acylphosphatase
VVMO6_01532	8.344670168	8.559794722	8.628141988	9.200052103	9.39103795	9.52709436	beta-hydroxydecanoyl-ACP dehydratase
VVMO6_02477	6.780015397	6.512192908	6.777420656	7.741201009	7.447489286	7.457067447	ATP-dependent chaperone ClpB
VVMO6_01280	2.980652177	2.909886152	2.832503993	3.809767077	3.826147116	3.367086388	integron integrase
VVMO6_02033	5.855924117	5.648729633	5.743127351	6.88968894	6.295656416	6.596684541	lactoylglutathione lyase
VVMO6_01268	8.081448463	8.110619964	8.125189795	8.837218687	8.909166304	9.159524021	hypothetical protein
VVMO6_03358	3.595190837	3.200828295	3.227464984	4.27372887	4.08444354	4.101768375	acyl-CoA thioesterase
VVMO6_02509	4.645808126	4.529775067	4.315776597	5.572612393	5.341653503	5.094277834	sodium:phosphate symporter
VVMO6_00881	3.886243645	3.831136539	3.986688467	4.66652924	4.817166792	4.704666443	two-component system response regulator
VVMO6_00446	2.963524921	3.021502481	3.309151389	3.970327661	3.826147116	3.956527055	hypothetical protein
VVMO6_03819	2.350156526	2.271649208	2.268840241	3.05955293	3.037339308	2.997919733	hemolysin D
VVMO6_02519	2.760343538	2.40570522	2.756883596	3.563436956	3.611953695	2.997919733	hypothetical protein
VVMO6_02632	3.379704012	2.909886152	3.069738653	4.052366072	4.057924137	3.645058999	ABC transporter substrate-binding protein
VVMO6_01140	5.813539751	5.753036549	5.767415858	6.876236131	6.444685012	6.614951944	fumarate hydratase
VVMO6_RS04830	1.851329095	2.271649208	2.621313149	3.471001475	2.780478329	2.841228357	MULTISPECIES: hypothetical protein
VVMO6_01160	5.537897692	5.358745345	5.360115645	6.511702821	5.972644566	6.362086294	hypothetical protein
VVMO6_02948	3.503367008	3.57880303	3.300300196	4.727733648	4.210186764	3.864518889	transposase
VVMO6_02884	3.526876661	3.125100108	3.038144358	4.4168782	3.805040677	3.943736864	hypothetical protein
VVMO6_02915	5.633042334	5.196442251	5.478620891	6.585202788	6.131561892	6.24966958	hypothetical protein
VVMO6_RS19600	7.077341812	7.477874166	7.605976787	8.030493591	8.771437414	7.955359881	MULTISPECIES: peptide chain release factor 1
VVMO6_00730	6.320156401	6.44259364	6.598547759	7.19435149	7.462731767	7.404362395	aminobenzoyl-glutamate transporter
VVMO6_03741	7.434320554	7.424719762	7.682478203	8.351317732	8.340753864	8.569418377	long-chain fatty acid outer membrane transporter
VVMO6_00985	10.35730592	10.40489918	10.60567009	11.47387111	11.33760148	11.29189486	membrane protein
VVMO6_01964	1.608524383	1.801294317	1.724099397	2.481741662	2.326785812	2.465178616	formate-dependent nitrite reductase subunit NrfD
VVMO6_00940	3.991382301	3.961263186	4.153241074	5.07254671	4.741419947	4.922396741	chemotaxis protein
VVMO6_04184	3.417928445	3.42489493	3.52206474	4.429214093	4.185899631	4.325315479	arginine ABC transporter ATP-binding protein
VVMO6_00976	2.427139237	2.008162671	2.17511963	3.121678429	2.714094175	3.070313671	histidine/lysine/arginine/ornithine ABC transporter permease HisQ
VVMO6_00420	3.063365107	3.009518638	3.059283911	4.189214466	3.794370522	3.62916973	chemotaxis protein
VVMO6_00259	4.105080781	3.739790608	3.853864089	5.056732011	4.513191336	4.734677136	arginine N-succinyltransferase
VVMO6_01715	5.823066694	5.389124535	5.353713093	6.787991942	6.314448893	6.150189157	diguanylate cyclase
VVMO6_00924	4.677664627	4.765359575	4.923260949	5.8128061	5.807358453	5.437691287	integrase
VVMO6_02928	2.892907166	2.948059165	3.180002047	3.372235615	4.218192459	3.809182233	membrane protein

VVMO6_03975	2.546963893	1.828834138	2.268840241	3.151765932	3.055326224	2.813375016	sugar phosphate antiporter
VVMO6_04389	2.090990802	1.685556115	1.525750898	2.278579436	2.62054549	2.465178616	5-methyltetrahydropteroyltriglutamate--homocysteine methyltransferase
VVMO6_01875	1.774846348	1.624008037	1.585258133	2.573512908	2.296858936	2.232593149	zinc ABC transporter permease
VVMO6_03940	1.694080855	1.347627929	1.259644843	2.278579436	1.957506916	2.05368977	branched-chain alpha-keto acid dehydrogenase subunit E2
VVMO6_00635	1.082602705	1.309037866	1.585258133	1.758973063	2.235075834	1.903261058	hypothetical protein
VVMO6_03740	7.998219532	7.861742486	8.217114316	8.919312695	8.864914248	9.122052024	lipase
VVMO6_03805	4.744364666	4.370241625	4.417508	5.836154838	5.12882329	5.242631796	multidrug transporter
VVMO6_03955	1.813594492	1.347627929	1.184672906	2.383733795	2.067945619	1.903261058	enoyl-CoA hydratase
VVMO6_04431	2.569779237	2.167644708	1.801412127	2.573512908	2.884751013	3.367086388	peptide-methionine (S)-S-oxide reductase
VVMO6_02008	3.550009343	3.433890025	3.273416055	4.634924975	3.965830876	4.275146383	glutathione-dependent reductase
VVMO6_01745	1.813594492	1.959159509	1.670155476	2.701073609	2.413012738	2.601712809	nucleoside-diphosphate sugar epimerase
VVMO6_01931	1.017707389	1.624008037	1.222645824	1.835115512	1.957506916	2.100538084	outer membrane beta-barrel domain protein
VVMO6_02595	6.513487804	6.465803034	6.162447029	7.407304045	7.39460275	7.188237966	ubiquinol-cytochrome c reductase
VVMO6_00350	5.600173635	5.372846041	5.422625304	6.647240347	6.329308591	6.218151101	hypothetical protein
VVMO6_04170	4.406979793	4.125725463	3.913121909	5.357109437	4.746964323	5.094277834	lactate dehydrogenase
VVMO6_03596	4.651166632	4.327440873	4.668558868	5.88174837	5.296997022	5.210957482	hypothetical protein
VVMO6_00963	4.635031033	4.201421679	4.311378065	5.62717309	5.239159503	5.053188976	hypothetical protein
VVMO6_00624	5.167234179	4.881689688	4.821766896	5.877253313	5.825813927	6.009982451	phosphopentomutase
VVMO6_RS17980	3.922147978	3.789694276	4.138418477	4.861189592	4.466906681	5.232150769	hypothetical protein
VVMO6_01781	4.322181065	4.045836574	4.225152272	5.317841407	4.971955995	5.111535313	acyl-CoA dehydrogenase
VVMO6_03182	4.368460249	4.346620359	4.067158519	5.178646217	5.281798982	5.17310317	membrane protein
VVMO6_03215	2.350156526	3.113949852	3.282433214	3.771523293	4.202136397	3.563802622	1,4-alpha-glucan-branching protein
VVMO6_03408	6.568331359	6.289957494	6.120321369	7.583200851	7.216231704	7.121659284	nucleoside permease NupC
VVMO6_01749	3.340439189	3.331717773	3.411334843	4.391885394	4.280687307	4.191060308	DNA helicase
VVMO6_03926	3.948501734	3.57880303	3.352610672	4.580662226	4.673170242	4.466134228	acetoacetyl-CoA synthetase
VVMO6_02405	1.813594492	2.055555906	1.92171654	2.383733795	2.780478329	2.947556778	D-alanine--D-alanine ligase
VVMO6_00032	4.387848575	4.469812481	4.493248015	5.664173843	5.274139498	5.328773991	hypothetical protein
VVMO6_01098	2.614355214	2.368657512	2.39059296	3.397568165	3.360320169	3.205037862	ABC transporter substrate-binding protein
VVMO6_00017	3.313656094	3.252585127	3.199175002	4.203648506	4.04897489	4.305456699	Bcr/CflA family drug resistance efflux transporter
VVMO6_00394	4.504937254	4.416354635	4.550354983	5.605595783	5.094442013	5.665093789	arginine deiminase
VVMO6_01156	5.391903679	5.191146857	5.606194415	6.365312457	6.387262556	6.459650279	glycine radical enzyme, YjjI family
VVMO6_01490	2.980652177	2.702219482	2.856854896	3.936159912	3.524269851	3.823216652	hypothetical protein
VVMO6_00690	3.215794411	3.102712748	3.236773031	4.036327357	3.946685163	4.364231314	N-acetylglutamate synthase
VVMO6_01758	5.885778724	5.633193399	5.604397772	7.205152154	6.301319907	6.510408192	hypothetical protein
VVMO6_03514	2.760343538	2.56156673	2.730769557	3.585649203	3.562502994	3.62916973	ribokinase
VVMO6_04155	3.69195499	3.618794394	3.005842644	4.824302207	4.218192459	4.180196404	multidrug transporter
VVMO6_01544	6.597768417	6.119240787	5.992902789	7.640025764	7.297821487	6.796639532	hypothetical protein
VVMO6_01756	3.584027423	3.460543626	3.552201256	4.757388918	4.33323416	4.420704918	di-and tricarboxylate transporter
VVMO6_03953	3.526876661	3.179594224	2.961616925	4.404435917	3.794370522	4.335143291	3-ketoacyl-ACP reductase
VVMO6_00150	4.342197054	3.810564209	3.773000488	5.382706632	4.832893483	4.674018186	endonuclease DDE
VVMO6_04137	4.158737311	4.158654189	4.248347604	5.214724183	5.250913991	5.117242235	LysR family transcriptional regulator
VMO6_t00065	1.369397021	1.186658618	0.978192013	2.165153826	1.8788859	1.475798496	tRNA
VVMO6_04002	2.523781933	2.368657512	2.423572194	3.446936634	3.331087912	3.267958121	ClC family H(+)/Cl(-) exchange transporter
VVMO6_04338	1.694080855	2.101441641	2.232079316	3.210121513	2.943878811	2.568771429	hypothetical protein

VVMO6_00054	8.146447142	8.404303081	8.56210628	9.288614174	9.394574668	9.64437809	ketol-acid reductoisomerase
VVMO6_01840	2.546963893	1.908433184	2.074885118	3.320195692	2.823095958	3.116633784	cytochrome C nitrate reductase, partial
VVMO6_03720	3.030846746	2.816679928	3.100655849	4.145025107	3.965830876	3.795009946	haloacid dehalogenase
VVMO6_03186	8.969355958	9.266985659	9.478323696	10.14702012	10.48291538	10.35930992	hypothetical protein
VVMO6_03157	2.182650666	2.210150386	2.09549689	3.238437368	2.924437563	3.093659617	PTS fructose transporter subunit IIC
VVMO6_01663	2.856258276	2.656841967	2.807735009	3.847023198	3.599749017	3.823216652	amino acid ABC transporter substrate-binding protein
VVMO6_02475	5.140812864	4.351375653	4.252942253	6.117255567	5.348963688	5.530430694	penicillin-insensitive murein endopeptidase
VVMO6_04116	3.671005749	3.747026262	3.544725939	4.634924975	4.637795157	4.821136036	preprotein translocase subunit SecF
VVMO6_03978	2.678738532	2.717033597	2.76976547	4.145025107	3.444622689	3.546987359	hypothetical protein
VVMO6_RS08220	2.636137134	2.672126941	2.677079153	3.650304897	3.524269851	3.809182233	hypothetical protein
VVMO6_RS10980	2.592239381	2.997434416	2.972801103	3.691853036	4.169478047	3.721981798	succinate dehydrogenase cytochrome b556 large membrane subunit
VVMO6_00938	4.635031033	4.59938759	4.977015163	5.798613296	5.79670549	5.940865277	uridine phosphorylase
VVMO6_02959	3.782634939	4.164069974	4.252942253	5.193185847	5.170678767	5.139846816	hypothetical protein
VVMO6_04339	2.296453525	2.101441641	2.053974591	3.238437368	2.904730749	3.139247826	hypothetical protein
VVMO6_03286	2.240673931	2.101441641	2.621313149	3.210121513	3.255305909	3.459839217	hypothetical protein
VVMO6_03959	1.735028708	1.186658618	1.670155476	2.701073609	2.137137844	2.314359377	acetyl-CoA acetyltransferase
VVMO6_01503	3.030846746	3.045175604	2.868877932	4.232090446	3.975309288	3.809182233	ammonium transporter
VVMO6_01721	2.122195639	1.685556115	1.398824622	2.617298363	2.69126952	2.535060281	2-keto-4-pentenoate hydratase
VVMO6_03426	3.014308509	2.816679928	2.439783345	4.099439355	3.739802895	3.477695083	lipoate--protein ligase
VVMO6_01480	3.753037093	3.397568165	3.646011852	4.83361285	4.655591122	4.535970721	ABC transporter ATP-binding protein
VVMO6_03962	1.651936748	1.492426226	1.431618337	2.85530621	1.8788859	2.353567877	methylcrotonoyl-CoA carboxylase
VVMO6_00419	4.878338203	4.317754659	4.421595017	6.030852984	5.638663147	5.308963089	chemotaxis protein
VVMO6_04559	1.316418701	1.143444588	1.105590343	1.976311152	2.235075834	1.475798496	anti-anti-sigma regulatory factor
VMO6_t00088	1.517572489	1.773218537	1.874791623	2.891419063	2.843941784	2.100538084	tRNA
VVMO6_01304	1.204242316	1.269387137	1.525750898	1.758973063	2.520507619	2.005268955	hypothetical protein
VVMO6_04185	3.802034786	3.878027655	3.964449519	5.06466103	4.655591122	5.247843893	arginine ABC transporter substrate-binding protein
VVMO6_03612	4.335555866	4.474179162	4.579912253	5.872744207	5.293212495	5.621587905	membrane protein
VVMO6_04430	2.636137134	2.6099907	2.21334152	3.42246356	3.331087912	3.766240745	peptide-methionine (R)-S-oxide reductase
VVMO6_00623	4.918886912	4.721954726	4.437828275	6.078621891	5.623672697	5.823589523	thymidine phosphorylase
VVMO6_01482	5.659872222	5.500346418	5.775422	6.993020712	6.665685463	6.785930347	tungsten ABC transporter substrate-binding protein
VVMO6_01612	2.323554891	2.291579728	2.471669193	3.372235615	3.611953695	3.116633784	hypothetical protein
VVMO6_00780	3.628171964	3.50390039	3.427683307	4.697455984	4.33323416	4.855685357	acyl-CoA dehydrogenase
VVMO6_03486	4.224915828	3.672982896	3.603471246	5.242950722	5.085717127	4.569660849	hypothetical protein
VVMO6_02219	6.338540575	6.192621759	6.304218562	7.536927526	7.488611012	7.360526032	asparagine synthase B
VVMO6_00418	3.877126186	3.618794394	3.680528571	5.178646217	4.889134253	4.429905944	flavoprotein
VVMO6_03957	1.469851859	0.620462691	1.222645824	2.165153826	1.707490887	1.849418349	acyl-CoA dehydrogenase
VVMO6_01881	5.193180307	5.14533353	5.202777863	6.384444437	6.345844821	6.342730638	protease HtpX
VVMO6_04229	1.144704123	1.228615784	1.614115922	2.332114458	1.288034345	2.696250979	PTS ascorbate transporter subunit IIB
VVMO6_03416	3.126282244	3.478042998	3.344022917	4.340562132	4.64375157	4.325315479	methionine sulfoxide reductase
VVMO6_04222	1.316418701	1.269387137	1.398824622	2.222981043	1.752286067	2.535060281	tryptophanase
VVMO6_04340	2.122195639	1.855858079	1.92171654	2.961041034	2.98199258	2.921700285	hypothetical protein
VVMO6_01930	3.639000225	3.695597626	3.829462223	4.897157291	4.928702045	4.848841366	patatin family protein
VVMO6_04138	7.306013433	7.263152336	6.905507586	8.370634959	8.456946288	8.372328047	adenylosuccinate synthetase
VVMO6_00975	3.743034651	3.442829384	3.038144358	5.040742029	4.241946397	4.402124931	ABC transporter substrate-binding protein

VVMO6_02089	2.546963893	2.545057356	2.795189203	3.883341388	3.624055992	3.766240745	membrane protein
VVMO6_04458	1.204242316	1.228615784	1.145673429	2.481741662	1.661260139	1.955166396	C4-dicarboxylate ABC transporter substrate-binding protein
VVMO6_03714	1.9589495	1.385212596	1.724099397	3.320195692	2.170528045	2.314359377	glucose-1-phosphate adenylyltransferase
VVMO6_03881	2.427139237	2.271649208	2.471669193	3.346450284	3.562502994	3.477695083	cytolysin
VVMO6_04186	2.059096069	2.423878313	2.074885118	3.346450284	3.316246624	3.116633784	arginine transporter permease subunit ArtQ
VVMO6_02235	1.608524383	1.934019282	1.431618337	2.994631316	2.668077947	2.189903884	membrane protein
VVMO6_01655	6.830483966	6.714903637	6.707170645	8.065489081	8.195746719	7.681660507	transporter
VVMO6_04379	1.651936748	1.457566192	1.724099397	3.121678429	2.170528045	2.353567877	DNA-binding response regulator
VMO6_t00095	1.420498597	1.492426226	1.222645824	2.165153826	2.467761124	2.100538084	tRNA
VVMO6_04052	1.517572489	1.098896064	1.105590343	2.481741662	1.957506916	1.849418349	reactive intermediate/imine deaminase
VVMO6_04169	2.122195639	2.230942642	2.356842167	3.71218685	3.107979364	3.183439494	lactate dehydrogenase
VVMO6_01545	5.410981553	4.447778597	4.433787052	6.693996497	5.794029913	5.521850801	hypothetical protein
VVMO6_03183	5.021755232	4.868480745	4.61958937	6.342666345	5.998440786	5.953680838	hypothetical protein
VVMO6_03185	7.435873731	7.184058991	7.342717434	8.737825659	8.470833802	8.646426376	TetR family transcriptional regulator
VVMO6_00314	5.144617092	4.871794336	5.117168411	6.636641049	6.044192973	6.247069181	argininosuccinate synthase
VVMO6_01720	1.017707389	1.052927989	1.259644843	2.528356869	1.795732156	1.545179768	maleylacetoacetate isomerase
VVMO6_02306	4.444497566	4.482873071	4.640780149	5.91282762	5.504350144	5.972693223	hypothetical protein
VVMO6_04117	4.60220813	4.092027568	4.425670487	5.831515223	5.308291398	5.823589523	preprotein translocase subunit SecD
VVMO6_03001	2.699576591	2.687251671	2.832503993	4.099439355	3.636057611	4.078554336	chemotaxis protein
VVMO6_03828	4.754357923	4.587344567	4.61958937	6.286141731	5.536720039	5.953680838	thiol reductase thioredoxin
VVMO6_02957	3.753037093	3.961263186	3.559638039	5.369964804	4.519683859	5.122926671	membrane protein
VVMO6_01932	4.499005961	4.611330914	4.449884463	5.895150114	5.912242529	5.637560435	transporter
VVMO6_00676	4.841312527	4.554729524	5.04482411	5.917213359	6.272777474	6.199442229	glycerol-3-phosphate dehydrogenase
VVMO6_01498	3.479467895	3.282770791	3.435788587	5.008220368	4.249778251	4.689423698	membrane protein
VVMO6_00969	3.063365107	2.477063164	2.423572194	4.003704891	3.956289779	3.660775175	chemotaxis protein
VVMO6_04167	2.799477904	2.672126941	2.286875307	4.052366072	3.54987086	3.795009946	Fe-S oxidoreductase
VVMO6_03325	1.144704123	0.852839618	1.222645824	2.332114458	1.8788859	1.674669482	phosphate ABC transporter substrate-binding protein
VVMO6_01481	3.110808323	2.972958009	3.090423559	4.217939565	4.519683859	4.147105043	ABC transporter permease
VVMO6_00497	5.583454137	5.391435112	5.452892958	6.97840471	6.714609257	6.785930347	RNA polymerase sigma factor RpoS
VVMO6_04067	1.993109964	1.959159509	1.776098787	2.961041034	2.823095958	3.267958121	lactoylglutathione lyase
VVMO6_00936	1.735028708	1.492426226	1.850744116	2.818266138	2.668077947	2.868554117	inosine-5-monophosphate dehydrogenase
VVMO6_01492	4.374952018	4.010196143	4.072366905	5.84077958	5.145711476	5.419330168	(Fe-S)-binding protein
VVMO6_00658	2.026480243	1.773218537	1.398824622	2.891419063	2.596178794	2.997919733	amino acid permease
VVMO6_03958	1.563765053	1.457566192	1.585258133	2.926649987	2.266298091	2.601712809	methylmalonate-semialdehyde dehydrogenase (acylating)
VVMO6_04228	0.803421789	1.005446909	1.398824622	1.976311152	2.067945619	2.05368977	PTS ascorbate transporter subunit IIA
VVMO6_03250	3.491466939	3.570669988	3.218096493	5.193185847	4.039969784	4.807081263	hypothetical protein
VVMO6_00258	5.448396739	5.310722094	5.139672107	6.896368593	6.403151252	6.710542826	acetylornithine aminotransferase
VMO6_r00012	0.640553079	1.005446909	0.313244418	0.938957776	1.837908045	1.067797733	rRNA
VVMO6_01782	2.182650666	2.055555906	2.09549689	3.494671481	3.107979364	3.308430367	TetR family transcriptional regulator
VVMO6_03970	5.60846128	5.134339156	4.993582339	7.259702775	6.387262556	6.067120672	sodium:calcium antiporter
VVMO6_02040	8.810207162	8.919743743	8.930014764	10.59468436	10.25128209	10.14295721	superoxide dismutase
VVMO6_04166	3.503367008	3.252585127	3.059283911	4.897157291	4.325843632	4.658446392	lactate permease
VVMO6_01125	1.694080855	1.655110271	1.898444857	3.090950061	2.884751013	2.895371913	hypothetical protein
VVMO6_04168	2.997578486	2.687251671	2.635458034	4.656071223	3.739802895	3.837115859	(Fe-S)-binding protein

VVMO6_03194	1.369397021	0.956349826	1.365268105	2.701073609	2.032063925	2.100538084	hypothetical protein
VVMO6_03251	4.394253877	4.388864069	3.785739885	6.154881604	5.195221314	5.455821659	membrane protein
VMO6_t00011	1.204242316	1.143444588	0.788682617	1.907439984	2.326785812	1.674669482	tRNA
VVMO6_03954	1.469851859	1.005446909	0.737142981	2.383733795	1.837908045	2.005268955	3-hydroxyisobutyrate dehydrogenase
VVMO6_04354	1.9589495	2.032053891	1.944618785	3.585649203	3.286098016	2.841228357	ferredoxin--NADP(+) reductase
VVMO6_01121	2.476269666	2.167644708	2.502865524	3.650304897	3.826147116	3.706921858	hypothetical protein
VVMO6_00386	4.355388413	3.810564209	3.997680695	5.664173843	5.577743502	5.442245285	ATPase
VVMO6_03979	4.624172827	3.754225808	3.773000488	5.973046907	5.48125117	5.288876354	mannose-6-phosphate isomerase
VVMO6_01722	1.813594492	1.655110271	1.585258133	3.121678429	2.780478329	2.868554117	homogentisate 1,2-dioxygenase
VVMO6_02165	5.00511184	4.986281492	4.689047435	6.818791999	6.289970606	6.119240787	nucleoside permease
VVMO6_RS10920	2.678738532	2.997434416	3.506757076	4.796004231	4.419087759	4.52742381	iron transporter FeoA
VVMO6_04337	2.376276524	2.271649208	2.423572194	3.607524642	3.717384519	3.917811199	elongation factor G
VVMO6_03184	5.995170108	5.483117108	5.615144222	7.516838986	7.234107852	7.113116686	hypothetical protein
VVMO6_03513	1.888101831	1.828834138	1.776098787	3.210121513	2.823095958	3.367086388	D-ribose ABC transporter substrate-binding protein
VVMO6_04375	5.404650251	5.368161105	5.602598889	7.017737282	6.892731124	7.234918348	acyl-CoA synthetase
VVMO6_03666	2.657595075	1.655110271	1.463683159	3.953344934	3.207849274	2.696250979	multidrug MFS transporter
VVMO6_00670	3.286366361	3.190250325	3.236773031	4.805498696	4.480283081	4.973630161	tRNA-binding protein
VVMO6_03134	3.939770529	3.65000802	3.785739885	5.389035593	5.409648699	5.3336845	galactose/methyl galactoside import ATP-binding protein MglA
VVMO6_02075	3.491466939	3.292693941	3.378071416	5.06466103	4.801266774	5.059130947	2,4-dienoyl-CoA reductase
VVMO6_00964	2.837578691	2.788904857	3.120905254	4.656071223	4.493536596	4.315420258	PrkA family serine protein kinase
VVMO6_00764	3.830654028	3.871421515	3.918915991	5.764943464	5.195221314	5.538959863	cytochrome c biogenesis protein CcsB
VVMO6_01256	4.217710799	4.063331962	4.266639047	6.054935138	5.788663826	5.940865277	phosphoribosylglycinamide formyltransferase
VVMO6_RS09805	1.608524383	1.005446909	1.105590343	2.85530621	2.203162902	2.568771429	cysteine hydrolase
VVMO6_01497	3.584027423	3.113949852	3.483486662	5.317841407	4.990771422	5.059130947	formate dehydrogenase
VVMO6_03425	3.156740908	2.896934013	2.455814358	5.156558278	4.194040856	4.101768375	chemotaxis protein
VVMO6_02645	5.755017884	5.169768747	5.511587356	7.460859412	7.190013164	7.31283122	leucine transcriptional activator
VVMO6_04363	2.451913581	2.330633306	2.592600403	4.477529176	3.975309288	3.904671538	alpha/beta hydrolase
VVMO6_01496	2.760343538	2.441825331	2.677079153	4.767140076	4.066818212	4.03097469	formate dehydrogenase
VVMO6_04183	6.514958089	6.294902947	6.142776319	8.446175725	7.609189233	8.365151331	hypothetical protein
VVMO6_01171	3.126282244	2.948059165	3.048752853	4.958020556	4.589237271	4.841964754	hypothetical protein
VVMO6_02966	4.613232279	4.370241625	4.527782724	6.746696514	6.021498239	6.228733955	heat-shock protein Hsp20
VVMO6_03942	0.803421789	0.620462691	0.570460532	1.678586963	1.837908045	1.402911263	electron transfer flavoprotein subunit beta
VVMO6_01495	5.243713291	4.692275265	4.963062355	7.280277495	6.592124596	6.683871274	formate dehydrogenase
VVMO6_03968	5.995170108	5.196442251	5.42669787	7.468350433	7.91339729	6.961160831	cold-shock protein
VVMO6_04479	3.379704012	3.190250325	3.40309061	5.34413849	4.853599229	5.396045195	amidinotransferase
VVMO6_03466	1.082602705	1.457566192	1.69737955	2.573512908	3.286098016	2.997919733	sn-glycerol-3-phosphate dehydrogenase subunit C
VVMO6_04362	0.640553079	0.956349826	0.628187741	1.976311152	2.032063925	1.793488015	hypothetical protein
VVMO6_02535	3.957180416	4.293250725	4.15814829	6.434256344	5.427022025	6.320640541	hypothetical protein
VVMO6_01494	0.724284536	0.852839618	1.431618337	2.926649987	1.8788859	2.500542526	transcriptional initiation protein Tat
VVMO6_00393	2.678738532	3.168858827	3.100655849	5.040742029	4.801266774	4.979908279	ornithine carbamoyltransferase
VVMO6_02378	8.376965459	7.758120793	8.092237785	10.37076385	10.05364225	10.06278984	S-(hydroxymethyl)glutathione dehydrogenase
VVMO6_00312	3.503367008	3.486713724	3.720887263	5.750269461	5.461153357	5.517541643	N-acetyl-gamma-glutamyl-phosphate reductase
VVMO6_02954	3.141591956	2.972958009	2.961616925	5.095948385	4.843283502	5.070941971	3-ketoacyl-CoA thiolase
VVMO6_03465	1.694080855	1.559716736	1.585258133	3.42246356	3.239659843	3.308430367	anaerobic glycerol-3-phosphate dehydrogenase subunit B

VVMO6_01723	0.949754894	1.186658618	1.222645824	2.818266138	2.356104489	2.813375016	4-hydroxyphenylpyruvate dioxygenase
VVMO6_03925	1.993109964	1.592220486	1.184672906	3.446936634	3.286098016	3.247288112	phenylalanine 4-monooxygenase
VVMO6_03665	3.095166632	2.774814189	2.487351679	4.852055788	5.334306089	3.823216652	hypothetical protein
VVMO6_01493	1.651936748	1.526463768	1.365268105	3.563436956	3.142043299	2.947556778	TorD family cytoplasmic chaperone
VVMO6_02953	4.754357923	4.623176177	4.637269897	7.027912171	6.54834204	6.831773352	fatty acid oxidation complex subunit alpha
VVMO6_00313	3.079353494	3.200828295	3.038144358	5.426437152	4.98609052	5.178572121	acetylglutamate kinase
VVMO6_01082	3.030846746	3.360308181	2.011221732	5.947545797	3.717384519	4.49272064	hypothetical protein
VVMO6_00725	4.562946389	4.587344567	4.705901741	6.946577004	6.919982766	6.886266275	hypothetical protein
VVMO6_03424	2.059096069	2.189054083	1.724099397	4.697455984	3.37471714	3.721981798	hypothetical protein
VVMO6_RS15125	1.261420515	1.347627929	1.259644843	2.961041034	2.413012738	3.62916973	hypothetical protein
VVMO6_02451	2.296453525	2.511460794	2.286875307	4.613464161	4.513191336	4.577961872	acetoin utilization protein AcuB
VVMO6_03512	1.469851859	1.624008037	1.145673429	3.471001475	3.175321457	3.864518889	ribose ABC transporter permease
VVMO6_03135	4.381414707	4.558846956	4.661664172	7.133457699	7.119100125	7.184165612	galactose ABC transporter substrate-binding protein
VVMO6_00091	3.2012561	3.09138743	3.246021409	5.745344771	5.708525591	5.766628634	universal stress protein A
VVMO6_02986	5.46978239	5.876936231	5.97215408	8.759969812	8.021425497	8.5859757	hypothetical protein
VVMO6_02379	5.649200067	5.258515752	5.486445751	8.414727369	8.225341922	8.011597267	S-formylglutathione hydrolase
VVMO6_04355	0.803421789	0.905522852	1.02192011	2.926649987	2.758687923	3.022456901	thioesterase
VVMO6_03961	0.456929957	0.905522852	0.447577237	2.481741662	1.918731908	2.568771429	isovaleryl-CoA dehydrogenase
VVMO6_04136	3.886243645	3.322060327	3.491285246	6.641950432	5.617632631	6.608888473	hypothetical protein
VVMO6_00660	2.152739794	2.40570522	2.356842167	5.118976518	4.763470741	4.973630161	beta-galactosidase subunit alpha
VVMO6_03388	1.563765053	1.457566192	1.585258133	4.06822844	3.9174816	3.943736864	universal stress protein A
VVMO6_RS19695	2.427139237	1.228615784	1.495050788	4.697455984	5.063671235	2.921700285	hypothetical protein
VVMO6_03880	1.017707389	0.417763525	1.222645824	3.151765932	3.107979364	3.183439494	cytolysin secretion protein
VVMO6_03464	2.122195639	2.008162671	2.19435715	5.008220368	4.735854182	5.151017663	FAD/NAD(P)-binding oxidoreductase
VVMO6_03164	2.323554891	1.624008037	2.09549689	5.242950722	4.868935823	5.247843893	purine-nucleoside phosphorylase
VVMO6_03810	3.047197545	3.388342995	3.652981577	6.644597814	6.723073243	6.866071864	hypothetical protein
VVMO6_03511	1.369397021	1.624008037	1.69737955	4.737686623	4.690537738	4.841964754	ribose import ATP-binding protein RbsA
VVMO6_00659	0.128497248	0.093791394	0.38197377	2.278579436	2.235075834	2.100538084	beta-D-galactosidase subunit beta
VVMO6_03510	0.724284536	0.852839618	0.933096976	4.591679279	4.202136397	4.43904866	D-ribose pyranase
VVMO6_02482	5.159734477	5.253443758	5.569829434	10.06144434	9.827836885	10.05221556	cold-shock protein
