# Supplementary material for: MARTX Toxin-Stimulated Interplay between Human Cells and Vibrio vulnificus
Source: mSphere. 2020 Aug 12;5(4):e00659-20. doi: 10.1128/mSphere.00659-20 (PMC7426173; doi:10.1128/mSphere.00659-20)
Supplement: TABLE S4 [file mSphere.00659-20-st004.pdf]

Table S4. Differentially regulated pathways in *V. vulnificus* (WT vs. *Δ rtxA1*) during dTHP-1 cell infection

Description	KEGG_path_id	Z_score (WT / RTX)	genes_fold_changes
Biosynthesis of siderophore group nonribosomal peptides	vvm01053	13.869482	VVMO6_02112:0.124298994563; VVMO6_04202:3.05735572503; VVMO6_04206:2.15005097897; VVMO6_04201:2.46257449058; VVMO6_04203:2.99659256302; VVMO6_04212:1.89071026713; VVMO6_04199:2.88753934061; VVMO6_04198:2.73540764519; VVMO6_04207:2.26840812332; VVMO6_04208:2.43220700238;
Ribosome	vvm03010	3.0866872	VVMO6_01920:0.0966463565563; VVMO6_00746:-0.116546100725; VVMO6_02749:0.260676579451; VVMO6_02731:0.107777430093; VVMO6_02738:0.143163243487; VVMO6_00329:0.245739324986; VVMO6_00282:-0.100581652781; VVMO6_02741:0.192813604784; VVMO6_02596:0.173411357825; VVMO6_02756:-0.116298587827; VVMO6_02732:0.167529552758; VVMO6_00281:-0.0227135659848; VVMO6_02733:0.103153527701; VVMO6_02742:0.263719912395; VVMO6_00598:-0.0125936272817; VVMO6_00517:-0.000153529975033; VVMO6_02746:0.339104246829; VVMO6_00331:0.271944932428; VVMO6_02751:0.285091574787; VVMO6_02506:-0.155451140553; VVMO6_02626:0.285482756267; VVMO6_00161:0.0722969825464; VVMO6_02752:0.14209785921; VVMO6_02755:0.017846838403; VVMO6_02754:0.174522276934; VVMO6_02743:-0.00753408001729; VVMO6_02740:0.248940828692; VVMO6_00163:0.00332012212812; VVMO6_00332:0.0267673373389; VVMO6_00162:-0.170667099766; VVMO6_00160:0.0262016603765; VVMO6_02597:-0.0821659784801; VVMO6_02745:-0.039089602876; VVMO6_02736:0.161230991463; VVMO6_02748:0.263211854787; VVMO6_02729:0.317450264286; VVMO6_02739:0.312070550027; VVMO6_00520:0.232863505782; VVMO6_01279:0.271334938278; VVMO6_02667:-0.0663929578233; VVMO6_02750:0.144333723001; VVMO6_02753:0.279091517665; VVMO6_02744:0.162730813172; VVMO6_01797:-0.0268557522788; VVMO6_02666:0.173562420905; VVMO6_02820:-0.113173843799; VVMO6_02747:0.374267582392; VVMO6_02737:0.521850739546; VVMO6_00728:0.431661117462; VVMO6_00302:-0.227578397191; VVMO6_01946:0.511785071535; VVMO6_02819:-0.00216175227973; VVMO6_02975:-0.00922244893926; VVMO6_01278:0.114586271625;
Bacterial secretion system	vvm03070	2.970147	VVMO6_02608:0.216004801705; VVMO6_02871:0.726913769728; VVMO6_02872:0.437040384587; VVMO6_02869:0.611733599585; VVMO6_02868:0.789129738567; VVMO6_02867:0.551327516087; VVMO6_02866:0.577995736104; VVMO6_02865:0.604040125919; VVMO6_02864:0.548441132384; VVMO6_02863:0.589429163561; VVMO6_02862:0.500049783235; VVMO6_02870:0.726917382803; VVMO6_04117:-1.32631106079; VVMO6_02446:0.198302883403; VVMO6_04116:-1.107955469; VVMO6_02445:0.285301736702; VVMO6_00158:-0.062374336995; VVMO6_00592:-0.107567294278; VVMO6_02735:0.00652853006468; VVMO6_02447:0.02505088288; VVMO6_02978:0.0155872692954; VVMO6_02568:0.306928376645; VVMO6_00128:-0.106281023168; VVMO6_00227:0.275374262647; VVMO6_00516:0.195342408715; VVMO6_02901:0.0358997586618; VVMO6_02900:0.144542339407; VVMO6_02899:-0.209203032364; VVMO6_03899:0.452548016122; VVMO6_03911:-0.0233439428239; VVMO6_03912:-0.0909277219252; VVMO6_03900:-0.0665288274479;
Aminoacyl-tRNA biosynthesis	vvm00970	2.5062403	VVMO6_02276:0.309471065466; VVMO6_02214:0.223263297803; VVMO6_00502:0.037903496854; VVMO6_02423:0.0563792356867; VVMO6_01023:0.170596890712; VVMO6_01128:0.118052159054; VVMO6_02961:0.2488281707; VVMO6_02962:0.320760187855; VVMO6_01276:-0.131119754275; VVMO6_01888:0.313662048464; VVMO6_01852:0.0322659931301; VVMO6_01958:0.204755506982; VVMO6_00037:0.0861337856622; VVMO6_00403:0.318918750013; VVMO6_02307:0.284046032834; VVMO6_02503:0.126957796671; VVMO6_02522:0.334237573643; VVMO6_02182:0.216443935562; VVMO6_00726:0.262570937653; VVMO6_02427:0.362076539945; VVMO6_01250:0.156435903101; VVMO6_01251:0.146364932023; VVMO6_04246:-0.173965847226; VVMO6_00580:-0.0847795343335; VVMO6_00254:0.201390114041;

beta-Lactam resistance	vvm01501	2.3591912	VVMO6_00056:0.848804891003; VVMO6_00057:0.738860151699; VVMO6_00058:0.390747848308; VVMO6_00318:0.155713326599; VVMO6_00583:0.345926062283; VVMO6_00704:0.0776522263922; VVMO6_02010:-0.342834971404; VVMO6_02011:-0.407104267786; VVMO6_02012:-0.116435945321; VVMO6_02013:-0.352892903301; VVMO6_02014:-0.102607202664; VVMO6_02312:0.109220980701; VVMO6_02492:0.142639318571; VVMO6_02583:0.0347633482936; VVMO6_02608:0.216004801705; VVMO6_03994:-0.390306903526; VVMO6_04162:2.13092477922;
Cationic antimicrobial peptide (CAMP) resistance	vvm01503	2.1551834	VVMO6_00027:0.900438144217; VVMO6_00056:0.848804891003; VVMO6_00057:0.738860151699; VVMO6_00203:0.082037918686; VVMO6_00204:-0.2443696828; VVMO6_00238:0.210270345237; VVMO6_00705:0.165908774053; VVMO6_00758:0.127525033461; VVMO6_01826:0.0720653300391; VVMO6_01827:-0.0700203962834; VVMO6_01828:-0.105472849954; VVMO6_01829:-0.144417548429; VVMO6_01830:-0.198485223468; VVMO6_02608:0.216004801705; VVMO6_03206:0.456746143066; VVMO6_03217:0.660484589218; VVMO6_03314:-0.265589657364; VVMO6_03994:-0.390306903526; VVMO6_04270:0.0160092611229;
Porphyrin and chlorophyll metabolism	vvm00860	2.0273442	VVMO6_02276:0.309471065466; VVMO6_02294:0.128781500759; VVMO6_00575:-0.112713829922; VVMO6_02896:0.304064798408; VVMO6_00098:0.127539825114; VVMO6_00097:0.29841177411; VVMO6_00096:0.32495992966; VVMO6_02721:-0.622404196003; VVMO6_03873:-0.116391164418; VVMO6_03844:0.653675905742; VVMO6_01662:-0.0778581268701; VVMO6_00170:0.176662085989; VVMO6_00046:0.276135805771; VVMO6_03382:1.53177073559; VVMO6_00427:-0.00380149891164; VVMO6_02888:-0.0486871730404; VVMO6_02950:-0.145971527956; VVMO6_02223:0.0400087130855; VVMO6_04019:-0.803896555883; VVMO6_04020:-0.702798589515; VVMO6_01954:-0.0555109917774; VVMO6_03809:0.125318101324; VVMO6_02558:0.110152845054; VVMO6_01741:0.195103028214; VVMO6_01742:0.18732140284; VVMO6_01743:-0.0734880600021; VVMO6_01740:0.207860292783; VVMO6_02912:-0.124419311152; VVMO6_00286:0.706380868457;
Purine metabolism	vvm00230	1.797877	VVMO6_02607:0.022796405402; VVMO6_03504:0.282328957943; VVMO6_02859:0.05442038487; VVMO6_00624:-0.967853841038; VVMO6_02207:0.421297396381; VVMO6_02297:0.297431624509; VVMO6_00879:0.0907968254545; VVMO6_00177:0.265252075824; VVMO6_00783:0.132488689671; VVMO6_01258:0.0976132278222; VVMO6_02374:0.402947382456; VVMO6_00784:0.0925593371144; VVMO6_00043:0.285469514665; VVMO6_00044:0.179907186389; VVMO6_01750:0.0772436558162; VVMO6_01872:0.019786013079; VVMO6_00178:0.20808435307; VVMO6_00885:0.102499626294; VVMO6_03733:-0.0972303807452; VVMO6_02288:0.255096365911; VVMO6_00494:0.0722805389708; VVMO6_01868:-0.0508413560636; VVMO6_03716:-0.0418269448314; VVMO6_00625:0.15770074692; VVMO6_03310:-0.726809050189; VVMO6_03164:-3.45716849859; VVMO6_00536:0.27629312593; VVMO6_02419:0.273068683764; VVMO6_02431:0.0388066376219; VVMO6_00428:0.350694566348; VVMO6_00486:0.152254954874; VVMO6_02366:-0.230366697336; VVMO6_02418:0.290541689382; VVMO6_04085:-0.0573663583076; VVMO6_02844:-0.135167589577; VVMO6_02638:0.390324015786; VVMO6_01929:-0.344497276304; VVMO6_03706:-0.484909087731; VVMO6_01974:0.278105192172; VVMO6_01975:0.347213635728; VVMO6_03816:0.543128172909; VVMO6_02115:0.115979530118; VVMO6_00921:0.398654412662; VVMO6_02730:0.136401320718; VVMO6_00164:0.0475578460289; VVMO6_00165:0.0842712099048; VVMO6_02845:0.232332194116; VVMO6_01585:-0.0778752885736; VVMO6_02895:0.29747528393; VVMO6_00761:-0.0421324087875; VVMO6_02969:-0.0906985718101; VVMO6_00886:0.023474616156; VVMO6_02309:0.109225127222; VVMO6_01936:-0.0375378925942;

Pyrimidine metabolism	vvm00240	1.741015	VVMO6_02564:-0.240490724053; VVMO6_02565:-0.420746372612; VVMO6_00392:-0.0103732706947; VVMO6_00391:0.0667603147145; VVMO6_03364:-0.136061180397; VVMO6_01539:-0.255671626475; VVMO6_02826:0.174251253068; VVMO6_01916:0.288092204592; VVMO6_01921:0.0221864797187; VVMO6_00748:0.26566689657; VVMO6_02431:0.0388066376219; VVMO6_00605:0.0569297024474; VVMO6_00487:0.137885925585; VVMO6_02730:0.136401320718; VVMO6_00164:0.0475578460289; VVMO6_00165:0.0842712099048; VVMO6_02845:0.232332194116; VVMO6_01585:-0.0778752885736; VVMO6_02895:0.29747528393; VVMO6_00761:-0.0421324087875; VVMO6_02969:-0.0906985718101; VVMO6_00886:0.023474616156; VVMO6_02309:0.109225127222; VVMO6_01936:-0.0375378925942; VVMO6_04309:-0.33991423535; VVMO6_04396:-0.290513938457; VVMO6_01118:-0.0317868395391; VVMO6_00772:0.148325633217; VVMO6_00404:0.00684584738169; VVMO6_00614:0.0600416458687; VVMO6_01956:0.262621275776; VVMO6_03733:-0.0972303807452; VVMO6_02288:0.255096365911; VVMO6_00494:0.0722805389708; VVMO6_03172:0.904899610945; VVMO6_00938:-1.12555325312; VVMO6_00785:0.149819485566; VVMO6_04159:-0.148737023668; VVMO6_00486:0.152254954874; VVMO6_01761:-0.197526698685; VVMO6_03816:0.543128172909; VVMO6_01974:0.278105192172; VVMO6_01975:0.347213635728; VVMO6_02511:0.423304922186; VVMO6_03564:0.514400058898; VVMO6_01259:-0.403116625287; VVMO6_00623:-1.17825220354; VVMO6_00625:0.15770074692; VVMO6_03310:-0.726809050189; VVMO6_02114:-0.0162127276304; VVMO6_03995:0.0139805368779; VVMO6_01851:-0.0662497406119; VVMO6_01937:0.0629410395363; VVMO6_01725:0.305130980343;
Amino sugar and nucleotide sugar metabolism	vvm00520	1.7125032	VVMO6_00721:-0.558806053767; VVMO6_03713:0.0349172579474; VVMO6_03025:0.116404721941; VVMO6_02492:0.142639318571; VVMO6_02282:-0.0629747874949; VVMO6_04050:0.00960302754319; VVMO6_00563:0.0560162027909; VVMO6_00562:0.526101417237; VVMO6_02250:0.235179238233; VVMO6_04531:-0.481519446378; VVMO6_04530:-0.632415403607; VVMO6_02215:0.0672605216933; VVMO6_01476:0.307319218333; VVMO6_00014:0.521445468249; VVMO6_04108:-0.736976513513; VVMO6_04107:-0.511496054202; VVMO6_02804:-0.0720887358255; VVMO6_04103:0.103695887107; VVMO6_02799:0.165662663209; VVMO6_02768:0.21753270602; VVMO6_02803:-0.0921465050593; VVMO6_02775:0.365591004243; VVMO6_02776:0.595365307371; VVMO6_02675:0.139127282633; VVMO6_00154:0.0211412220469; VVMO6_00564:-0.325927521745; VVMO6_00590:0.173406405404; VVMO6_04109:-0.310853011349; VVMO6_02216:0.208434881651; VVMO6_03007:0.243929546717; VVMO6_02636:0.110214720103; VVMO6_04330:-0.614535940735; VVMO6_01934:0.133973771111; VVMO6_00338:0.33013998615; VVMO6_02207:0.421297396381; VVMO6_00358:0.217941724634; VVMO6_02763:-0.19828596884; VVMO6_00665:0.376010623644; VVMO6_00664:0.308434476268; VVMO6_04486:0.457351214248; VVMO6_00663:0.19109240622; VVMO6_04487:0.312091343975; VVMO6_00287:0.466475359018; VVMO6_02770:0.415144419287; VVMO6_04028:0.32271754564; VVMO6_03149:0.150465956872; VVMO6_03979:-1.55886758688; VVMO6_03714:-1.24820620443; VVMO6_01000:0.148672179177;
Ubiquinone and other terpenoid-quinone biosynthesis	vvm00130	1.6940822	VVMO6_00136:0.0810243294317; VVMO6_00137:0.0153166887642; VVMO6_02914:-0.106539941931; VVMO6_02699:-0.0243811166821; VVMO6_00461:0.0499104621955; VVMO6_01972:-0.0288377449431; VVMO6_00460:0.0589765789637; VVMO6_02904:0.0343094110962; VVMO6_02300:-0.453576331495; VVMO6_02112:0.124298994563; VVMO6_04202:3.05735572503; VVMO6_02111:0.134015342242; VVMO6_02110:0.211958099839; VVMO6_02108:0.603426010008; VVMO6_02107:0.234197451504; VVMO6_02109:0.0218534054754; VVMO6_00296:-0.163151475758; VVMO6_00789:0.24833730927; VVMO6_01723:-2.12655239021;
Sphingolipid metabolism	vvm00600	1.6729867	VVMO6_03137:0.136849906351; VVMO6_03648:0.323004546985; VVMO6_03646:-0.321543448529; VVMO6_03138:0.460228544631; VVMO6_01835:0.90831350744;

Pentose phosphate pathway	vvm00030	1.5288301	VVMO6_00338:0.33013998615; VVMO6_01645:0.136406021755; VVMO6_01644:0.184836097855; VVMO6_01643:0.233376748379; VVMO6_00328:-0.165250872246; VVMO6_04014:0.451083589621; VVMO6_00445:0.348240697759; VVMO6_04013:-0.209035620012; VVMO6_00456:-0.0247630221512; VVMO6_00622:-0.683786681746; VVMO6_03514:-1.02607660368; VVMO6_00624:-0.967853841038; VVMO6_02207:0.421297396381; VVMO6_02297:0.297431624509; VVMO6_00076:0.544142474773; VVMO6_00079:0.416538497846; VVMO6_04464:0.0130335274186; VVMO6_04299:0.285697294591; VVMO6_04300:0.210812781695; VVMO6_00449:0.329542915402; VVMO6_02702:0.23303037994; VVMO6_00293:0.0522887508785; VVMO6_00207:0.0928598722246;
Pentose and glucuronate interconversions	vvm00040	1.509076	VVMO6_04289:0.277526785998; VVMO6_04484:0.513530148749; VVMO6_04306:0.746207370378; VVMO6_04462:0.0575574592484; VVMO6_04456:0.0614221084307; VVMO6_02763:-0.19828596884; VVMO6_00358:0.217941724634; VVMO6_04477:-0.0112856898387; VVMO6_00328:-0.165250872246; VVMO6_04237:0.0252030131885;
RNA degradation	vvm03018	1.3946436	VVMO6_01951:0.000576469270853; VVMO6_02918:0.0346824994772; VVMO6_00488:0.380850220231; VVMO6_00605:0.0569297024474; VVMO6_04068:-0.58927430357; VVMO6_03724:-0.157051876093; VVMO6_00251:-0.213091713919; VVMO6_03644:-0.191281463877; VVMO6_00719:-0.0347447856423; VVMO6_02922:0.215317978936; VVMO6_02916:0.00539558767041; VVMO6_00207:0.0928598722246; VVMO6_02385:0.342854896104; VVMO6_04522:0.640250265563; VVMO6_00210:0.716924360514; VVMO6_00241:-0.0714571992573; VVMO6_02467:-0.105701887969; VVMO6_00544:0.15130128063;
Starch and sucrose metabolism	vvm00500	1.3536457	VVMO6_01101:-0.197313338138; VVMO6_03115:0.108561971476; VVMO6_03098:0.219801108307; VVMO6_02326:-0.0429539348066; VVMO6_03032:0.0420125625756; VVMO6_02250:0.235179238233; VVMO6_02327:-0.578955916833; VVMO6_02763:-0.19828596884; VVMO6_00358:0.217941724634; VVMO6_02207:0.421297396381; VVMO6_00338:0.33013998615; VVMO6_03714:-1.24820620443; VVMO6_01000:0.148672179177; VVMO6_01001:0.479564399914; VVMO6_03058:0.346134068874; VVMO6_03056:0.428718248109; VVMO6_03389:0.0410853671074; VVMO6_03877:-0.204517202623; VVMO6_04288:0.168174416012; VVMO6_03748:0.148498010863; VVMO6_03057:0.237685540732;
Biosynthesis of antibiotics	vvm01130	1.3520833	VVMO6_00014:0.521445468249; VVMO6_00016:-0.313106587013; VVMO6_00019:-0.210599378602; VVMO6_00020:-0.117881197931; VVMO6_00021:-0.274753196726; VVMO6_00022:-0.18069945284; VVMO6_00023:-0.39321660794; VVMO6_00043:0.285469514665; VVMO6_00044:0.179907186389; VVMO6_00047:0.0304691753315; VVMO6_00054:-1.07139323806; VVMO6_00102:0.0367205511232; VVMO6_00103:0.266587994779; VVMO6_00116:0.00645309186003; VVMO6_00177:0.265252075824; VVMO6_00178:0.20808435307; VVMO6_00184:-0.516558431426; VVMO6_00185:-0.519987528371; VVMO6_00186:-0.623631907676; VVMO6_00187:-0.324470251611; VVMO6_00207:0.0928598722246; VVMO6_00216:0.044420794899; VVMO6_00217:0.0988513585825; VVMO6_00218:-0.121984046934; VVMO6_00219:-0.730012712159; VVMO6_00230:0.292310321201; VVMO6_00253:0.0181418025294; VVMO6_00258:-1.40052671174; VVMO6_00288:0.382526650771; VVMO6_00293:0.0522887508785; VVMO6_00305:-0.518206203142; VVMO6_00306:-0.292792875786; VVMO6_00311:-0.144905483357; VVMO6_00312:-2.09338949704; VVMO6_00313:-2.22892897895; VVMO6_00314:-1.30330925301; VVMO6_00315:-0.762774458134; VVMO6_00324:-0.188445634402; VVMO6_00325:-0.232873788178; VVMO6_00328:-0.165250872246; VVMO6_00338:0.33013998615; VVMO6_00354:-0.708493374183; VVMO6_00355:-0.325645270618; VVMO6_00358:0.217941724634; VVMO6_00389:-0.451675369534; VVMO6_00393:-2.07017705057; VVMO6_00394:-1.01191786361; VVMO6_00432:-0.0169030705607; VVMO6_00445:0.348240697759; VVMO6_00448:0.347239104814; VVMO6_00449:0.329542915402; VVMO6_00454:-0.0172981739191; VVMO6_00456:-0.0247630221512; VVMO6_00488:0.380850220231;

Glutathione metabolism	vvm00480	1.1965305	VVMO6_00511:0.491115094093; VVMO6_00438:0.199864381797; VVMO6_00405:0.156920267738; VVMO6_02432:-0.10972225889; VVMO6_01543:0.06999601024; VVMO6_02368:0.276388543127; VVMO6_04242:-0.416152178876; VVMO6_00082:0.0637307782807; VVMO6_00988:-0.0946671478574; VVMO6_01643:0.233376748379; VVMO6_01645:0.136406021755; VVMO6_04423:0.37202850744; VVMO6_03042:0.165326180118; VVMO6_03401:-0.621419697843;
Lipopolysaccharide biosynthesis	vvm00540	1.1286536	VVMO6_00758:0.127525033461; VVMO6_02571:-0.158085285584; VVMO6_00756:0.340874407693; VVMO6_01854:-0.10215194185; VVMO6_00759:-0.197297602767; VVMO6_00958:-0.179634245393; VVMO6_02683:-0.0781064208864; VVMO6_02289:0.145779466531; VVMO6_02684:-0.02476373427; VVMO6_00960:-0.0107775420778; VVMO6_02795:-0.0379827602215; VVMO6_02825:0.0153719595415; VVMO6_02793:0.172512642656; VVMO6_02810:0.235431511369; VVMO6_00781:0.0698420384298; VVMO6_02609:0.0788427499517; VVMO6_02329:-0.00778471124559; VVMO6_02792:0.0787524737903; VVMO6_02794:0.149127660489;
Glycolysis / Gluconeogenesis	vvm00010	1.062399	VVMO6_00338:0.33013998615; VVMO6_00207:0.0928598722246; VVMO6_02702:0.23303037994; VVMO6_00293:0.0522887508785; VVMO6_00449:0.329542915402; VVMO6_00288:0.382526650771; VVMO6_04135:-0.396169358599; VVMO6_02081:0.326479238617; VVMO6_00116:0.00645309186003; VVMO6_00448:0.347239104814; VVMO6_00230:0.292310321201; VVMO6_04533:0.0848804241509; VVMO6_00488:0.380850220231; VVMO6_02638:0.390324015786; VVMO6_01929:-0.344497276304; VVMO6_03706:-0.484909087731; VVMO6_00532:0.240433712386; VVMO6_00533:-0.104513012212; VVMO6_03940:-0.939969822472; VVMO6_00534:0.0638385104409; VVMO6_03234:-0.291465309358; VVMO6_02378:-2.07654428571; VVMO6_02043:0.497124943158; VVMO6_03472:0.313421195582; VVMO6_04237:0.0252030131885; VVMO6_00187:-0.324470251611; VVMO6_00666:0.269948098556; VVMO6_02207:0.421297396381; VVMO6_02082:0.291753994091; VVMO6_02875:-0.33367657265; VVMO6_04443:-0.2085457143; VVMO6_00413:-0.0106575824472; VVMO6_02250:0.235179238233; VVMO6_04330:-0.614535940735; VVMO6_01934:0.133973771111;
Glycerolipid metabolism	vvm00561	1.0348262	VVMO6_00552:0.0857642804803; VVMO6_04237:0.0252030131885; VVMO6_00678:-0.741752024858; VVMO6_00139:0.586858231593; VVMO6_01945:-0.234966053515; VVMO6_02623:-0.226397208806; VVMO6_02674:-0.0845502519043; VVMO6_01659:-0.135808718486; VVMO6_03207:0.180160019017; VVMO6_02811:0.111876706132; VVMO6_01226:-0.311848585314; VVMO6_03138:0.460228544631; VVMO6_01835:0.90831350744;
Vitamin B6 metabolism	vvm00750	0.9314297	VVMO6_04513:0.177655685305; VVMO6_03044:0.00872932004559; VVMO6_00447:0.064991063049; VVMO6_00871:0.127675458835; VVMO6_01765:0.177722245619; VVMO6_02658:0.140120979928; VVMO6_00477:0.179381450927; VVMO6_02540:-0.306866955636;
Peptidoglycan biosynthesis	vvm00550	0.8468578	VVMO6_02675:0.139127282633; VVMO6_00154:0.0211412220469; VVMO6_02576:-0.0416091506654; VVMO6_02579:-0.00458082561747; VVMO6_02582:-0.0230380527396; VVMO6_03772:-0.210828670995; VVMO6_02581:0.0385200603637; VVMO6_02620:0.0836570394175; VVMO6_02647:-0.0618870748988; VVMO6_02580:-0.0893571665161; VVMO6_02577:-0.0188162771626; VVMO6_02505:-0.157414393944; VVMO6_00318:0.155713326599; VVMO6_00549:-0.233214905761; VVMO6_02312:0.109220980701; VVMO6_02583:0.0347633482936; VVMO6_03447:-0.205536598632; VVMO6_02315:0.43938284824; VVMO6_00582:-0.0327995394882;
Fructose and mannose metabolism	vvm00051	0.7645313	VVMO6_03149:0.150465956872; VVMO6_03979:-1.55886758688; VVMO6_04028:0.32271754564; VVMO6_01069:0.626163066331; VVMO6_01070:0.238853840971; VVMO6_01072:-0.180090266719; VVMO6_00207:0.0928598722246; VVMO6_02702:0.23303037994; VVMO6_00293:0.0522887508785; VVMO6_02634:-0.347592838952; VVMO6_03698:0.166935878262; VVMO6_00449:0.329542915402; VVMO6_00288:0.382526650771; VVMO6_03699:-0.189998021801; VVMO6_03697:-0.132410790664; VVMO6_03153:0.442912688356; VVMO6_02633:-0.763576967599;

Taurine and hypotaurine metabolism	vvm00430	0.7006238	VVMO6_01774:-0.380969744731; VVMO6_03191:0.106223117246; VVMO6_01893:-0.188475577161; VVMO6_01541:-0.31670426028; VVMO6_01095:0.425639908581; VVMO6_03645:0.354363920671; VVMO6_01096:0.250033562257;
Cyanoamino acid metabolism	vvm00460	0.682672	VVMO6_03115:0.108561971476; VVMO6_03098:0.219801108307; VVMO6_02077:0.140470797603; VVMO6_02320:0.162030451039; VVMO6_03687:-0.340957742121;
Mismatch repair	vvm03430	0.639073	VVMO6_00498:0.0815110586735; VVMO6_00239:0.082357196434; VVMO6_02516:-0.15485172488; VVMO6_02926:-0.173355248544; VVMO6_01746:-0.178422849035; VVMO6_02420:-0.419520913604; VVMO6_02351:0.153643506624; VVMO6_02524:0.462604331404; VVMO6_00360:0.155935306531; VVMO6_00761:-0.0421324087875; VVMO6_02969:-0.0906985718101; VVMO6_00886:0.023474616156; VVMO6_02309:0.109225127222; VVMO6_01936:-0.0375378925942; VVMO6_04309:-0.33991423535; VVMO6_04396:-0.290513938457; VVMO6_01118:-0.0317868395391; VVMO6_00772:0.148325633217; VVMO6_00404:0.00684584738169; VVMO6_00614:0.0600416458687; VVMO6_02244:0.00485481642959; VVMO6_00327:-0.265073144015; VVMO6_01616:-0.106050309575;
Homologous recombination	vvm03440	0.6371926	VVMO6_02524:0.462604331404; VVMO6_00360:0.155935306531; VVMO6_02968:-0.228986949975; VVMO6_00476:0.0585740677481; VVMO6_00888:0.210126347813; VVMO6_00500:-0.2897002532; VVMO6_01585:-0.0778752885736; VVMO6_02895:0.29747528393; VVMO6_01026:-0.402096261234; VVMO6_01027:-0.427422482927; VVMO6_01024:-0.2454838635; VVMO6_02848:-0.228799774078; VVMO6_00687:-0.00680598713768; VVMO6_00686:-0.197798103024; VVMO6_00688:-0.0973915693194; VVMO6_00761:-0.0421324087875; VVMO6_02969:-0.0906985718101; VVMO6_00886:0.023474616156; VVMO6_02309:0.109225127222; VVMO6_01936:-0.0375378925942; VVMO6_04309:-0.33991423535; VVMO6_04396:-0.290513938457; VVMO6_01118:-0.0317868395391; VVMO6_00772:0.148325633217; VVMO6_00404:0.00684584738169; VVMO6_00614:0.0600416458687; VVMO6_00301:0.0355858017084; VVMO6_00982:0.245313055894;
Arginine and proline metabolism	vvm00330	0.5768488	VVMO6_04159:-0.148737023668; VVMO6_01237:0.233200031666; VVMO6_01236:0.259720971674; VVMO6_04423:0.37202850744; VVMO6_03042:0.165326180118; VVMO6_03401:-0.621419697843; VVMO6_01981:-0.633815955367; VVMO6_01980:-0.594483810194; VVMO6_04237:0.0252030131885; VVMO6_00259:-0.918400462837; VVMO6_00260:-0.702323082159; VVMO6_01738:-0.0803167428598; VVMO6_00432:-0.0169030705607; VVMO6_04509:0.939407409389; VVMO6_02363:0.412797244308; VVMO6_02362:0.370340732554; VVMO6_03813:0.378869920926;
Riboflavin metabolism	vvm00740	0.5499579	VVMO6_01108:-0.0376776106543; VVMO6_02358:0.116525064265; VVMO6_02360:0.0737384192936; VVMO6_03219:-0.457554762087; VVMO6_02357:-0.0196347986705; VVMO6_02359:0.194150142333; VVMO6_01466:0.253248416557; VVMO6_02504:-0.0798633857584; VVMO6_02912:-0.124419311152;
Glycine, serine and threonine metabolism	vvm00260	0.5464708	VVMO6_00503:-0.183373594909; VVMO6_00354:-0.708493374183; VVMO6_02542:-0.340150345427; VVMO6_00306:-0.292792875786; VVMO6_00872:0.106007057523; VVMO6_02047:0.157218179666; VVMO6_02541:-0.10309211092; VVMO6_02540:-0.306866955636; VVMO6_03503:0.192679673302; VVMO6_02320:0.162030451039; VVMO6_03687:-0.340957742121; VVMO6_00627:-0.205254738794; VVMO6_01765:0.177722245619; VVMO6_04368:-0.780872434043; VVMO6_00454:-0.0172981739191; VVMO6_00552:0.0857642804803; VVMO6_00230:0.292310321201; VVMO6_04533:0.0848804241509; VVMO6_00389:-0.451675369534; VVMO6_03258:1.46769946467; VVMO6_03259:1.3722224365; VVMO6_03685:0.237459208731; VVMO6_03690:-0.0301647417956; VVMO6_00534:0.0638385104409; VVMO6_03686:-0.286136419776; VVMO6_00355:-0.325645270618; VVMO6_00152:0.198706967391; VVMO6_03454:-0.364878129546; VVMO6_03453:-0.30952783635; VVMO6_03443:0.036465721437; VVMO6_01133:-0.112912063589; VVMO6_00019:-0.210599378602; VVMO6_02001:-0.354405150221; VVMO6_02000:-0.301809756874; VVMO6_01982:-0.674353356925;

DNA replication	vvm03030	0.514682	VVMO6_04309:-0.33991423535; VVMO6_04396:-0.290513938457; VVMO6_01118:-0.0317868395391; VVMO6_00772:0.148325633217; VVMO6_00761:-0.0421324087875; VVMO6_00886:0.023474616156; VVMO6_01936:-0.0375378925942; VVMO6_02309:0.109225127222; VVMO6_00614:0.0600416458687; VVMO6_00404:0.00684584738169; VVMO6_02969:-0.0906985718101; VVMO6_00334:-0.120056167984; VVMO6_02628:-0.267887024175; VVMO6_00360:0.155935306531; VVMO6_00771:-0.171144992482; VVMO6_00760:-0.161623002583; VVMO6_01585:-0.0778752885736; VVMO6_02895:0.29747528393; VVMO6_02244:0.00485481642959; VVMO6_01616:-0.106050309575;
Cysteine and methionine metabolism	vvm00270	0.442052	VVMO6_00225:0.0247181743555; VVMO6_02247:-0.246964264254; VVMO6_02096:0.925665543381; VVMO6_01817:0.0264087533588; VVMO6_04454:-0.258881207678; VVMO6_00353:0.00552922855703; VVMO6_04389:-0.934804172695; VVMO6_01094:-0.573188459462; VVMO6_00444:0.451663138412; VVMO6_03401:-0.621419697843; VVMO6_02557:0.251001007759; VVMO6_01122:0.239552326003; VVMO6_02062:-0.00249265601107; VVMO6_00513:0.180814745152; VVMO6_00503:-0.183373594909; VVMO6_00354:-0.708493374183; VVMO6_02542:-0.340150345427; VVMO6_00306:-0.292792875786; VVMO6_00872:0.106007057523; VVMO6_02047:0.157218179666; VVMO6_01726:-0.110255600366; VVMO6_00305:-0.518206203142; VVMO6_00021:-0.274753196726; VVMO6_00511:0.491115094093; VVMO6_00438:0.199864381797; VVMO6_03813:0.378869920926; VVMO6_04009:-0.0477617134218; VVMO6_03234:-0.291465309358; VVMO6_02669:-0.00814498133134; VVMO6_03443:0.036465721437; VVMO6_01133:-0.112912063589;
One carbon pool by folate	vvm00670	0.4185197	VVMO6_02662:-0.038309837926; VVMO6_03715:-0.189508656345; VVMO6_00904:-0.185740464617; VVMO6_02320:0.162030451039; VVMO6_03687:-0.340957742121; VVMO6_00783:0.132488689671; VVMO6_01258:0.0976132278222; VVMO6_00178:0.20808435307; VVMO6_00037:0.0861337856622; VVMO6_03690:-0.0301647417956; VVMO6_00353:0.00552922855703; VVMO6_02511:0.423304922186; VVMO6_03564:0.514400058898; VVMO6_02180:-0.145367739337; VVMO6_00307:-0.839029023614; VVMO6_00457:-0.697283278079;
Nicotinate and nicotinamide metabolism	vvm00760	0.3635155	VVMO6_00467:0.337634300172; VVMO6_01039:-0.357732635158; VVMO6_00528:0.0864175830263; VVMO6_01252:-0.173732395902; VVMO6_00625:0.15770074692; VVMO6_03310:-0.726809050189; VVMO6_03733:-0.0972303807452; VVMO6_02288:0.255096365911; VVMO6_00494:0.0722805389708; VVMO6_03367:-0.0537096943414; VVMO6_00167:0.0449098475668; VVMO6_03366:0.105724614305; VVMO6_00499:-0.226252908522; VVMO6_01254:-0.0982236381905; VVMO6_02393:-0.327857756806; VVMO6_00142:0.401439067709; VVMO6_03797:-0.235545070645; VVMO6_03796:-0.292922762383;
Nucleotide excision repair	vvm03420	0.3440535	VVMO6_00950:0.200054617598; VVMO6_00357:-0.250527880806; VVMO6_02024:-0.326184060355; VVMO6_01984:0.0106512763593; VVMO6_02926:-0.173355248544; VVMO6_01585:-0.0778752885736; VVMO6_02895:0.29747528393; VVMO6_02244:0.00485481642959; VVMO6_01616:-0.106050309575;
Protein export	vvm03060	0.3411999	VVMO6_02735:0.00652853006468; VVMO6_00158:-0.062374336995; VVMO6_00592:-0.107567294278; VVMO6_04117:-1.32631106079; VVMO6_02446:0.198302883403; VVMO6_04116:-1.107955469; VVMO6_02445:0.285301736702; VVMO6_02447:0.02505088288; VVMO6_02978:0.0155872692954; VVMO6_02568:0.306928376645; VVMO6_00227:0.275374262647; VVMO6_00516:0.195342408715; VVMO6_00128:-0.106281023168; VVMO6_02901:0.0358997586618; VVMO6_02900:0.144542339407; VVMO6_02899:-0.209203032364; VVMO6_00473:0.00314760934015; VVMO6_02502:0.246842386658;
Vancomycin resistance	vvm01502	0.320288	VVMO6_00335:-0.064501466824; VVMO6_02577:-0.0188162771626; VVMO6_02580:-0.0893571665161; VVMO6_02581:0.0385200603637; VVMO6_03772:-0.210828670995; VVMO6_03946:0.117342318115;

Sulfur metabolism	vvm00920	0.2836641	VVMO6_03191:0.106223117246; VVMO6_02719:-0.182820017668; VVMO6_02720:-0.265841430818; VVMO6_02717:-0.16487268043; VVMO6_02860:-0.0644556251315; VVMO6_00347:-0.0516868616398; VVMO6_00345:-0.424939985984; VVMO6_00346:0.0377752872183; VVMO6_04009:-0.0477617134218; VVMO6_00133:-0.0598000921792; VVMO6_00225:0.0247181743555; VVMO6_02247:-0.246964264254; VVMO6_02096:0.925665543381; VVMO6_01726:-0.110255600366; VVMO6_00305:-0.518206203142;
Terpenoid backbone biosynthesis	vvm00900	0.2833205	VVMO6_02353:0.0685299730123; VVMO6_00752:-0.29378813937; VVMO6_00491:0.183632643198; VVMO6_02296:0.0364556366119; VVMO6_00492:0.0103552437167; VVMO6_02428:0.0885674837447; VVMO6_02500:-0.0632906930615; VVMO6_03959:-1.12718380584; VVMO6_03614:0.18455912786; VVMO6_02352:-0.0103306704031; VVMO6_02668:0.167195022832; VVMO6_00750:-0.0229227233543;
Biosynthesis of secondary metabolites	vvm01110	0.18355	VVMO6_00016:-0.313106587013; VVMO6_00019:-0.210599378602; VVMO6_00020:-0.117881197931; VVMO6_00021:-0.274753196726; VVMO6_00022:-0.18069945284; VVMO6_00023:-0.39321660794; VVMO6_00043:0.285469514665; VVMO6_00044:0.179907186389; VVMO6_00046:0.276135805771; VVMO6_00047:0.0304691753315; VVMO6_00054:-1.07139323806; VVMO6_00096:0.32495992966; VVMO6_00097:0.29841177411; VVMO6_00098:0.127539825114; VVMO6_00102:0.0367205511232; VVMO6_00103:0.266587994779; VVMO6_00106:0.0436573284715; VVMO6_00116:0.00645309186003; VVMO6_00133:-0.0598000921792; VVMO6_00136:0.0810243294317; VVMO6_00137:0.0153166887642; VVMO6_00139:0.586858231593; VVMO6_00152:0.198706967391; VVMO6_00170:0.176662085989; VVMO6_00177:0.265252075824; VVMO6_00178:0.20808435307; VVMO6_00184:-0.516558431426; VVMO6_00185:-0.519987528371; VVMO6_00186:-0.623631907676; VVMO6_00187:-0.324470251611; VVMO6_00207:0.0928598722246; VVMO6_00216:0.044420794899; VVMO6_00217:0.0988513585825; VVMO6_00218:-0.121984046934; VVMO6_00219:-0.730012712159; VVMO6_00225:0.0247181743555; VVMO6_00226:0.145732824489; VVMO6_00230:0.292310321201; VVMO6_00233:0.0387752415905; VVMO6_00240:0.350836698616; VVMO6_00253:0.0181418025294; VVMO6_00258:-1.40052671174; VVMO6_00288:0.382526650771; VVMO6_00293:0.0522887508785; VVMO6_00296:-0.163151475758; VVMO6_00305:-0.518206203142; VVMO6_00306:-0.292792875786; VVMO6_00311:-0.144905483357; VVMO6_00312:-2.09338949704; VVMO6_00313:-2.22892897895; VVMO6_00314:-1.30330925301; VVMO6_00315:-0.762774458134; VVMO6_00324:-0.188445634402; VVMO6_00325:-0.232873788178;
Base excision repair	vvm03410	0.1289874	VVMO6_02817:-0.0372982187128; VVMO6_02032:-0.658810167327; VVMO6_00426:-0.235840127742; VVMO6_03015:-0.351467768315; VVMO6_01818:0.0297059623493; VVMO6_02537:-0.141006303183; VVMO6_00423:-0.373744712088; VVMO6_00979:0.1818717408; VVMO6_02538:-0.266370307991; VVMO6_01585:-0.0778752885736; VVMO6_02895:0.29747528393; VVMO6_02524:0.462604331404; VVMO6_02244:0.00485481642959; VVMO6_01616:-0.106050309575;
Phenylalanine, tyrosine and tryptophan biosynthesis	vvm00400	0.0867026	VVMO6_01613:0.409452649576; VVMO6_04200:3.177383162; VVMO6_04017:-0.517859880941; VVMO6_02491:0.0320731423063; VVMO6_00325:-0.232873788178; VVMO6_00186:-0.623631907676; VVMO6_00047:0.0304691753315; VVMO6_00324:-0.188445634402; VVMO6_00996:0.447407264135; VVMO6_00865:-0.111865693008; VVMO6_01996:-0.413036960727; VVMO6_01997:-0.251284173603; VVMO6_01998:-0.488032169404; VVMO6_01999:-0.397795742393; VVMO6_02001:-0.354405150221; VVMO6_02000:-0.301809756874; VVMO6_02490:-0.0588321665155; VVMO6_02483:-0.0539144660615; VVMO6_00016:-0.313106587013; VVMO6_03813:0.378869920926; VVMO6_01862:-0.55417914186; VVMO6_01122:0.239552326003; VVMO6_03925:-2.15099307232;

Flagellar assembly	vvm02040	-0.007209	VVMO6_00832:-0.0576042680574; VVMO6_00831:-0.319949781038; VVMO6_00830:-0.330279714285; VVMO6_00829:0.0396895307533; VVMO6_00828:-0.0450492943837; VVMO6_00827:0.141664218375; VVMO6_00826:0.234264936643; VVMO6_00825:-0.174089068643; VVMO6_02349:-0.0992040944211; VVMO6_02350:-0.228703667384; VVMO6_02263:-0.141990739163; VVMO6_02264:0.00290312341568; VVMO6_00823:-0.0457231503556; VVMO6_00822:-0.264990802477; VVMO6_00821:-0.298845720779; VVMO6_00820:-0.0599062387934; VVMO6_00819:0.0368777586045; VVMO6_00818:-0.0619441607068; VVMO6_00817:-0.0775652541382; VVMO6_02265:0.00258883641382; VVMO6_02266:-0.158116726916; VVMO6_02269:-0.0437006606936; VVMO6_02270:-0.157118760283; VVMO6_02271:-0.379873619252; VVMO6_02262:-0.115593063045; VVMO6_02261:-0.161836023726; VVMO6_02260:0.00693853091301; VVMO6_02259:-0.175082224766; VVMO6_02257:-0.264422165856; VVMO6_02256:-0.163195838818; VVMO6_00808:-0.0565719659161; VVMO6_00809:0.11257015321; VVMO6_02251:-0.160425762654; VVMO6_02252:-0.177519717478; VVMO6_02255:-0.00575003146984; VVMO6_00807:-0.304940642409; VVMO6_00811:-0.0537939724347; VVMO6_00813:-0.190484558356;
Monobactam biosynthesis	vvm00261	-0.087005	VVMO6_02719:-0.182820017668; VVMO6_02720:-0.265841430818; VVMO6_00503:-0.183373594909; VVMO6_00354:-0.708493374183; VVMO6_02542:-0.340150345427; VVMO6_00306:-0.292792875786; VVMO6_00872:0.106007057523; VVMO6_02047:0.157218179666; VVMO6_00796:0.20104470584; VVMO6_02566:0.260012163217;
Pyruvate metabolism	vvm00620	-0.114304	VVMO6_00187:-0.324470251611; VVMO6_00532:0.240433712386; VVMO6_00533:-0.104513012212; VVMO6_03940:-0.939969822472; VVMO6_00534:0.0638385104409; VVMO6_00971:0.878169246337; VVMO6_02043:0.497124943158; VVMO6_03645:0.354363920671; VVMO6_01096:0.250033562257; VVMO6_01095:0.425639908581; VVMO6_02638:0.390324015786; VVMO6_01929:-0.344497276304; VVMO6_03706:-0.484909087731; VVMO6_00762:-0.0959453171459; VVMO6_00185:-0.519987528371; VVMO6_00184:-0.516558431426; VVMO6_00875:-0.204075648355; VVMO6_04537:0.494250079958; VVMO6_04237:0.0252030131885; VVMO6_03234:-0.291465309358; VVMO6_01074:-0.13615531024; VVMO6_00897:0.252355685807; VVMO6_02033:-0.871588691715; VVMO6_00769:-0.0148213069792; VVMO6_01754:0.24227592302; VVMO6_00303:0.0392870897858; VVMO6_00506:-0.292418493431; VVMO6_00507:-0.178623597701; VVMO6_02669:-0.00814498133134; VVMO6_01140:-0.886647795614; VVMO6_00219:-0.730012712159; VVMO6_00218:-0.121984046934; VVMO6_00217:0.0988513585825; VVMO6_00216:0.044420794899; VVMO6_00310:0.0703881413244; VVMO6_02875:-0.33367657265; VVMO6_03459:0.157671634377; VVMO6_03414:-0.222120578215; VVMO6_02453:-0.133848970413; VVMO6_03959:-1.12718380584; VVMO6_02650:-0.544985161358;
Galactose metabolism	vvm00052	-0.12629	VVMO6_00666:0.269948098556; VVMO6_00665:0.376010623644; VVMO6_00664:0.308434476268; VVMO6_04486:0.457351214248; VVMO6_00663:0.19109240622; VVMO6_04487:0.312091343975; VVMO6_00287:0.466475359018; VVMO6_02770:0.415144419287; VVMO6_00358:0.217941724634; VVMO6_02207:0.421297396381; VVMO6_03137:0.136849906351; VVMO6_00660:-2.90542611548; VVMO6_00659:-4.35062924681; VVMO6_03138:0.460228544631; VVMO6_01835:0.90831350744; VVMO6_00207:0.0928598722246; VVMO6_01101:-0.197313338138; VVMO6_03748:0.148498010863;
Lysine biosynthesis	vvm00300	-0.13862	VVMO6_00503:-0.183373594909; VVMO6_00354:-0.708493374183; VVMO6_02542:-0.340150345427; VVMO6_00306:-0.292792875786; VVMO6_00872:0.106007057523; VVMO6_02047:0.157218179666; VVMO6_00796:0.20104470584; VVMO6_02566:0.260012163217; VVMO6_00680:-0.0829195112325; VVMO6_00258:-1.40052671174; VVMO6_00800:0.0231606950296; VVMO6_00103:0.266587994779; VVMO6_00102:0.0367205511232; VVMO6_02582:-0.0230380527396; VVMO6_02581:0.0385200603637;

Pantothenate and CoA biosynthesis	vvm00770	-0.255323	VVMO6_02643:-0.323550360544; VVMO6_00023:-0.39321660794; VVMO6_02642:-0.143941821051; VVMO6_00022:-0.18069945284; VVMO6_00054:-1.07139323806; VVMO6_00020:-0.117881197931; VVMO6_00542:0.0631541906117; VVMO6_00698:0.221718761723; VVMO6_00541:-0.000484446978405; VVMO6_00156:-0.283528708557; VVMO6_02823:0.111844045626; VVMO6_02815:0.250051762516; VVMO6_00523:0.308807583533; VVMO6_00478:0.151454527993; VVMO6_03723:-0.635621054839; VVMO6_00021:-0.274753196726;
Sulfur relay system	vvm04122	-0.313859	VVMO6_02439:0.27193031058; VVMO6_04009:-0.0477617134218; VVMO6_04007:-0.0989803320086; VVMO6_02958:0.171072920807; VVMO6_00280:0.0419511893696; VVMO6_00279:0.329639912261; VVMO6_00278:0.315886436268; VVMO6_01669:0.0556518737775; VVMO6_01870:-0.0187232304045; VVMO6_02016:-0.64964358061; VVMO6_02020:-0.761111595368; VVMO6_02017:-0.826998473768; VVMO6_02018:-0.393150510061; VVMO6_02348:-0.0863518950832; VVMO6_02940:-0.618418759875;
Folate biosynthesis	vvm00790	-0.376751	VVMO6_04005:-0.0752115269554; VVMO6_00899:-0.00897481909261; VVMO6_03603:0.305083331162; VVMO6_02622:0.031426983238; VVMO6_02621:-0.166487173861; VVMO6_00543:-0.0770315025134; VVMO6_00589:-0.0125644573212; VVMO6_02662:-0.038309837926; VVMO6_00876:-0.0547815995032; VVMO6_01130:-0.354220246288; VVMO6_01679:-0.0966627398718; VVMO6_01678:-0.145609785923; VVMO6_02335:0.298266100474; VVMO6_02020:-0.761111595368; VVMO6_02018:-0.393150510061; VVMO6_02016:-0.64964358061; VVMO6_02017:-0.826998473768; VVMO6_00257:-0.182148475809; VVMO6_01139:0.141679875736; VVMO6_01939:-0.0554629568336;
Biotin metabolism	vvm00780	-0.673916	VVMO6_01884:0.197844656987; VVMO6_00870:-0.579456111746; VVMO6_02158:0.0235404430788; VVMO6_02161:0.117932497813; VVMO6_01940:-0.389517389596; VVMO6_03953:-1.03966334362; VVMO6_02160:0.213699522971; VVMO6_01942:-0.0878682745723; VVMO6_00757:-0.086765379826; VVMO6_02855:-0.116988566038; VVMO6_01885:-0.665593004539; VVMO6_01887:0.0509115402167; VVMO6_01883:0.0690070579304; VVMO6_01886:-0.821649230246; VVMO6_00155:0.0332023013526;
Citrate cycle (TCA cycle)	vvm00020	-0.736749	VVMO6_02200:-0.221494925341; VVMO6_01688:-0.533230492234; VVMO6_00551:-0.282708542955; VVMO6_00988:-0.0946671478574; VVMO6_02195:0.2554334795; VVMO6_02194:0.309864212692; VVMO6_00534:0.0638385104409; VVMO6_02192:-0.152767538577; VVMO6_02193:-0.0796698284078; VVMO6_02197:-0.451367180227; VVMO6_02196:0.274132396248; VVMO6_02198:-0.660775121208; VVMO6_00219:-0.730012712159; VVMO6_00218:-0.121984046934; VVMO6_00217:0.0988513585825; VVMO6_00216:0.044420794899; VVMO6_01140:-0.886647795614; VVMO6_02669:-0.00814498133134; VVMO6_02875:-0.33367657265; VVMO6_00532:0.240433712386; VVMO6_00533:-0.104513012212; VVMO6_03940:-0.939969822472;
Nitrogen metabolism	vvm00910	-0.824676	VVMO6_04096:-0.340455205688; VVMO6_04097:-0.380274541004; VVMO6_03847:0.660887713474; VVMO6_03866:-0.105680268834; VVMO6_03846:0.887950564928; VVMO6_01967:-0.779463494813; VVMO6_01810:-0.00864772892412; VVMO6_01541:-0.31670426028; VVMO6_02883:-0.425161575988; VVMO6_02552:-0.553162744157; VVMO6_02554:-0.449150256222; VVMO6_02553:-0.680597099531; VVMO6_02555:-0.313680988748; VVMO6_00537:-0.550623984302; VVMO6_03293:-0.0474803615539;
Selenocompound metabolism	vvm00450	-0.918712	VVMO6_00305:-0.518206203142; VVMO6_01817:0.0264087533588; VVMO6_04454:-0.258881207678; VVMO6_00353:0.00552922855703; VVMO6_04389:-0.934804172695; VVMO6_01094:-0.573188459462; VVMO6_01761:-0.197526698685; VVMO6_02719:-0.182820017668; VVMO6_02720:-0.265841430818; VVMO6_01958:0.204755506982;
Lysine degradation	vvm00310	-0.924221	VVMO6_02195:0.2554334795; VVMO6_02194:0.309864212692; VVMO6_02953:-2.18589874316; VVMO6_00859:0.0221768082564; VVMO6_03959:-1.12718380584; VVMO6_00934:0.361099265743; VVMO6_03071:0.0243213142523; VVMO6_04237:0.0252030131885;

Propanoate metabolism	vvm00640	-0.944127	VVMO6_00187:-0.324470251611; VVMO6_01686:0.19282293011; VVMO6_03645:0.354363920671; VVMO6_01096:0.250033562257; VVMO6_01095:0.425639908581; VVMO6_00534:0.0638385104409; VVMO6_00971:0.878169246337; VVMO6_02953:-2.18589874316; VVMO6_00859:0.0221768082564; VVMO6_00762:-0.0959453171459; VVMO6_00185:-0.519987528371; VVMO6_00184:-0.516558431426; VVMO6_00875:-0.204075648355; VVMO6_04537:0.494250079958; VVMO6_02193:-0.0796698284078; VVMO6_02192:-0.152767538577; VVMO6_03958:-1.39363652698; VVMO6_01689:-0.220904698883; VVMO6_00551:-0.282708542955; VVMO6_01690:-0.272012878768; VVMO6_03234:-0.291465309358; VVMO6_03959:-1.12718380584; VVMO6_03619:0.393783165715; VVMO6_03711:-0.584809126962;
Degradation of aromatic compounds	vvm01220	-0.960994	VVMO6_00289:-0.439529798668; VVMO6_02043:0.497124943158; VVMO6_02378:-2.07654428571; VVMO6_02699:-0.0243811166821; VVMO6_03472:0.313421195582;
Histidine metabolism	vvm00340	-1.044313	VVMO6_01864:-0.765034655174; VVMO6_01857:-0.315303617014; VVMO6_01859:-0.190142366995; VVMO6_01860:-0.35647649514; VVMO6_01858:0.00544010597498; VVMO6_01861:-0.299669610848; VVMO6_01862:-0.55417914186; VVMO6_01863:-0.80108267224; VVMO6_01269:-0.100733485201; VVMO6_02153:0.303819706635; VVMO6_01270:-0.440601226395; VVMO6_01272:-0.570549487331; VVMO6_01271:-0.24149050004; VVMO6_03218:0.186338865463; VVMO6_04237:0.0252030131885; VVMO6_04147:-0.182913116674; VVMO6_00782:0.0645048950898;
Oxidative phosphorylation	vvm00190	-1.084347	VVMO6_00947:0.481700279938; VVMO6_02197:-0.451367180227; VVMO6_02196:0.274132396248; VVMO6_02198:-0.660775121208; VVMO6_00219:-0.730012712159; VVMO6_00218:-0.121984046934; VVMO6_00217:0.0988513585825; VVMO6_00216:0.044420794899; VVMO6_02595:-0.953991509491; VVMO6_02594:-0.781843838654; VVMO6_02593:-0.677729382299; VVMO6_04019:-0.803896555883; VVMO6_04026:-0.0661141096374; VVMO6_04020:-0.702798589515; VVMO6_01523:-0.695855607296; VVMO6_01522:-0.611287342785; VVMO6_01521:-0.750308421863; VVMO6_01520:-0.708829694877; VVMO6_01029:0.363766210667; VVMO6_01030:0.272857987139; VVMO6_00010:0.18689255087; VVMO6_00012:0.237533689044; VVMO6_00011:0.138887426055; VVMO6_00009:-0.0265650454228; VVMO6_00013:0.248912057189; VVMO6_00006:0.0245982037357; VVMO6_00008:0.151991464964; VVMO6_00007:0.220235957015; VVMO6_02703:-0.110115272636; VVMO6_01833:-0.507256186801; VVMO6_02467:-0.105701887969;
Fatty acid biosynthesis	vvm00061	-1.136868	VVMO6_00762:-0.0959453171459; VVMO6_00185:-0.519987528371; VVMO6_00184:-0.516558431426; VVMO6_00875:-0.204075648355; VVMO6_04537:0.494250079958; VVMO6_01943:-0.368501278057; VVMO6_00735:-0.14051318518; VVMO6_03827:0.179248638773; VVMO6_01944:-0.465255199853; VVMO6_00870:-0.579456111746; VVMO6_02158:0.0235404430788; VVMO6_02161:0.117932497813; VVMO6_01940:-0.389517389596; VVMO6_03953:-1.03966334362; VVMO6_02160:0.213699522971; VVMO6_01942:-0.0878682745723; VVMO6_01532:-0.86480945959; VVMO6_00757:-0.086765379826; VVMO6_01780:-0.556449064791; VVMO6_03758:-0.495004528108; VVMO6_02644:-0.0805920384183; VVMO6_02173:0.116266890115;

Microbial metabolism in diverse environments	vvm01120	-1.157933	VVMO6_00076:0.544142474773; VVMO6_00079:0.416538497846; VVMO6_00096:0.32495992966; VVMO6_00097:0.29841177411; VVMO6_00098:0.127539825114; VVMO6_00102:0.0367205511232; VVMO6_00103:0.266587994779; VVMO6_00116:0.00645309186003; VVMO6_00133:-0.0598000921792; VVMO6_00184:-0.516558431426; VVMO6_00185:-0.519987528371; VVMO6_00187:-0.324470251611; VVMO6_00207:0.0928598722246; VVMO6_00216:0.044420794899; VVMO6_00217:0.0988513585825; VVMO6_00218:-0.121984046934; VVMO6_00219:-0.730012712159; VVMO6_00225:0.0247181743555; VVMO6_00230:0.292310321201; VVMO6_00258:-1.40052671174; VVMO6_00263:-0.408938917528; VVMO6_00288:0.382526650771; VVMO6_00289:-0.439529798668; VVMO6_00293:0.0522887508785; VVMO6_00303:0.0392870897858; VVMO6_00306:-0.292792875786; VVMO6_00307:-0.839029023614; VVMO6_00310:0.0703881413244; VVMO6_00328:-0.165250872246; VVMO6_00338:0.33013998615; VVMO6_00345:-0.424939985984; VVMO6_00346:0.0377752872183; VVMO6_00347:-0.0516868616398; VVMO6_00354:-0.708493374183; VVMO6_00355:-0.325645270618; VVMO6_00389:-0.451675369534; VVMO6_00445:0.348240697759; VVMO6_00448:0.347239104814; VVMO6_00449:0.329542915402; VVMO6_00454:-0.0172981739191; VVMO6_00456:-0.0247630221512; VVMO6_00488:0.380850220231; VVMO6_00503:-0.183373594909; VVMO6_00532:0.240433712386; VVMO6_00533:-0.104513012212; VVMO6_00534:0.0638385104409; VVMO6_00551:-0.282708542955; VVMO6_00552:0.0857642804803; VVMO6_00575:-0.112713829922; VVMO6_00627:-0.205254738794; VVMO6_00666:0.269948098556; VVMO6_00680:-0.0829195112325; VVMO6_00762:-0.0959453171459; VVMO6_00796:0.20104470584;
Thiamine metabolism	vvm00730	-1.192487	VVMO6_02942:-0.850017964851; VVMO6_03225:-0.627482968596; VVMO6_02940:-0.618418759875; VVMO6_02439:0.27193031058; VVMO6_02348:-0.0863518950832; VVMO6_02937:-0.39016064005; VVMO6_02353:0.0685299730123; VVMO6_02938:-0.530611098892; VVMO6_02941:-0.249198972446; VVMO6_02355:-0.158573487745;
Alanine, aspartate and glutamate metabolism	vvm00250	-1.195575	VVMO6_03813:0.378869920926; VVMO6_00467:0.337634300172; VVMO6_02077:0.140470797603; VVMO6_02219:-1.19459046612; VVMO6_02113:-0.135151539913; VVMO6_00355:-0.325645270618; VVMO6_01893:-0.188475577161; VVMO6_00199:0.151140307364; VVMO6_00314:-1.30330925301; VVMO6_00315:-0.762774458134; VVMO6_04138:-1.23806431026; VVMO6_00246:0.0835568745678; VVMO6_01872:0.019786013079; VVMO6_00392:-0.0103732706947; VVMO6_00391:0.0667603147145; VVMO6_01774:-0.380969744731; VVMO6_03071:0.0243213142523; VVMO6_02552:-0.553162744157; VVMO6_02554:-0.449150256222; VVMO6_02553:-0.680597099531; VVMO6_02555:-0.313680988748; VVMO6_01541:-0.31670426028; VVMO6_04509:0.939407409389; VVMO6_02883:-0.425161575988; VVMO6_02564:-0.240490724053; VVMO6_02565:-0.420746372612; VVMO6_00425:-0.126328639805; VVMO6_02636:0.110214720103; VVMO6_00879:0.0907968254545;
Phosphotransferase system (PTS)	vvm02060	-1.304978	VVMO6_02249:0.156160044055; VVMO6_02248:-0.0291919292063; VVMO6_02250:0.235179238233; VVMO6_04330:-0.614535940735; VVMO6_01934:0.133973771111; VVMO6_02215:0.0672605216933; VVMO6_02327:-0.578955916833; VVMO6_04531:-0.481519446378; VVMO6_00411:-0.232725152162; VVMO6_02633:-0.763576967599; VVMO6_04228:-1.39747813741; VVMO6_04229:-1.20125222683; VVMO6_04230:-0.463269996834; VVMO6_03699:-0.189998021801; VVMO6_03697:-0.132410790664; VVMO6_03153:0.442912688356; VVMO6_02514:0.0151308363228; VVMO6_02692:-0.0912512278165; VVMO6_03152:-0.0866573610355;

Metabolic pathways	vvm01100	-1.319313	VVMO6_00006:0.0245982037357; VVMO6_00007:0.220235957015; VVMO6_00008:0.151991464964; VVMO6_00009:-0.0265650454228; VVMO6_00010:0.18689255087; VVMO6_00011:0.138887426055; VVMO6_00012:0.237533689044; VVMO6_00013:0.248912057189; VVMO6_00014:0.521445468249; VVMO6_00016:-0.313106587013; VVMO6_00019:-0.210599378602; VVMO6_00020:-0.117881197931; VVMO6_00021:-0.274753196726; VVMO6_00022:-0.18069945284; VVMO6_00023:-0.39321660794; VVMO6_00043:0.285469514665; VVMO6_00044:0.179907186389; VVMO6_00046:0.276135805771; VVMO6_00047:0.0304691753315; VVMO6_00054:-1.07139323806; VVMO6_00076:0.544142474773; VVMO6_00079:0.416538497846; VVMO6_00096:0.32495992966; VVMO6_00097:0.29841177411; VVMO6_00098:0.127539825114; VVMO6_00102:0.0367205511232; VVMO6_00103:0.266587994779; VVMO6_00106:0.0436573284715; VVMO6_00116:0.00645309186003; VVMO6_00136:0.0810243294317; VVMO6_00137:0.0153166887642; VVMO6_00139:0.586858231593; VVMO6_00142:0.401439067709; VVMO6_00148:-0.115003801411; VVMO6_00152:0.198706967391; VVMO6_00154:0.0211412220469; VVMO6_00155:0.0332023013526; VVMO6_00156:-0.283528708557; VVMO6_00164:0.0475578460289; VVMO6_00165:0.0842712099048; VVMO6_00167:0.0449098475668; VVMO6_00170:0.176662085989; VVMO6_00177:0.265252075824; VVMO6_00178:0.20808435307; VVMO6_00184:-0.516558431426; VVMO6_00185:-0.519987528371; VVMO6_00186:-0.623631907676; VVMO6_00187:-0.324470251611; VVMO6_00199:0.151140307364; VVMO6_00207:0.0928598722246; VVMO6_00216:0.044420794899; VVMO6_00217:0.0988513585825; VVMO6_00218:-0.121984046934; VVMO6_00219:-0.730012712159;
C5-Branched dibasic acid metabolism	vvm00660	-1.361574	VVMO6_02643:-0.323550360544; VVMO6_00023:-0.39321660794; VVMO6_02642:-0.143941821051; VVMO6_00022:-0.18069945284; VVMO6_02193:-0.0796698284078; VVMO6_02192:-0.152767538577; VVMO6_02652:-0.699708328654; VVMO6_02653:-0.791530945479; VVMO6_02651:-0.467893727833;
Carbon metabolism	vvm01200	-1.440622	VVMO6_00019:-0.210599378602; VVMO6_00076:0.544142474773; VVMO6_00079:0.416538497846; VVMO6_00116:0.00645309186003; VVMO6_00184:-0.516558431426; VVMO6_00185:-0.519987528371; VVMO6_00187:-0.324470251611; VVMO6_00207:0.0928598722246; VVMO6_00216:0.044420794899; VVMO6_00217:0.0988513585825; VVMO6_00218:-0.121984046934; VVMO6_00219:-0.730012712159; VVMO6_00225:0.0247181743555; VVMO6_00230:0.292310321201; VVMO6_00263:-0.408938917528; VVMO6_00288:0.382526650771; VVMO6_00293:0.0522887508785; VVMO6_00303:0.0392870897858; VVMO6_00307:-0.839029023614; VVMO6_00310:0.0703881413244; VVMO6_00328:-0.165250872246; VVMO6_00338:0.33013998615; VVMO6_00355:-0.325645270618; VVMO6_00389:-0.451675369534; VVMO6_00445:0.348240697759; VVMO6_00448:0.347239104814; VVMO6_00449:0.329542915402; VVMO6_00454:-0.0172981739191; VVMO6_00456:-0.0247630221512; VVMO6_00488:0.380850220231; VVMO6_00532:0.240433712386; VVMO6_00533:-0.104513012212; VVMO6_00534:0.0638385104409; VVMO6_00551:-0.282708542955; VVMO6_00627:-0.205254738794; VVMO6_00762:-0.0959453171459; VVMO6_00859:0.0221768082564; VVMO6_00875:-0.204075648355; VVMO6_00904:-0.185740464617; VVMO6_00988:-0.0946671478574; VVMO6_01095:0.425639908581; VVMO6_01096:0.250033562257; VVMO6_01133:-0.112912063589; VVMO6_01140:-0.886647795614; VVMO6_01495:-1.93529267062; VVMO6_01496:-1.86603791273; VVMO6_01497:-1.82173127814; VVMO6_01643:0.233376748379; VVMO6_01644:0.184836097855; VVMO6_01645:0.136406021755; VVMO6_01754:0.24227592302; VVMO6_01765:0.177722245619; VVMO6_01780:-0.556449064791; VVMO6_01929:-0.344497276304;
Biosynthesis of unsaturated fatty acids	vvm01040	-1.725006	VVMO6_03953:-1.03966334362; VVMO6_02160:0.213699522971; VVMO6_01942:-0.0878682745723; VVMO6_03518:-0.559061023114; VVMO6_02953:-2.18589874316; VVMO6_00859:0.0221768082564; VVMO6_03759:-0.142838908056; VVMO6_02093:0.147204446735; VVMO6_02005:-0.199909104068;

Two-component system	vvm02020	-1.866438	VVMO6_02470:-0.0294969480898; VVMO6_02471:-0.170896313239; VVMO6_00899:-0.00897481909261; VVMO6_03292:0.0573614984539; VVMO6_03325:-1.36168118668; VVMO6_03640:-0.576958376652; VVMO6_02469:-0.118862387017; VVMO6_02850:0.0830962564173; VVMO6_02851:-0.236688620484; VVMO6_03208:0.0937225196232; VVMO6_00891:-0.185154087719; VVMO6_00892:-0.0383595801659; VVMO6_03209:0.413772125205; VVMO6_00203:0.082037918686; VVMO6_00204:-0.2443696828; VVMO6_04385:1.3666947856; VVMO6_04384:1.28097598552; VVMO6_04219:0.479523543072; VVMO6_04220:-0.0939777183012; VVMO6_03083:0.148398114506; VVMO6_00808:-0.0565719659161; VVMO6_00809:0.11257015321; VVMO6_02251:-0.160425762654; VVMO6_02252:-0.177519717478; VVMO6_02255:-0.00575003146984; VVMO6_00807:-0.304940642409; VVMO6_00835:-0.284288599408; VVMO6_02350:-0.228703667384; VVMO6_03621:-0.106206388188; VVMO6_01008:0.262962325765; VVMO6_01840:-1.07436736427; VVMO6_01839:-0.593005354435; VVMO6_01007:-0.401908486772; VVMO6_02548:-0.155148722248; VVMO6_02546:0.101075753131; VVMO6_01693:-0.852296516129; VVMO6_04020:-0.702798589515; VVMO6_02970:-0.120422026342; VVMO6_03601:-0.404479057948; VVMO6_03835:0.371267113275; VVMO6_01754:0.24227592302; VVMO6_04093:0.164788237252; VVMO6_04092:0.0806469595301; VVMO6_00219:-0.730012712159; VVMO6_00218:-0.121984046934; VVMO6_00217:0.0988513585825; VVMO6_00216:0.044420794899; VVMO6_03978:-1.12187145839; VVMO6_03707:-0.194824754711; VVMO6_03977:0.122490965933; VVMO6_03976:0.161720595975; VVMO6_03975:-0.932457360333; VVMO6_00483:0.212100351241; VVMO6_01985:-0.00351552190426;
Butanoate metabolism	vvm00650	-1.910994	VVMO6_03959:-1.12718380584; VVMO6_02953:-2.18589874316; VVMO6_00859:0.0221768082564; VVMO6_01780:-0.556449064791; VVMO6_03758:-0.495004528108; VVMO6_02043:0.497124943158; VVMO6_04112:0.126009537129; VVMO6_02197:-0.451367180227; VVMO6_02196:0.274132396248; VVMO6_02198:-0.660775121208; VVMO6_00219:-0.730012712159; VVMO6_00218:-0.121984046934; VVMO6_00217:0.0988513585825; VVMO6_00216:0.044420794899; VVMO6_01774:-0.380969744731; VVMO6_03071:0.0243213142523; VVMO6_00971:0.878169246337; VVMO6_03964:-0.777709029766; VVMO6_03926:-0.998403356745; VVMO6_02643:-0.323550360544; VVMO6_00023:-0.39321660794; VVMO6_02642:-0.143941821051; VVMO6_00022:-0.18069945284;
Valine, leucine and isoleucine biosynthesis	vvm00290	-1.919345	VVMO6_00019:-0.210599378602; VVMO6_02652:-0.699708328654; VVMO6_02653:-0.791530945479; VVMO6_02651:-0.467893727833; VVMO6_02643:-0.323550360544; VVMO6_00023:-0.39321660794; VVMO6_02642:-0.143941821051; VVMO6_00022:-0.18069945284; VVMO6_00054:-1.07139323806; VVMO6_00020:-0.117881197931; VVMO6_00021:-0.274753196726; VVMO6_02964:-0.128542650666; VVMO6_02113:-0.135151539913; VVMO6_02650:-0.544985161358;

Biosynthesis of amino acids	vvm01230	-1.993091	VVMO6_00016:-0.313106587013; VVMO6_00019:-0.210599378602; VVMO6_00020:-0.117881197931; VVMO6_00021:-0.274753196726; VVMO6_00022:-0.18069945284; VVMO6_00023:-0.39321660794; VVMO6_00047:0.0304691753315; VVMO6_00054:-1.07139323806; VVMO6_00102:0.0367205511232; VVMO6_00103:0.266587994779; VVMO6_00116:0.00645309186003; VVMO6_00186:-0.623631907676; VVMO6_00207:0.0928598722246; VVMO6_00225:0.0247181743555; VVMO6_00230:0.292310321201; VVMO6_00258:-1.40052671174; VVMO6_00288:0.382526650771; VVMO6_00305:-0.518206203142; VVMO6_00306:-0.292792875786; VVMO6_00311:-0.144905483357; VVMO6_00312:-2.09338949704; VVMO6_00313:-2.22892897895; VVMO6_00314:-1.30330925301; VVMO6_00315:-0.762774458134; VVMO6_00324:-0.188445634402; VVMO6_00325:-0.232873788178; VVMO6_00328:-0.165250872246; VVMO6_00353:0.00552922855703; VVMO6_00354:-0.708493374183; VVMO6_00393:-2.07017705057; VVMO6_00432:-0.0169030705607; VVMO6_00444:0.451663138412; VVMO6_00445:0.348240697759; VVMO6_00448:0.347239104814; VVMO6_00449:0.329542915402; VVMO6_00454:-0.0172981739191; VVMO6_00456:-0.0247630221512; VVMO6_00488:0.380850220231; VVMO6_00503:-0.183373594909; VVMO6_00513:0.180814745152; VVMO6_00551:-0.282708542955; VVMO6_00627:-0.205254738794; VVMO6_00680:-0.0829195112325; VVMO6_00690:-1.02103179; VVMO6_00796:0.20104470584; VVMO6_00800:0.0231606950296; VVMO6_00865:-0.111865693008; VVMO6_00872:0.106007057523; VVMO6_00988:-0.0946671478574; VVMO6_00996:0.447407264135; VVMO6_01094:-0.573188459462; VVMO6_01122:0.239552326003; VVMO6_01133:-0.112912063589; VVMO6_01613:0.409452649576;
ABC transporters	vvm02010	-2.122538	VVMO6_01482:-1.18480181392; VVMO6_01481:-1.36468030861; VVMO6_01480:-1.13918252185; VVMO6_03808:-0.188688976702; VVMO6_03807:-0.117755028783; VVMO6_03806:-0.345819510536; VVMO6_00558:0.468110993384; VVMO6_03435:-0.0442521250013; VVMO6_00559:0.0635731692671; VVMO6_00560:-0.105594905413; VVMO6_02698:-0.196751730943; VVMO6_02697:-0.171080788757; VVMO6_02696:-0.0675418051947; VVMO6_01505:-0.0580616846774; VVMO6_01506:-0.0783594186344; VVMO6_03235:-0.0230304740669; VVMO6_01507:0.0653892114722; VVMO6_01508:0.222063333741; VVMO6_01509:0.238670352968; VVMO6_03457:-0.368144238092; VVMO6_03354:0.313528832222; VVMO6_03036:0.0893857602642; VVMO6_03355:0.190993379657; VVMO6_03356:-0.397209341167; VVMO6_03352:0.0640242914041; VVMO6_03099:0.536678512683; VVMO6_02132:0.350784957402; VVMO6_04434:-0.559582140934; VVMO6_02678:0.0689567737377; VVMO6_02679:0.0254052431066; VVMO6_02680:0.218815523433; VVMO6_02677:0.106640760595; VVMO6_02681:0.342290654845; VVMO6_02135:-0.0225909873791; VVMO6_02133:-0.688771928107; VVMO6_03513:-1.60676693104; VVMO6_03512:-2.64236250983; VVMO6_03511:-3.69079806797; VVMO6_03510:-4.63413295339; VVMO6_03135:-2.65474482366; VVMO6_03133:-0.439961194183; VVMO6_03134:-1.65883564935; VVMO6_01706:0.248653026211; VVMO6_03292:0.0573614984539; VVMO6_03325:-1.36168118668; VVMO6_03640:-0.576958376652; VVMO6_02469:-0.118862387017; VVMO6_02466:-0.282509320391; VVMO6_02465:-0.0285577578742; VVMO6_03327:-0.6263339143; VVMO6_02464:0.167224918887; VVMO6_03328:-0.39591479969; VVMO6_04185:-1.17402032662; VVMO6_04187:-0.770829949177;
Ascorbate and aldarate metabolism	vvm00053	-2.179766	VVMO6_02763:-0.19828596884; VVMO6_04228:-1.39747813741; VVMO6_04229:-1.20125222683; VVMO6_04230:-0.463269996834; VVMO6_04470:-0.357970097012; VVMO6_04237:0.0252030131885;

Bacterial chemotaxis	vvm02030	-2.190641	VVMO6_03390:0.0893640524068; VVMO6_01704:-0.158010752503; VVMO6_02611:-0.295432127713; VVMO6_03392:-0.263589139958; VVMO6_03492:-0.234381229795; VVMO6_00667:-0.458016592233; VVMO6_03296:0.00803554922283; VVMO6_03324:-0.339315569284; VVMO6_03336:0.0718308775109; VVMO6_04193:0.0875404269816; VVMO6_03992:-0.318345219207; VVMO6_04016:0.0445523967021; VVMO6_04435:-0.283259131031; VVMO6_04554:-0.128741141634; VVMO6_02222:-0.362773685011; VVMO6_03945:0.0646284706677; VVMO6_01473:-0.440838544567; VVMO6_01234:-0.00355298186151; VVMO6_00420:-0.91512557417; VVMO6_02139:-0.0380929385476; VVMO6_03001:-1.32863141758; VVMO6_03848:-0.0315669716735; VVMO6_03878:-0.335289074232; VVMO6_03879:-0.616983709324; VVMO6_03059:-0.613552308504; VVMO6_03068:-0.262170997295; VVMO6_03721:0.0909560703659; VVMO6_03814:0.0817685167715; VVMO6_01065:-0.257913413924; VVMO6_01117:0.29101911233; VVMO6_01129:-0.479714196467; VVMO6_02822:-0.657125678102; VVMO6_00928:0.131554530656; VVMO6_00940:-0.912051222966; VVMO6_03521:-0.528516936416; VVMO6_00180:-0.207329983183; VVMO6_00980:-0.623246531046; VVMO6_00969:-1.356849677; VVMO6_01814:-0.679958742576; VVMO6_01671:0.0550759932599; VVMO6_01681:-0.210328904254; VVMO6_03354:0.313528832222; VVMO6_03036:0.0893857602642; VVMO6_03513:-1.60676693104; VVMO6_03135:-2.65474482366; VVMO6_00838:-0.116435287365; VVMO6_04557:-0.248688108919; VVMO6_00841:-0.0851814856083; VVMO6_00842:0.267448633401; VVMO6_00836:-0.1884232706; VVMO6_00837:-0.272527984158; VVMO6_00339:0.0148800049441; VVMO6_00839:-0.165266515937; VVMO6_04551:0.0149580816074;
Glyoxylate and dicarboxylate metabolism	vvm00630	-2.249821	VVMO6_02452:-0.152373351227; VVMO6_03414:-0.222120578215; VVMO6_02453:-0.133848970413; VVMO6_02669:-0.00814498133134; VVMO6_02200:-0.221494925341; VVMO6_01688:-0.533230492234; VVMO6_00551:-0.282708542955; VVMO6_03959:-1.12718380584; VVMO6_03249:-0.554519265864; VVMO6_00253:0.0181418025294; VVMO6_00355:-0.325645270618; VVMO6_02883:-0.425161575988; VVMO6_02320:0.162030451039; VVMO6_03687:-0.340957742121; VVMO6_03685:0.237459208731; VVMO6_03690:-0.0301647417956; VVMO6_00534:0.0638385104409; VVMO6_03686:-0.286136419776; VVMO6_00552:0.0857642804803; VVMO6_00389:-0.451675369534; VVMO6_00079:0.416538497846; VVMO6_04464:0.0130335274186; VVMO6_04299:0.285697294591; VVMO6_01495:-1.93529267062; VVMO6_03161:-0.035519283938; VVMO6_01496:-1.86603791273; VVMO6_01497:-1.82173127814; VVMO6_02180:-0.145367739337;
Methane metabolism	vvm00680	-2.349875	VVMO6_02378:-2.07654428571; VVMO6_02379:-2.78193456477; VVMO6_01495:-1.93529267062; VVMO6_03161:-0.035519283938; VVMO6_01496:-1.86603791273; VVMO6_01497:-1.82173127814; VVMO6_02320:0.162030451039; VVMO6_03687:-0.340957742121; VVMO6_00355:-0.325645270618; VVMO6_00389:-0.451675369534; VVMO6_00488:0.380850220231; VVMO6_00310:0.0703881413244; VVMO6_02669:-0.00814498133134; VVMO6_00449:0.329542915402; VVMO6_02702:0.23303037994; VVMO6_00293:0.0522887508785; VVMO6_00207:0.0928598722246; VVMO6_03645:0.354363920671; VVMO6_01096:0.250033562257; VVMO6_01095:0.425639908581; VVMO6_00187:-0.324470251611; VVMO6_03459:0.157671634377; VVMO6_00230:0.292310321201; VVMO6_04533:0.0848804241509; VVMO6_04368:-0.780872434043; VVMO6_00454:-0.0172981739191; VVMO6_01765:0.177722245619; VVMO6_00627:-0.205254738794;
beta-Alanine metabolism	vvm00410	-2.590411	VVMO6_01774:-0.380969744731; VVMO6_03401:-0.621419697843; VVMO6_04237:0.0252030131885; VVMO6_00541:-0.000484446978405; VVMO6_02953:-2.18589874316; VVMO6_00859:0.0221768082564; VVMO6_03958:-1.39363652698;
Tyrosine metabolism	vvm00350	-2.967606	VVMO6_03813:0.378869920926; VVMO6_01862:-0.55417914186; VVMO6_01122:0.239552326003; VVMO6_01723:-2.12655239021; VVMO6_01722:-1.56541906619; VVMO6_01720:-1.30783563598; VVMO6_02378:-2.07654428571; VVMO6_02043:0.497124943158; VVMO6_03472:0.313421195582; VVMO6_00289:-0.439529798668; VVMO6_03071:0.0243213142523;

Tryptophan metabolism	vvm00380	-2.979652	VVMO6_02195:0.2554334795; VVMO6_02953:-2.18589874316; VVMO6_00859:0.0221768082564; VVMO6_03959:-1.12718380584; VVMO6_04222:-1.21612210808; VVMO6_04237:0.0252030131885; VVMO6_03249:-0.554519265864; VVMO6_01712:-0.729001248079;
Glycerophospholipid metabolism	vvm00564	-3.014861	VVMO6_00226:0.145732824489; VVMO6_03464:-3.18804463853; VVMO6_00676:-1.34349673187; VVMO6_03465:-2.12295327003; VVMO6_03466:-2.00167955248; VVMO6_00139:0.586858231593; VVMO6_01945:-0.234966053515; VVMO6_02623:-0.226397208806; VVMO6_02674:-0.0845502519043; VVMO6_01659:-0.135808718486; VVMO6_03207:0.180160019017; VVMO6_02811:0.111876706132; VVMO6_01752:-0.327672643846; VVMO6_00112:-0.114657122055; VVMO6_01711:0.182269357941; VVMO6_03883:0.0594637026938; VVMO6_00751:-0.426698192576; VVMO6_00152:0.198706967391; VVMO6_00233:0.0387752415905; VVMO6_01983:-0.327247150864; VVMO6_02354:-0.143979799513; VVMO6_03224:-0.564725156666; VVMO6_01816:-0.550347329028; VVMO6_02069:-0.462613541063;
Fatty acid metabolism	vvm01212	-3.178812	VVMO6_00184:-0.516558431426; VVMO6_00185:-0.519987528371; VVMO6_00735:-0.14051318518; VVMO6_00757:-0.086765379826; VVMO6_00762:-0.0959453171459; VVMO6_00780:-1.19161940553; VVMO6_00858:-0.0811930062781; VVMO6_00859:0.0221768082564; VVMO6_00870:-0.579456111746; VVMO6_00875:-0.204075648355; VVMO6_01532:-0.86480945959; VVMO6_01780:-0.556449064791; VVMO6_01940:-0.389517389596; VVMO6_01942:-0.0878682745723; VVMO6_01943:-0.368501278057; VVMO6_01944:-0.465255199853; VVMO6_02158:0.0235404430788; VVMO6_02160:0.213699522971; VVMO6_02161:0.117932497813; VVMO6_02173:0.116266890115; VVMO6_02644:-0.0805920384183; VVMO6_02953:-2.18589874316; VVMO6_02954:-2.12292112562; VVMO6_03518:-0.559061023114; VVMO6_03758:-0.495004528108; VVMO6_03827:0.179248638773; VVMO6_03953:-1.03966334362; VVMO6_03959:-1.12718380584; VVMO6_04537:0.494250079958;
Phenylalanine metabolism	vvm00360	-3.370365	VVMO6_03925:-2.15099307232; VVMO6_03813:0.378869920926; VVMO6_01862:-0.55417914186; VVMO6_01122:0.239552326003; VVMO6_01723:-2.12655239021; VVMO6_02414:-0.726773591278; VVMO6_01712:-0.729001248079;
Fatty acid degradation	vvm00071	-3.453325	VVMO6_03959:-1.12718380584; VVMO6_02954:-2.12292112562; VVMO6_00858:-0.0811930062781; VVMO6_02953:-2.18589874316; VVMO6_00859:0.0221768082564; VVMO6_00780:-1.19161940553; VVMO6_02644:-0.0805920384183; VVMO6_02173:0.116266890115; VVMO6_02378:-2.07654428571; VVMO6_02043:0.497124943158; VVMO6_03472:0.313421195582; VVMO6_04237:0.0252030131885;
2-Oxocarboxylic acid metabolism	vvm01210	-3.97631	VVMO6_00020:-0.117881197931; VVMO6_00021:-0.274753196726; VVMO6_00022:-0.18069945284; VVMO6_00023:-0.39321660794; VVMO6_00054:-1.07139323806; VVMO6_00258:-1.40052671174; VVMO6_00311:-0.144905483357; VVMO6_00312:-2.09338949704; VVMO6_00313:-2.22892897895; VVMO6_00315:-0.762774458134; VVMO6_00354:-0.708493374183; VVMO6_00503:-0.183373594909; VVMO6_00551:-0.282708542955; VVMO6_00690:-1.02103179; VVMO6_00872:0.106007057523; VVMO6_00988:-0.0946671478574; VVMO6_01982:-0.674353356925; VVMO6_02047:0.157218179666; VVMO6_02113:-0.135151539913; VVMO6_02200:-0.221494925341; VVMO6_02642:-0.143941821051; VVMO6_02643:-0.323550360544; VVMO6_02650:-0.544985161358; VVMO6_02651:-0.467893727833; VVMO6_02652:-0.699708328654; VVMO6_02653:-0.791530945479; VVMO6_03813:0.378869920926;
Benzoate degradation	vvm00362	-4.024929	VVMO6_02954:-2.12292112562; VVMO6_00858:-0.0811930062781; VVMO6_00859:0.0221768082564; VVMO6_02953:-2.18589874316; VVMO6_03959:-1.12718380584;
Geraniol degradation	vvm00281	-4.221809	VVMO6_03964:-0.777709029766; VVMO6_02954:-2.12292112562; VVMO6_00858:-0.0811930062781; VVMO6_03957:-1.19637555893; VVMO6_02953:-2.18589874316; VVMO6_00859:0.0221768082564;
Other glycan degradation	vvm00511	-4.717351	VVMO6_03137:0.136849906351; VVMO6_00660:-2.90542611548; VVMO6_00659:-4.35062924681; VVMO6_02282:-0.0629747874949; VVMO6_04050:0.00960302754319; VVMO6_00563:0.0560162027909; VVMO6_03162:-0.511024247168;

Arginine biosynthesis	vvm00220	-5.409955	VVMO6_00393:-2.07017705057; VVMO6_00314:-1.30330925301; VVMO6_00315:-0.762774458134; VVMO6_00394:-1.01191786361; VVMO6_00425:-0.126328639805; VVMO6_02883:-0.425161575988; VVMO6_01541:-0.31670426028; VVMO6_03813:0.378869920926; VVMO6_02113:-0.135151539913; VVMO6_00690:-1.02103179; VVMO6_00313:-2.22892897895; VVMO6_00312:-2.09338949704; VVMO6_00258:-1.40052671174; VVMO6_00311:-0.144905483357;
Valine, leucine and isoleucine degradation	vvm00280	-5.947856	VVMO6_00021:-0.274753196726; VVMO6_00534:0.0638385104409; VVMO6_03961:-2.85776273305; VVMO6_02953:-2.18589874316; VVMO6_00859:0.0221768082564; VVMO6_02954:-2.12292112562; VVMO6_00858:-0.0811930062781; VVMO6_03954:-1.52985531291; VVMO6_03403:0.0907527214765; VVMO6_03958:-1.39363652698; VVMO6_04237:0.0252030131885; VVMO6_03962:-1.15921474412; VVMO6_03964:-0.777709029766; VVMO6_03926:-0.998403356745; VVMO6_03959:-1.12718380584;
