# Supplementary material for: MARTX Toxin-Stimulated Interplay between Human Cells and Vibrio vulnificus
Source: mSphere. 2020 Aug 12;5(4):e00659-20. doi: 10.1128/mSphere.00659-20 (PMC7426173; doi:10.1128/mSphere.00659-20)
Supplement: TABLE S5 [file mSphere.00659-20-st005.pdf]

Table S5. Differentially regulated secretion system genes in *V. vulnificus* (WT vs. *Δ rtxA1*) during dTHP-1 cell infection

gene	Expression (log_2 CPM)						Fold change	protein_description
	WT_6h_1st	WT_6h_2nd	WT_6h_3rd	ΔrtxA1_6h_1st	ΔrtxA1_6h_2nd	ΔrtxA1_6h_3rd		
**VVMO6_00128**	7.576286028	7.551007763	7.446145518	7.65978989	7.657091153	7.574767356	0.9289797	signal recognition particle-docking protein FtsY, partial
**VVMO6_00158**	9.124044882	9.07819786	9.159167079	9.125730652	9.246850916	9.173628413	0.957686694	preprotein translocase subunit SecE
**VVMO6_00227**	8.441012572	8.460128574	8.548388356	8.129256375	8.191178347	8.302037083	1.210308026	protein-export protein SecB
**VVMO6_00516**	8.427023577	8.499486713	8.508116738	8.266205455	8.31018233	8.274848993	1.144995881	signal recognition particle protein
**VVMO6_00592**	10.28762197	10.52906009	10.43054714	10.73171406	10.3437154	10.47785979	0.928151814	preprotein translocase subunit SecG
**VVMO6_02445**	8.702387389	8.647470734	8.756644892	8.49540265	8.275116684	8.475902927	1.218665118	preprotein translocase subunit SecF
**VVMO6_02446**	8.865710919	8.702939515	8.780310973	8.597309676	8.486247317	8.670885243	1.147347876	preprotein translocase subunit SecD
**VVMO6_02447**	6.749114371	6.758517962	6.916409644	6.853532206	6.675894535	6.826557515	1.017515579	preprotein translocase subunit YajC
**VVMO6_02568**	8.684206288	8.593300717	8.742828388	8.456759079	8.221364415	8.418704004	1.237071062	preprotein translocase subunit SecA
**VVMO6_02608**	9.076046403	9.087312598	9.118990499	8.868129222	8.797719752	8.965559643	1.161512598	membrane protein
**VVMO6_02735**	12.6285219	12.68232428	12.80110431	12.61674638	12.78962844	12.68613012	1.004535487	preprotein translocase subunit SecY
**VVMO6_02862**	5.782135395	5.751238545	5.569829434	5.141642846	5.227308457	5.263368147	1.414262364	general secretion pathway protein GspM
**VVMO6_02863**	7.569226903	7.428662497	7.176122211	6.644597814	6.741243286	7.027820774	1.504651279	type II secretion system protein GspL
**VVMO6_02864**	6.860864138	6.776308511	6.59312237	6.128646772	6.209931453	6.267742304	1.46250457	general secretion pathway protein GspK
**VVMO6_02865**	6.704709899	6.521716468	6.177026028	5.951827408	5.758788183	5.947287288	1.519967131	type II secretion system protein GspJ
**VVMO6_02866**	4.918886912	4.74019904	4.583564694	4.129989335	4.119061075	4.335143291	1.492773971	type II secretion system protein GspI
**VVMO6_02867**	6.169598128	6.173952521	6.040186578	5.515906908	5.702831211	5.521850801	1.465433515	type II secretion system protein GspH
**VVMO6_02868**	8.119155519	8.041315125	7.951284685	7.075776951	7.200153684	7.455941922	1.728031766	type II secretion system protein GspG
**VVMO6_02869**	8.211819717	8.064305969	7.779215466	7.139100937	7.624330141	7.451431021	1.528094325	type II secretion system protein GspF
**VVMO6_02870**	8.612194469	8.390507973	8.141077998	7.483216786	7.661501474	7.840167896	1.655098849	type II secretion system protein GspE
**VVMO6_02871**	9.255190243	9.183948677	9.018010238	8.22523071	8.551463437	8.491915872	1.655094704	type II secretion system protein GspD
**VVMO6_02872**	8.147869341	8.143548657	7.983435248	7.413526244	7.798624033	7.729950589	1.353824175	type II secretion system protein GspC
**VVMO6_02899**	5.901602	6.050724373	6.228089914	6.176995033	6.506461583	6.09630843	0.865014947	preprotein translocase subunit TatC
**VVMO6_02900**	6.836376206	6.877029089	6.925072359	6.873981749	6.60286573	6.729296493	1.105379942	sec-independent translocase
**VVMO6_02901**	5.537897692	5.783264667	5.756132088	5.669383042	5.872214371	5.400732352	1.025196003	protein translocase TatA
**VVMO6_02978**	10.43294615	10.34036229	10.24783119	10.3534725	10.15421488	10.45663364	1.010862849	membrane protein insertase YidC
**VVMO6_03899**	2.182650666	2.83036948	2.621313149	1.976311152	2.384839194	2.189903884	1.368455021	hemolysin D
**VVMO6_03900**	2.9108883	2.909886152	3.291394364	2.818266138	3.430909937	2.947556778	0.954932837	ATPase
**VVMO6_03911**	2.740370936	2.577889315	2.856854896	2.741204882	2.801944505	2.696250979	0.983949418	type VI secretion protein IcmF
**VVMO6_04116**	3.671005749	3.747026262	3.544725939	4.634924975	4.637795157	4.821136036	0.463951059	preprotein translocase subunit SecF
