# Supplementary material for: MARTX Toxin-Stimulated Interplay between Human Cells and Vibrio vulnificus
Source: mSphere. 2020 Aug 12;5(4):e00659-20. doi: 10.1128/mSphere.00659-20 (PMC7426173; doi:10.1128/mSphere.00659-20)
Supplement: TABLE S6 [file mSphere.00659-20-st006.pdf]

Table S6. Differentially regulated host and *V. vulnificus* genes during infection and the oligonucleotides used in RT-qPCR

(A) Differentially regulated host and *V. vulnificus* genes during infection

Function	Gene	Expression (log_2 CPM)									Coding protein
		Mock_6h_1st	Mock_6h_2nd	Mock_6h_3rd	WT_6h_1st	WT_6h_2nd	WT_6h_3rd	ΔrtxA1_6h_1st	ΔrtxA1_6h_2nd	ΔrtxA1_6h_3rd	
**Genes expressed differentially in HT-29 cells upon *V. vulnificus* infection**											
**pro-inflammatory transcription factor**	**JUN**	5.569393526	5.691898904	5.714891989	9.027221548	9.004820154	8.890885345	6.717195504	7.022804299	6.723482009	transcription factor AP-1
	**FOS**	3.702103979	3.914471043	3.911611636	6.715675564	6.827829548	6.764275053	5.499056991	6.093611978	5.775183716	proto-oncogene c-Fos
	**ATF3**	2.225133438	2.120371957	2.541504811	5.280536535	5.401256972	5.485596905	3.508901833	3.392293861	3.72615041	cyclic AMP-dependent transcription factor ATF-3
**regulation of inflammatory responses, NF-κB signaling, and MAPK signaling**	**KLF2**	0.282390363	0.379029738	0.502547068	1.709817458	1.812104956	1.541272336	0.510748364	0.517840451	0.612434229	Krueppel-like factor 2
	**KLF4**	3.820376449	3.954965055	3.96758085	4.539061639	4.505059128	4.668303214	4.142509154	3.984178261	4.129258463	Krueppel-like factor 4
	**KLF6**	6.291692038	6.34993256	6.290395156	7.745716791	7.623510764	7.555830102	6.42872214	6.254573495	6.318893629	Krueppel-like factor 6
	**NLRP3**	1.930059121	1.938464258	2.115599425	0.838318796	0.9684802	0.720899192	2.073654877	2.223542499	2.024054496	NACHT, LRP, and PYD domains-containing protein 3
	**TLR4**	2.176676034	2.076999832	2.115599425	1.257328778	1.419194634	1.436062218	1.728025216	1.40886028	2.011494405	Toll-like receptor 4
	**CXCR4**	4.530758853	4.368707139	4.590828426	3.847537108	3.984594941	3.938175725	4.33023631	4.730573728	4.483170786	C-X-C chemokine receptor type 4
**anti-inflammation**	**TSC22D3**	3.725873478	3.96292923	3.732597029	5.13588846	5.2416861	5.119982775	4.552538305	4.924247489	4.52574684	glucocorticoid-induced leucine zipper, GILZ
**pro-inflammation**	**EDN1**	4.970937628	4.720716	5.016696973	3.721404141	3.519134378	4.09904203	4.169248636	4.073224487	4.60730534	endothelin-1
**Genes expressed differentially in dTHP-1 cells upon *V. vulnificus* infection**											
**pro-inflammatory cytokines/ chemokines**	**CSF2**	0	0	0.072808687	6.710394658	6.531792954	6.888596656	4.343981324	4.589041145	5.359090309	Granulocyte-macrophage colony-stimulating factor
	**IL6**	0	0	0.0368636	4.068971222	4.06906891	4.567950981	1.377822014	2.209675968	2.628594969	Interleukin-6
	**IL12B**	0	0	0.0368636	3.526360531	4.037166636	4.597262681	0.895976823	1.177036156	1.051441844	Interleukin-12 subunit beta
**inhibition of inflammation**	**PYCARD**	4.916940609	4.974688676	4.906259844	4.424986768	4.29270409	4.297320678	5.134521748	4.665711725	5.071459036	apoptosis-associated speck-like protein containing a CARD
**regulation of inflammatory responses and NF-κB signaling**	**TLR8**	2.76295483	2.587051647	2.726004876	4.373604231	4.100280965	4.787291132	2.8985026	3.257778803	3.011009755	Toll-like receptor 8
	**TLR9**	0.920307808	1.314381141	0.697015425	2.002299228	2.105597615	2.029717039	1.116579538	1.27426615	1.432042008	Toll-like receptor 9
	**CXCR4**	4.318986899	4.294700422	4.331000679	6.233044471	6.014086764	6.184400809	4.403877173	4.371904902	4.609898982	C-X-C chemokine receptor type 4
	**STAT4**	3.413517337	3.056130894	3.485062586	4.498784151	4.71185085	5.1391533	3.309779741	3.988292104	3.667803444	signal transducer and activator of transcription 4
	**IKBKB**	6.072896163	6.192914338	5.984040725	7.182952799	7.133088006	7.282154414	6.510096777	6.42924768	6.42632456	inhibitor of nuclear factor (NF)-κB kinase (IKK) subunit beta
	**IRF7**	3.748587289	3.973890512	3.825124642	4.450007684	4.592535957	4.527918677	3.866852475	4.142189932	4.343891253	Interferon regulatory factor 7
	**IRAK3**	4.991201168	4.756048391	4.913711553	4.019683967	3.99350233	4.40069143	4.497179695	4.668695394	4.375298063	interleukin-1 receptor-associated kinase 3
**anti-inflammation**	**TSC22D3**	4.11117414	4.101050524	4.030999997	3.617524722	3.586468514	2.90516747	3.236373552	3.192938859	2.805020898	glucocorticoid-induced leucine zipper, GILZ
**pro-inflammation**	**EDN1**	1.961225224	1.743224414	1.904349902	5.472637548	5.815252349	5.884961116	4.875150968	5.48280797	5.365360347	endothelin-1
**iron homeostasis**	**HAMP**	1.130947586	1.359080945	1.277070169	1.428189939	1.679201563	1.559400892	1.569346004	0.751125589	1.568729531	Hepcidin
	**SLC11A1**	7.222790339	7.312251372	7.073627623	7.449613681	7.34661901	7.415664812	7.14847792	7.07281261	6.997129669	natural resistance-associated macrophage protein 1
	**FTH1**	11.76440563	12.09174701	11.53144051	11.76998101	11.60040823	11.46788937	12.09271853	11.21390434	11.82232512	heavy chain of ferritin
	**FTL**	13.0450111	12.98163839	13.09906756	12.56124584	12.76282906	12.77520554	12.88709866	13.22079675	12.96786311	Light chain of ferritin
**Genes expressed differentially in *V. vulnificus* during dTHP-1 cell infection**											
**siderophore utilization**	**VVMO6_04197**		N.A. *		6.599155311	6.67846971	6.964124165	3.266208147	5.085717127	4.59442199	4\'-phosphopantetheinyl transferase
	**VVMO6_04198**		N.A.		9.932046386	9.878552322	10.16774713	5.420270507	8.051981315	7.275039015	peptide synthetase
	**VVMO6_04199**		N.A.		11.32216389	11.29719572	11.67357834	6.511702821	9.343339043	8.603870312	hypothetical protein
	**VVMO6_04200**		N.A.		8.547575973	8.615460503	9.034115854	4.189214466	6.31070993	5.568421015	3-deoxy-7-phosphoheptulonate synthase
	**VVMO6_04201**		N.A.		7.065350385	7.053727297	7.319359994	2.85530621	5.440771606	4.855685357	2,3-dihydro-2,3-dihydroxybenzoate dehydrogenase
	**VVMO6_04202**		N.A.		7.710024607	7.728269811	8.185016746	3.090950061	5.611567171	4.935376673	isochorismate synthase
	**VVMO6_04203**		N.A.		7.699071788	7.555135815	8.031693885	2.961041034	5.523859103	4.954630201	2,3-dihydroxybenzoate-AMP ligase
	**VVMO6_04205**		N.A.		9.625401751	9.576786185	9.69651471	5.533153799	8.005323239	7.419453512	NADPH-dependent ferric siderophore reductase
	**VVMO6_04206**		N.A.		9.29531447	9.394993292	9.659573877	5.521678811	8.083547648	7.32521178	isochorismatase
	**VVMO6_04207**		N.A.		9.135082171	9.065231164	9.32734882	5.126571598	7.597728352	7.074036392	isochorismate-pyruvate lyase
	**VVMO6_04208**		N.A.		9.643326316	9.579955165	9.838453058	5.456881089	7.791264041	7.617674497	2,3-dihydroxybenzoate-AMP ligase
	**VVMO6_04209**		N.A.		7.28203554	7.556680794	7.769628077	3.181238756	6.255376971	5.617566988	acyl carrier protein

	**VVMO6_04210**	N.A.	9.268108218	9.435303827	9.559964779	5.544538236	7.983178679	7.482715088	enterochelin ABC transporter substrate-binding protein
	**VVMO6_04211**	N.A.	12.24862841	12.55164534	12.9298531	7.733760915	11.03862465	10.01065986	ligand-gated channel protein
	**VVMO6_04212**	N.A.	7.87253262	7.772374643	8.033030179	4.852055788	6.557836518	6.161280516	short-chain isoprenyl diphosphate synthase
	**VVMO6_04403**	N.A.	6.731516246	6.546809497	6.65605106	4.676911992	5.825813927	6.12208187	(2Fe-2S)-binding protein
	**VVMO6_04404**	N.A.	8.605309291	8.495733105	8.604742788	7.188920684	7.882875566	8.253630594	iron ABC transporter substrate-binding protein
	**VVMO6_04408**	N.A.	8.835139482	8.86883098	9.034115854	5.462893536	7.367864104	7.207091752	aerobactin siderophore receptor IutA
	**VVMO6_03836**	N.A.	8.386635209	8.259714064	8.484380077	6.066827126	7.254687205	7.142797066	ferrichrome ABC transporter substrate-binding protein
	**VVMO6_03837**	N.A.	7.648393394	7.377129892	7.472485544	5.149119837	6.35495037	6.538326746	cell envelope biogenesis protein TonB
	**VVMO6_03838**	N.A.	5.881225728	5.83341208	5.936895673	3.752014387	4.684771772	4.734677136	biopolymer transporter protein ExbD
	**VVMO6_03839**	N.A.	7.113700124	6.947030191	7.165804174	4.870265932	5.731080976	5.90831929	hypothetical protein
	**VVMO6_03840**	N.A.	8.799086876	8.581712515	8.651167723	5.98980062	7.324023948	7.479395488	flagellar motor protein MotA
	**VVMO6_03841**	N.A.	8.247192252	8.365163087	8.406357145	5.550196942	7.025257207	6.935549176	biopolymer transporter TonB

* N.A. not available

(B) Oligonucleotides used in RT-qPCR

Name	Oligonucleotide sequence, 5' to 3'	Target gene
JUND_qRT_F	cagcgaggagcaggagtt	JUND
JUND_qRT_R	gagctggttctgcttgtgtaaat	JUND
KLF6_qRT_F	cacgagaccggctacttctc	KLF6
KLF6_qRT_R	ccagctctaggcaggtctgtt	KLF6
NLRP3_qRT_F	cacctgttgtgcaatctgaag	NLRP3
NLRP3_qRT_R	gcaagatcctgacaacatgc	NLRP3
EDN1_qRT_F	agacaaaccaggtcggagacca	EDN1
EDN1_qRT_R	tgggtcacataacgctctctgga	EDN1
TLR4_qRT_F	cctgcgtgagaccagaaag	TLR4
TLR4_qRT_R	ttcagctccatgcattgataa	TLR4
TLR8_qRT_F	gggagaatgaaggagtcatcttt	TLR8
TLR8_qRT_R	tcagcattgacgactgaagg	TLR8
TLR9_qRT_F	ggaccgggtcagtggtctct	TLR9
TLR9_qRT_R	agggctcaggatcaccagca	TLR9
IFNGR1_qRT_F	gggcagccatctgactccaa	IFNGR1
IFNGR1_qRT_R	tggagtgatacgagtttaaagcgatgc	IFNGR1
PYCARD_qRT_F	cttatcgcgagggtcacaa	PYCARD
PYCARD_qRT_R	caggaccttcccgtacagag	PYCARD
GAPDH_qRT_F	agccacatcgctcagacac	GAPDH
GAPDH_qRT_R	gcccaatacgaccaaatcc	GAPDH
04198_qRT_F	gctgcagtttgaagtcatgg	VVMO6_04198
04198_qRT_R	atcatcacgcgcagagttt	VVMO6_04198
04201_qRT_F	gtaccttggcggtgatgc	VVMO6_04201
04201_qRT_R	atactgcctgagcgtcgtg	VVMO6_04201
04202_qRT_F	gcaccacaagctcgtcttatt	VVMO6_04202
04202_qRT_R	catgcaccccctgcttat	VVMO6_04202
04207_qRT_F	gtggcatcgttgaagaaacc	VVMO6_04207
04207_qRT_R	aatcttgactcactggtg	VVMO6_04207
rrsh_qRT_F	gttggtgaggtaagggctca	*rrsH*
rrsh_qRT_R	gctgatcatcctctcagacca	*rrsH*
